# BamQuery: a proteogenomic tool to explore the immunopeptidome and prioritize actionable tumor antigens

**DOI:** 10.1186/s13059-023-03029-1

**Published:** 2023-08-15

**Authors:** Maria Virginia Ruiz Cuevas, Marie-Pierre Hardy, Jean-David Larouche, Anca Apavaloaei, Eralda Kina, Krystel Vincent, Patrick Gendron, Jean-Philippe Laverdure, Chantal Durette, Pierre Thibault, Sébastien Lemieux, Claude Perreault, Grégory Ehx

**Affiliations:** 1 Institute for Research in Immunology and Cancer (IRIC), Université de Montréal, Montreal, QC H3C 3J7 Canada; 2 Department of Biochemistry and Molecular Medicine, Université de Montréal, Montreal, QC H3C 3J7 Canada; 3 Department of Medicine, Université de Montréal, Montreal, QC H3C 3J7 Canada; 4 Department of Chemistry, Université de Montréal, Montreal, QC H3C 3J7 Canada; 5 Laboratory of Hematology, GIGA-I3, University of Liege, CHU of Liege, Liege, Belgium

**Keywords:** Immunopeptidome, Computational biology, Major histocompatibility complex, Tumor antigens

## Abstract

MHC-I-associated peptides deriving from non-coding genomic regions and mutations can generate tumor-specific antigens, including neoantigens. Quantifying tumor-specific antigens’ RNA expression in malignant and benign tissues is critical for discriminating actionable targets. We present BamQuery, a tool attributing an exhaustive RNA expression to MHC-I-associated peptides of any origin from bulk and single-cell RNA-sequencing data. We show that many cryptic and mutated tumor-specific antigens can derive from multiple discrete genomic regions, abundantly expressed in normal tissues. BamQuery can also be used to predict MHC-I-associated peptides immunogenicity and identify actionable tumor-specific antigens de novo.

**Supplementary Information:**

The online version contains supplementary material available at 10.1186/s13059-023-03029-1.

## Background

The immunopeptidome is the repertoire of MHC-I-associated peptides (MAPs) that represents in real-time the landscape of the intracellular proteome as it is molded by protein translation and degradation [1]. In recent years, immunopeptidomic data has been harvested to identify relevant and targetable tumor antigens (TAs). Indeed, MAPs deriving from mutations characterizing the neoplastic transformation (mutated TAs, also known as neoantigens) can be recognized by cytotoxic T cells and used as anti-cancer therapeutic targets [2].

The immunopeptidome is typically assumed to result from the degradation of canonical proteins, coded by exons and translated from known open-reading frames. This paradigm was challenged by proteogenomic studies using mass spectrometry (MS) analyses informed by genomic data such as RNA sequencing (RNA-seq). Indeed, these studies revealed that ~ 5–10% of MAPs derive from non-canonical (nc) regions of the genome, such as introns, non-coding RNAs (ncRNA), or endogenous retroelements (EREs), as well as from out-of-frame exonic translation [3–6]. Furthermore, a recent study showed that a significant fraction of MS peptide-spectrum matches assigned to canonical MAPs have better scores when attributed to ncMAPs, suggesting a greater contribution of the non-canonical regions to the immunopeptidome than previously estimated [7]. While most of the discovered ncMAPs are non-mutated [4, 8–12], many of them are found exclusively in cancer cells and attract attention as (1) they can be immunogenic in vitro as well as in vivo; (2) they are more numerous in the immunopeptidome of malignant cells than mutated TAs, and (3) several non-coding TAs are widely shared between cancer patients whereas mutations mainly generate private antigens [13, 14]. Identifying ncMAPs and actionable TAs has raised three challenges immunologists often address inconsistently.

The first is the attribution of an exact RNA expression to MAPs. Because immunopeptidomic identifications by MS tend to miss lowly abundant MAPs and to be poorly reproducible, the expression of the TA candidates in RNA-seq data is preferably measured to assess their tumor specificity [8–12]. Typically, proteogenomic pipelines quantify MAP RNA expression by estimating their parental transcript expression with conventional tools such as Kallisto [15] or HTSeq [16]. However, such tools cannot be used for ncMAPs which often derive from unannotated transcripts. Furthermore, such approaches do not consider that MAPs (8–11 residues) could derive from multiple regions of the genome due to the degeneracy of the genetic code and the existence of numerous paralogs/orthologs. Therefore, studies failing to consider all genomic regions susceptible to generating a given MAP would underestimate its RNA expression. The second is the attribution of a biotype to MAPs (a biotype corresponds to the functional annotations of each genomic region, e.g., protein-coding exon, intron, ERE, ncRNA, pseudogene). The presence of multiple genomic regions that can produce identical MAPs, and exhibit different biotypes, can lead to the misidentification of their origins. For instance, a MAP may be attributed to an ERE origin, while a canonical exon could also generate it through out-of-frame translation, possibly with a greater probability. The third challenge is to prioritize TAs. Ideally, TAs should be immunogenic and specifically expressed (or overexpressed) by malignant cells [17]. Because RNA expression is a reliable proxy of the MAP presentation probability [9, 18], RNA-seq data of tumor and normal samples are powerful tools to perform TA prioritization. Tumor specificity is typically evaluated by comparing MAP RNA expression between paired tumor and normal tissue samples. However, considering MAP RNA expression in medullary thymic epithelial cells (mTECs) should have a distinct advantage. Indeed, it should be a good predictor of immunogenicity because mTEC MAPs induce central immune tolerance [17, 19]. However, for the reasons mentioned above, reliable comparison of MAP RNA expression between tumors, their paired normal samples, and mTECs requires considering all their possible genomic regions of origin.

To address these challenges, we developed BamQuery, an annotation-independent tool that enables the attribution of an exhaustive RNA expression profile to any MAP of interest in any RNA-seq dataset of interest.

## Results

### Exhaustive capture of MAP RNA expression

**Fig. 1:**
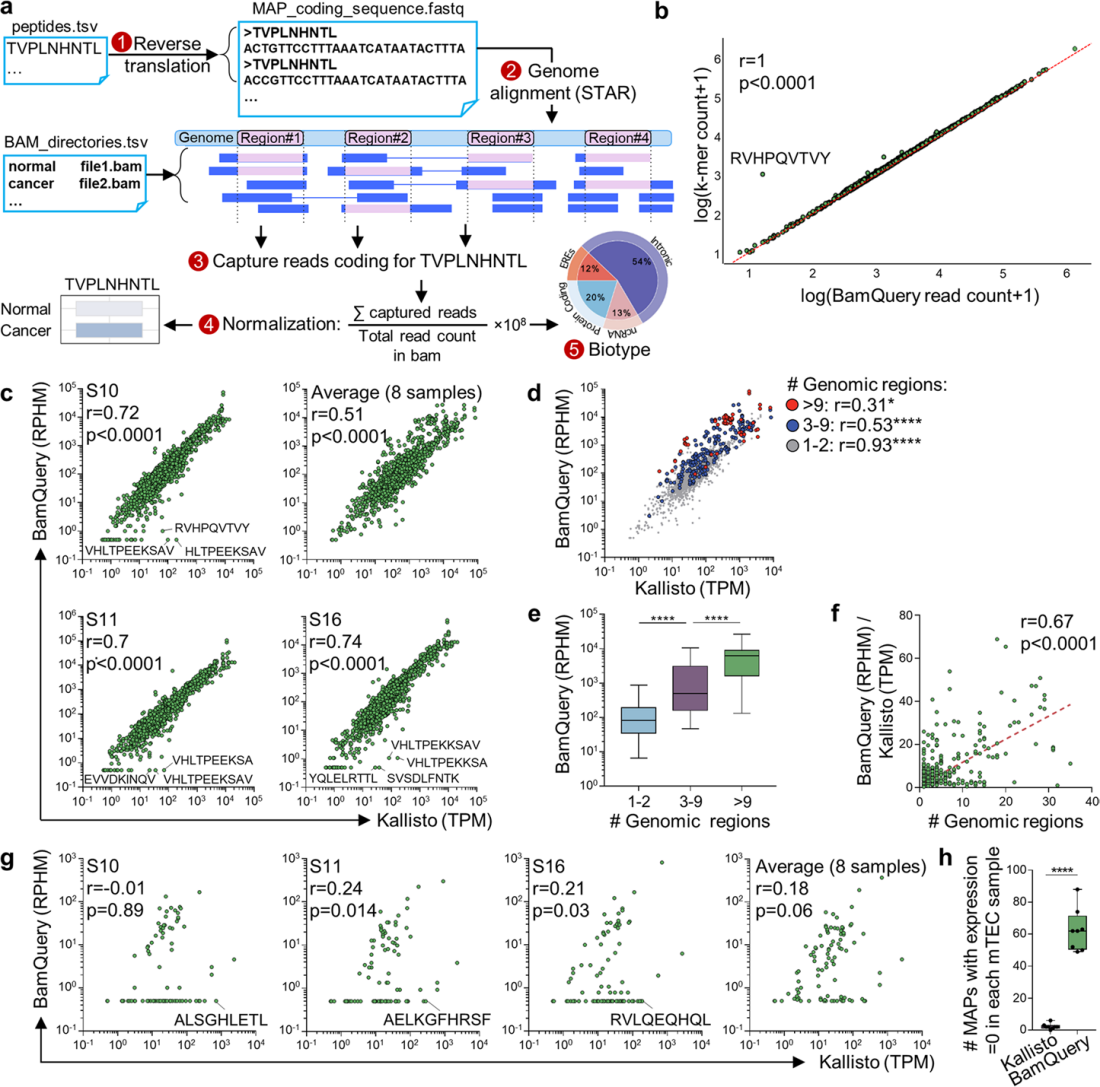
Exhaustive capture of MAPs RNA expression. **a** Overview of the BamQuery approach to measuring MAP RNA expression levels. **b** Pearson’s correlation between BamQuery-acquired read counts and Jellyfish’s K-mer counts for canonical nonamer MAPs (*n* = 1211) from the HLA Ligand Atlas (present in at least 20 different tissues) in eight mTEC samples. **c** Pearson’s correlation between BamQuery’s (in RPHM) and Kallisto’s (in TPM) quantifications of 1702 MAPs from the HLA Ligand Atlas in 8 mTEC samples. Because Kallisto does not perform direct quantifications of MAPs’ RNA expression, the expression of their gene of origin was used as a surrogate. A value of 0.5 was added to each RPHM or TPM value to enable visualization on a logarithmic axis. Correlations for three representative samples and the average of the eight samples are shown. **d** Segregation of MAPs based on their number of coding regions and re-computation of correlations for the average of eight mTEC samples. **e** Box plots of the average expression of MAPs across the eight mTECs, segregated based on the number of coding regions. **f** Correlation between the ratio, for each MAP, between the BamQuery and the Kallisto quantification (average of eight mTECs), as a function of the number of coding regions. **g** Correlation between BamQuery’s (in RPHM) and Kallisto’s (in TPM) quantifications of 108 mutated MAPs in eight mTEC samples (analysis performed as in panel **c**). **h** The number of mutated MAPs having an expression = 0 according to Kallisto or BamQuery is reported in each of the eight tested mTEC samples (dots)

We designed BamQuery to evaluate MAP RNA expression independently of annotations for two reasons. Firstly, no annotations are available for MAP-coding transcripts located in intergenic regions. Secondly, genomic annotations cover vast regions unlikely to accurately represent the local RNA expression of an 8–11 residue peptide (especially for ncMAPs deriving from introns, Additional file 1: Fig. S1a). Due to the small size of MAP-coding sequences (MCS, 24–33 nucleotides), counting the RNA-seq reads containing each MCS able to code for a given peptide is the most thorough and least error-prone method to evaluate MAP RNA expression. To make BamQuery readily available, it had to work on a broadly used data format. Given that querying MCS in fastq files is time-consuming (> 1 min / MCS), we designed BamQuery to work on bam files in five steps (Fig. 1a, and “Methods”): (1) reverse translation of each MAP into all possible MCS; (2) mapping of MCS to the genome using STAR [20] to identify those having perfect matches with the reference and attribute them a genomic location. At this step, we also include to the reference genome the mutations from the dbSNP annotations [21] to enable the mapping of mutated sequences; (3) counting of the primary RNA-seq reads encompassing exactly the MCS at their respective location and sum read counts of each MAP across locations; (4) normalization of the read count of each MAP by the total primary alignment read count of the sample and multiplication by 1 × 10^8^ to yield read-per-hundred-million (RPHM) numbers and (5) attribution of biotypes to MAPs based on the reference annotations overlapping the various expressed (RPHM > 0) regions.

To test BamQuery, we collected robustly validated MAPs derived from benign tissues reported in the HLA Ligand Atlas [22] (1702 canonical MAPs shared across at least 20 tissues, Additional file 1: Fig. S1b, c) and queried them in the transcriptome of eight mTEC samples sequenced previously [10, 23]. As a control, we used the primary reads contained in the mTEC bam files previously aligned with STAR to generate a database of 27-nucleotide-long k-mers (reads chunked into shorter sequences) using Jellyfish [24], a tool that counts k-mer occurrences in the primary read sequences (“Methods”). Importantly, we preferred designing BamQuery to work on bam files instead of Jellyfish k-mer files of original fastq files because of the elevated disk space that k-mer databases require (4 databases would be needed per sample to query MAPs of 8 to 11 amino acid length) and because such databases would not provide information about the genomic region of the queried MCS.

We queried this 27-nucleotide-long k-mer database for all possible 27-mer-MCSs encoding 9-amino acid-long MAPs (1211/1702). The comparison of total read counts between BamQuery and total k-mer occurrences for each MAP showed a correlation equal to 1, demonstrating the exhaustivity of BamQuery (Fig. 1b). Importantly, the main outlier in this correlation was the RVHPQVTVY peptide, deriving from the HLA-DRB3 gene. Previously, the STAR aligner was shown to have poor performance in hypervariable genomic regions such as HLA genes [25]. Consequently, this outlier results from the limited capacity of STAR to map MCS to the HLA-DRB3 gene when performing the BamQuery analysis. A more detailed comparison between MCS counts given by BamQuery and k-mer counts in the database also showed an excellent correlation, except for the MCS coding for the RVHPQVTVY peptide (Additional file 1: Fig. S2a).

Currently, quantifying canonical MAP RNA abundance is performed with conventional tools such as Kallisto and HTSeq [15, 16]. Kallisto, which provides results similar to other tools and boasts the fastest computing speed [26], was selected for comparison with BamQuery. Similar to other conventional tools, Kallisto counts the number of reads overlapping a large genomic region corresponding to pre-determined coordinates, such as coding genes. The expression of a MAP-coding gene is then used as a proxy to attribute an RNA expression to the tested MAP. In contrast with BamQuery, using conventional tools does not enable the quantification of the reads directly coding for a specific MAP. Good correlations (0.7–0.8) between Kallisto and BamQuery were observed for each of the eight mTEC samples tested (Fig. 1c). A good correlation (0.51) was also obtained when computing the average of expression across the eight samples. However, when recomputing correlations on subsets of MAPs segregated based on their number of coding regions detected by BamQuery, we observed lower correlation values for MAPs originating from 3 to 9 or more than 9 regions (Fig. 1d). These MAPs presented significantly higher expression than those coded by few (1–2) regions (Fig. 1e). Furthermore, the ratio between BamQuery’s RPHM and Kallisto’s TPM values increased significantly with the number of coding regions (Fig. 1f). Similar results were obtained when using a different dataset of MAPs, selected based on their possibility to be coded by ERE regions, and therefore deriving from more regions on average than our initial dataset (Additional file 1: Fig. S2b-d). Finally, we assessed whether similar results could be obtained with another transcript abundance quantification tool, HTSeq [16]. BamQuery vs. HTSeq correlations were lower than those obtained with Kallisto (~ 0.6, Additional file 1: Fig. S2e). Critically, HTSeq did not detect expression for six genes encoding MAPs, while BamQuery found significant expression for these peptides. Altogether, these data show that BamQuery captures more RNA expression than Kallisto or HTSeq for highly expressed MAPs deriving from multiple genomic regions.

We found a second type of divergence between BamQuery and Kallisto. In specific samples (representative samples S10-11–16 are shown in Fig. 1c), many MAP-coding transcripts were classified as unexpressed by BamQuery and highly expressed by Kallisto. Upon manual examination in the IGV genome browser [27], we observed that these MAPs overlapped annotated mutations in the dbSNP database and that the considered mTEC sample did not express the allele necessary to enable the presentation of the peptides (Additional file 1: Fig. S3). Therefore, we reasoned that BamQuery should outperform Kallisto substantially for the detection of mutated MAPs. To test this hypothesis, we quantified the expression of 108 MAPs deriving from non-synonymous benign germline mutations in protein-coding genes published by our group before [28]. The expression of the gene of origin, detected by Kallisto, was compared to BamQuery’s quantifications. Poor correlations (− 0.04 to 0.24) were obtained for individual samples, and the average expression across all mTEC samples (Fig. 1g). The number of MAPs with an expression equal to zero in each mTEC sample was dramatically greater when detected with BamQuery than with Kallisto (Fig. 1h). Manual examination in IGV of outlier MAPs showed that BamQuery’s non-detection reflected mutated MAPs unable to be coded by their mTEC sample since wild-type reads were expressed (Additional file 1: Fig. S4).

Finally, we tested the speed of BamQuery when analyzing the expression of the 1702 canonical and 724 non-canonical MAPs in the eight mTEC samples. We measured the execution time in function of the total number of MCS analyzed for random selections of 1, 2, 3, 4, 5, 10, 15, 20, 30, 50, 70, or 100 MAPs among our dataset (10 random selection were made for each number, Additional file 1: Fig. S5a). BamQuery required a median time of 37 and 92 s to evaluate the expression of canonical and non-canonical MAP, respectively (Additional file 1: Fig. S5b-left panel). This translates to an average of approximately 4.6 and 11.5 s to assess each canonical and non-canonical MAP, respectively, within a single sample. The disparity in processing time can be explained by the fact that non-canonical MAPs produce higher numbers of MCS than canonical peptides (Additional file 1: Fig. S5b, right panel). To investigate the effect of the number of cores on BamQuery’s runtime, we analyzed 100 randomly selected canonical and non-canonical MAPs using 4, 8, 16, or 32 cores. As expected, the results showed that using 32 cores significantly reduced the runtime by two- to three-fold compared to using 4 cores (Additional file 1: Fig. S5c). We also tested the performance of BamQuery with 32 cores while analyzing 1000 MAPs (90% canonical and 10% non-canonical, as is expected in a typical immunopeptidome) in 2489 normal samples from GTEx (spread across 50 different tissues). BamQuery required only 30 s per sample, enabling the analysis of a full immunopeptidome in numerous benign samples in less than 24 h (22.8 h in total). Overall, these results highlight the speed, accuracy, versatility, and superiority of BamQuery over other approaches.

### RNA expression level as a proxy for protein translation and MAP generation

**Fig. 2:**
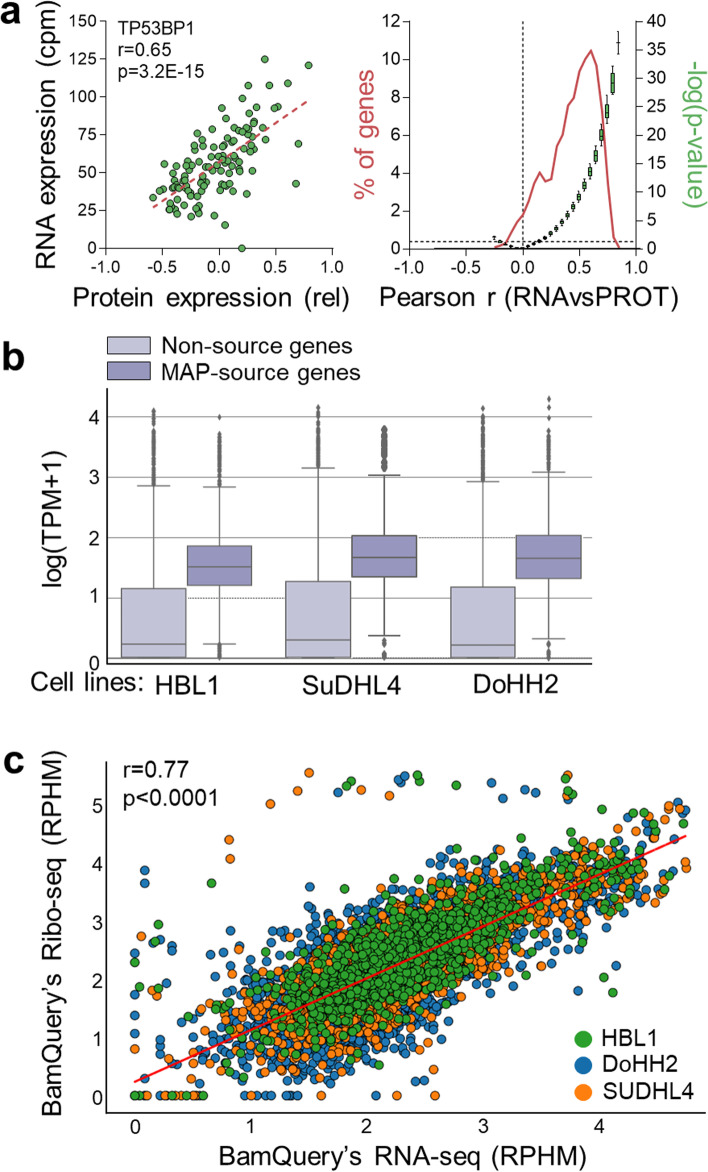
RNA expression level as a proxy for protein translation and MAP generation. **a** The abundance of 3586 proteins (assessed in [30]) was correlated to their corresponding transcript abundance (obtained with Kallisto from public TCGA RNA-seq data) across 115 high-grade serous ovarian cancer patients for which matched proteomic and transcriptomic data were available. Left panel: representative correlation obtained for the TP53BP1 gene across 115 patients. Right panel: distribution of the frequency of genes (*n* = 3586) and *p*-values among different categories of correlation values (*r*), incremented by 0.05 from − 0.25 to + 0.9. **b** Transcription expression level distribution of MAP source and non-source genes in three DLBCL cell lines. **c** Correlation between BamQuery RNA expression and BamQuery Ribo-seq expression (in RPHM) of 6833 MAPs detected in 3 DLBCL cell lines

Many studies have reported a strong positive correlation between RNA expression levels and the generation of MAPs [5, 8, 9, 18, 29]. We compared protein and RNA expression levels to demonstrate further that a high RNA expression increases the probability of protein translation and, thereby, MAP generation. These analyses were made with a dataset of 3586 proteins examined by the TCGA group in high-grade serous ovarian cancer patients [30]. The correlation between protein and RNA expression across the 115 patients for which paired RNA-seq data were available showed that only ~ 15% of transcripts/protein couples were not positively and significantly correlated (Fig. 2a).

To explore the relation between transcriptome and immunopeptidome, we took advantage of a dataset of three diffuse large B cell lymphoma cell lines [5] for which matched RNA-seq, Ribo-seq, and immunopeptidomic data are available. Because no quantitative data were available for MAP abundance, we compared the abundance of transcripts coding, or not, for MAPs. Transcripts that were sources of MAPs presented an RNA expression significantly higher than the non-source transcripts (Fig. 2b), supporting the predictive capacity of RNA expression on MAP generation probability. Ribo-seq produces a detailed map of active cell translation events. The reads identified by this sequencing method can be aligned on the genome to create a bam file for analysis with BamQuery. For each of the three cell lines, we correlated the RNA-seq and Ribo-seq quantifications for each MAP identified by MS (Fig. 2c). This evidenced an excellent correlation between the two methods, showing that RNA expression is a good proxy for the translation probability of MAPs.

### New insights into the immunopeptidome biology

Next, we explored the biological features of the immunopeptidome by evaluating the expression of the 1702 canonical MAPs from the HLA ligand atlas along with 724 MAPs previously reported as non-canonical in normal tissues, including mTEC samples [31] and tissues from GTEx [32] (Additional files 2 and 3). BamQuery attributed a genomic location to 100% MAPs: among canonical MAPs, all originally annotated genes were attributed to their respective MAP by BamQuery, and among an extensive list of well-annotated ncMAPs [9], the originally annotated genomic location was re-located by BamQuery with an accuracy of 100%.

**Fig. 3:**
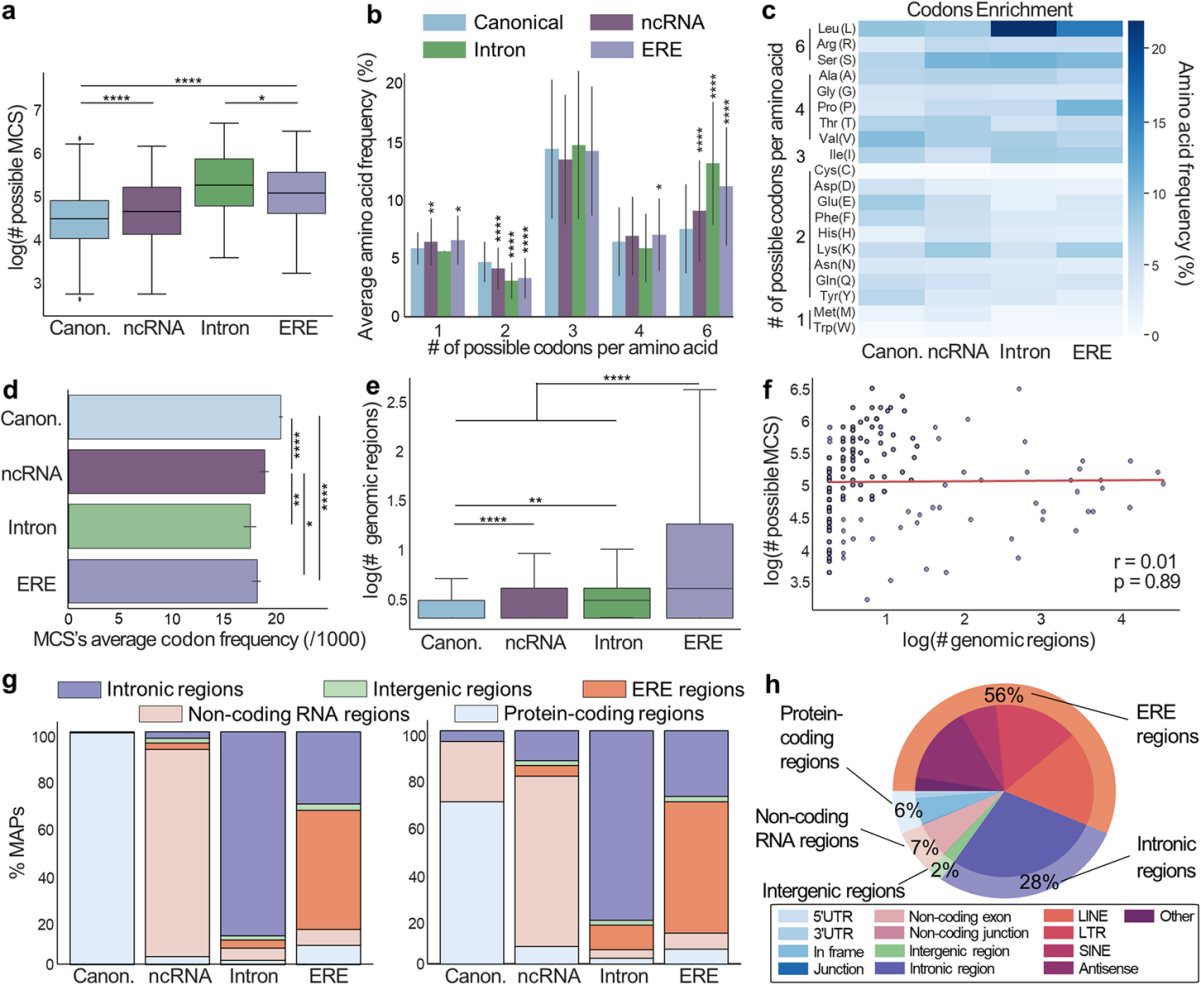
New insights into the immunopeptidome biology. **a–h** Published MAPs reported as canonical (*n* = 1702) and non-canonical (ncRNA (*n* = 378), intronic (*n* = 114), and EREs (*n* = 232)) were searched with BamQuery in GTEx tissues and mTEC bam files in unstranded mode (GTEx data being unstranded) with genome version GRCh38.p13, gene set annotations release v38_104, and dbSNP release 151. Panels **a, e, f, g** were generated with the comparison of 9-mers only (*n* = 1211 canonical, *n* = 207 ncRNA, *n* = 68 intronic, *n* = 157 EREs) to prevent possible biases introduced by variable frequencies of 8/10/11-mers among the compared groups. Figures **b, c, h** were generated with the complete MAP dataset (*n* = 1702 canonical, *n* = 378 ncRNA, *n* = 114 intronic, *n* = 232 EREs). Mann–Whitney *U* test was used for indicated comparisons (*p < 0.05, **p < 0.01, ***p < 0.001, ****p < 0.0001). **a** Number of possible MCS after reverse-translation of indicated MAP groups. **b** Average frequency (%) of amino acids encoded by the indicated number of synonymous codons in indicated MAP groups. **c** Heat map of amino acid frequency in indicated MAP groups. **d** Mean of the MCS average usage frequency of codons (among 1000 codons located in human reference protein-coding sequences) encoding each of the 20 amino acids of indicated MAP groups. Codon frequencies were obtained from the codon usage database (http://www.kazusa.or.jp/codon/). **e** Number of MCS genomic locations able to code for the indicated MAP groups. **f** Pearson’s correlation between the number of possible MCS after reverse translation vs. the number of MCS genomic locations able to code for the assessed ERE MAPs. The red line is a linear regression. **g** Percentage of MAPs attributed to indicated biotypes by BamQuery based on the best guess (left) or EM-established (right) biotype ranks, and the genomic regions expressed in GTEx tissues and mTECs. The *X*-axis indicates the biotype reported in the original study (groups). For clarity, BamQuery biotypes were summarized into five general categories: protein-coding regions, non-coding RNAs, EREs, intronic and intergenic. **h** Percentage of the most likely biotype attributed by BamQuery to EREs MAPs

Comparing all 9-mers together (to prevent biases due to differences of length proportions), a higher number of possible MCS (total number of MCS after reverse translation) was found for non-canonical vs. canonical MAPs, especially for those mapping to introns and EREs (Fig. 3a). We investigated whether this bias could be linked to the degeneracy of codons. We found that residues encoded by six synonymous codons (R/L/S) were enriched in intron- and ERE-derived MAPs, with leucine being the most enriched (Fig. 3b,c). These differences were not observed for peptides that cannot be presented by MHC molecules, suggesting that a MAP-specific mechanism explains these results (Additional file 1: Fig. S6a-b). Previously, we observed that MAP source transcripts use rare codons more frequently than transcripts that do not generate MAPs [4]. Therefore, we hypothesized that ncMAPs would use rare codons more frequently than canonical MAPs. Coherent with this assumption, we found that the genomic codon frequency of residues encoded by six synonymous codons (R/L/S) was, on average, lower than those encoded by lower numbers of synonymous codons (Additional file 1: Fig. S6c). Furthermore, codons of ncMCS presented a lower genomic frequency than codons of canonical MCS (Fig. 3d). As rare codons are rate limiting for protein synthesis [33–35] and as MAPs frequently derive from defective ribosomal products (DRiPs) generated by alterations of protein synthesis rate [36], our data suggest that DRiPs contribute more to the generation of ncMAPs than to canonical ones.

Next, we analyzed the relation between the number of possible MCS per MAP (i.e., diversity of synonymous codons) and the number of genomic regions able to code for a given MAP. Canonical MAPs are primarily derived from a reduced number of genomic regions, with 63% originating from a single genomic location. In contrast, ncMAPs could derive from multiple regions, with only 43% originating from a single genomic location (Fig. 3e, Additional file 1: Fig. S6d). ERE MAPs presented the greatest numbers of possible regions, in agreement with their repeated nature (up to 35,343 potential regions). However, their number of potential MCS did not correlate with the number of possible locations, showing that amino acid residue composition cannot be used to predict the number of possible regions of origin (Fig. 3f).

Finally, given the multiplicity of possible regions of origin, we computed the most likely biotype of each MAP. For this, we used machine learning (expectation–maximization algorithm) to rank the biotypes (in-frame, intron, ERE, etc.) as a function of their likelihood of generating the reads covering them across the whole set of GTEx tissues. In general, canonical in-frame transcripts are more likely translated than non-canonical ones. For this reason, BamQuery’s best guess automatically ranks as “in-frame” any MAP having at least one in-frame canonical origin, which was the case for all canonical MAPs from our dataset (Fig. 3g, left panel). BamQuery can also attribute biotypes based only on the likelihood ranks (considering the number of reads overlapping each transcript). In this case, ~ 26% of canonical MAPs were assigned with a greater probability to ncRNAs (Fig. 3g, right panel). Intron and ncRNA MAPs were predicted to belong mainly to their identified biotype (81 and 73%) (Additional file 1: Fig. S6e). However, only 56% of ERE-derived MAPs were estimated to derive from EREs, and 6% of them could derive from canonical regions (5% in-frame) (Fig. 3h). Altogether, these data show that many published MAPs could be mislabeled either as canonical or non-canonical.

### Single-cell proteogenomic analyses

High-throughput single-cell RNA sequencing (scRNA-seq) enables the examination of individual cells’ transcriptome [37, 38]. Therefore, we sought to perform single-cell analyses using BamQuery. Given the end-bias of the Chromium library design typically used in scRNA-seq, we evaluated whether read coverage would allow BamQuery analyses of canonical and non-canonical MAPs in cancerous [39] and normal [40] lung tissues scRNA-seq data. As expected, reads showed a bias toward the 3′ end of the canonical genes (Additional file 1: Fig. S7a). However, the coverage extended far from the 3′ end, in agreement with a report detecting mutations in various regions of the gene body [41]. We also found a surprisingly high (~ 50% of reads) and homogeneous read coverage in introns and ERE regions, in agreement with previous reports [42, 43], suggesting that BamQuery could detect expression for ncMAPs in scRNA-seq.

**Fig. 4:**
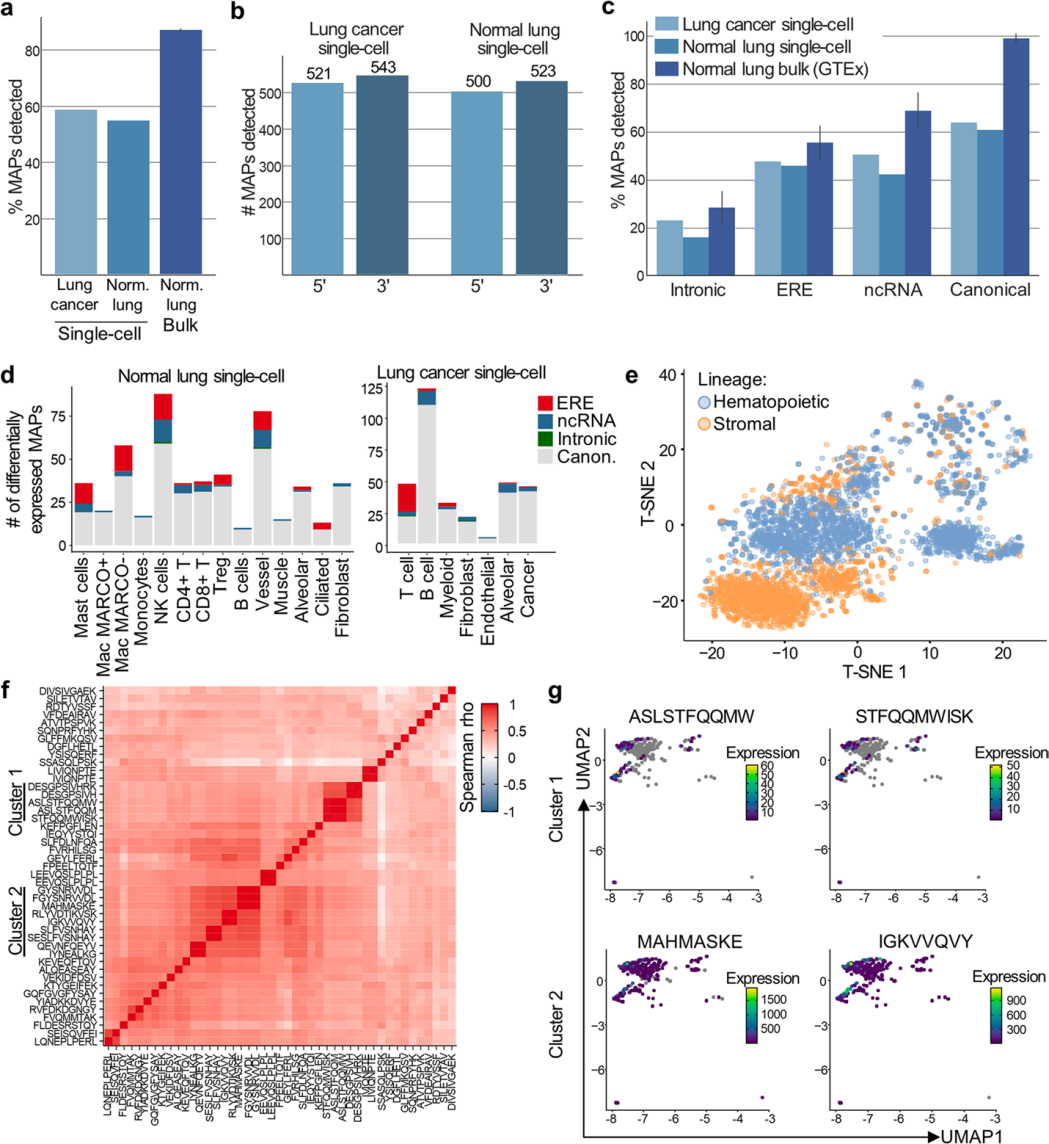
Single-cell proteogenomic analyses. **a–g** Canonical (*n* = 1702) and non-canonical MAPs (ncRNA (378), intronic (114), and EREs (232)) were searched with BamQuery in bam files of scRNA-seq of normal and cancerous lung samples in single-cell in stranded mode with genome version GRCh38.p13, gene set annotations release v38_104, and dbSNP release 151. **a** Median percentage of MAPs detected in normal and cancerous lung scRNA-seq, as well as in bulk RNA-seq samples of normal lungs from GTEx (*n* = 150). **b** Number of canonical MAPs located in the 5′ (first half of the transcript) or 3′ (second half of the transcript) region of the transcript detected in indicated scRNA-seq datasets. **c** Median percentage of indicated MAP groups detected in normal and cancerous lung scRNA-seq, as well as in bulk RNA-seq samples of normal lungs from GTEx. **d** Number of MAPs identified as differentially expressed by the different populations of cells in the normal lung (left panel) or cancerous lung (right panel). The originally reported biotype of the MAPs is indicated by the color code. **e** TSNE analysis of the hematopoietic (blue) and stromal (orange) cells from the normal lung based on their MAP expression. **f** Heatmap showing the co-expression (spearman rho, color bar) of MAPs overexpressed by lung cancer cells (rows vs. columns). Two clusters of MAPs are highlighted on the left side of the heatmap (cluster 1 and cluster 2). **g** TSNE showing the expression of MAPs (color bar) from cluster 1 (higher panel) or cluster 2 (lower panel). Grey color indicates the null expression of a MAP in a cell

BamQuery detected expression for 50–60% of the canonical and non-canonical MAPs (Additional file 2) in scRNA-seq, while 86% were found in bulk RNA-seq of GTEx lung samples (Fig. 4a). This lower number of MAPs expressed in single-cell data can be ascribed to lower read coverage and did not hamper the feasibility of scRNA-seq analyses. Indeed, despite the biased read coverage toward the 3′ end of transcripts, the read coverage of the 5′ end was sufficient to enable the detection of at least one read coding for all canonical MAPs (Fig. 4b). Also, the expressed rate of intronic and ERE MAPs in scRNA-seq data was more comparable to bulk RNA-seq data than canonical MAPs (Fig. 4c). This likely results from the more homogeneous read coverage observed in non-coding than in coding regions (Additional file 1: Fig. S7a).

Therefore, we explored the patterns of MAP expression in normal and malignant lungs. Differential expression analysis showed that 12.86% (186/1446) and 16.46% (248/1506) of MAPs presented cell type-specific expression profiles in normal and malignant samples, respectively (Fig. 4d and Additional files 4 and 5). Several differentially expressed MAPs derived from genes having cell type-specific functions, such as YTAVVPLVY in B cells (immunoglobulin J polypeptide), STFQQMWISK in muscle cells (Beta-actin-like protein 2), and FLLFPDMEA in macrophages (complement C1q B chain) (Additional file 1: Fig. S7b). Importantly, upon manual validation, we observed that many cells of the examined population did not express the MAPs found to be differentially expressed. The SSASQLPSK ERE MAP is shown as an example in Additional file 1: Fig. S7c-e where multiple cells did not express the peptide in both populations (cancer and alveolar cells) between which it was differentially expressed. While this phenomenon, known as zero inflation, has a likely biological origin [44], it suggests that the expression of MAPs should be considered at the cell cluster level and that specialized tools, such as MAST [45] that we used for the differential expression analysis above, should be used to characterize their expression.

To further assess the reliability of MAP expression, we re-clustered the normal lung dataset based uniquely on MAP expression. This provided a clear separation of the hematopoietic and stromal compartments (Fig. 4e, Additional file 1: Fig. S8a) and allowed the clustering of specific cell populations such as alveolar cells or the monocytes and macrophages (Additional file 1: Fig. S8b, c). Strikingly, most MAPs identified as differentially expressed in the normal lung dataset had an expression restricted to either the hematopoietic or stromal lineages, showing a clear dichotomy between these two compartments in terms of MAP expression (Additional file 1: Fig. S8d).

Given the growing interest in TAs shared between tumor cells, we assessed the clonality of 45 MAPs whose coding sequences were overexpressed by cancer cells through co-expression analyses. This highlighted two clusters of MAPs co-expressed in lung cancer cells (Fig. 4f) with different expression profiles (Fig. 4g). A limited number of cancer cells expressed MAPs of cluster 1, whereas MAPs of cluster 2 were ubiquitously expressed, making them more desirable immunotherapeutic targets. Importantly, a conventional clustering (UMAP + k-nearest neighbors analysis) of the lung cancer cells based on their canonical gene expression provided five different clusters (Fig S8e). All MAPs from cluster 1 in Fig. 4f were overexpressed by the new cluster 2 whereas MAPs from cluster 2 (Fig. 4f) were associated with new clusters 1 and 2 (Additional file 6), confirming the co-expression pattern of the MAPs in tumor cells.

Finally, we examined the possibility of using BamQuery to investigate the tumor specificity of mutated TAs. For this, we performed a UMAP clustering of the total lung cancer sample (normal cells + malignant cells) based on their canonical gene expression and projected the expression of mutated TAs, analyzed with BamQuery, on this UMAP. This showed that among the 393 neoantigens analyzed (dataset assembled from publications and public databases [11, 46, 47]), an RNA expression was found for eleven of them, and one (RLLCPPARA, a melanoma neoantigen [48]) presented a tumor-specific expression (Additional file 1: Fig. S8f-h).

These data demonstrate the capacity of BamQuery to perform scRNA-seq analyses and evidence its potential to assess TAs intra-tumoral heterogeneity and tumor specificity.

### MAP expression is underestimated in healthy tissues

**Fig. 5:**
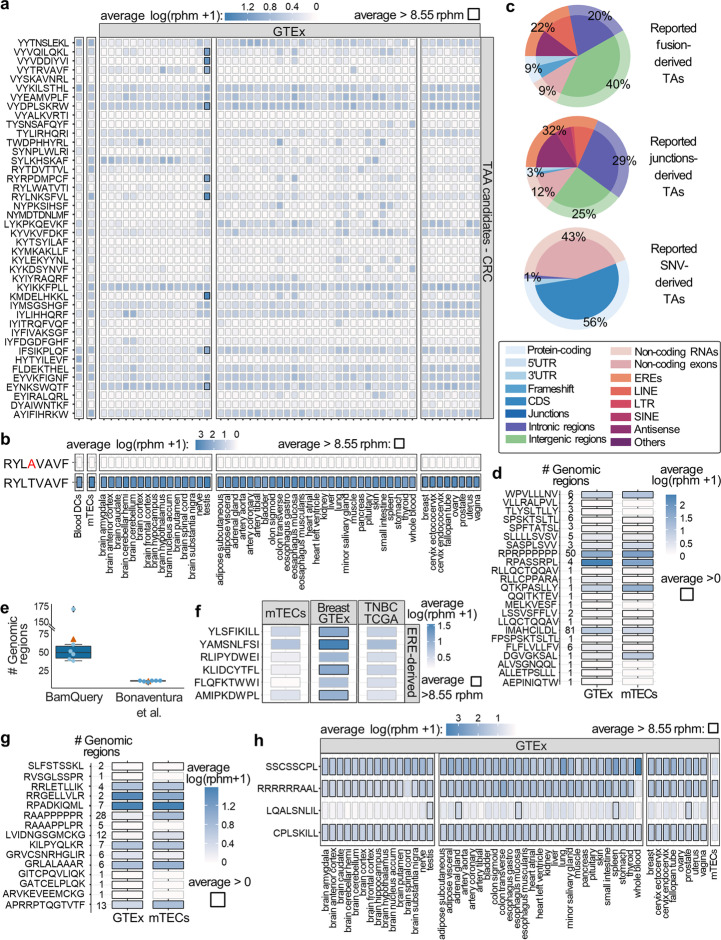
Underestimated MAP expression in healthy tissues. **a–h** Published human colorectal cancer (CRC) TAAs, mutated TAs, ERE-derived TSAs, proteasomal splicing peptides, and Epstein–Barr virus (EBV) MAPs were searched with BamQuery in the GTEx tissues (*n* = 12–50 / tissue), mTECs (*n* = 11), and DCs (*n* = 19) bam files in unstranded mode with genome version GRCh38.p13, gene set annotations release v38_104 and dbSNP release 155 (except for the search for mutated TAs (**d**) where dbSNP was not considered, dbSNP = 0). **a** Heatmap of average RNA expression of published CRC TAAs in indicated tissues. Boxes in which a peptide has an average rphm > 8.55 are highlighted in black. **b** Heatmap of average RNA expression of the CRC mutated TA RYLAVAAVF and its wild-type RYLTVAAVF in indicated tissues. **c** Percentage of the most likely biotype attributed by BamQuery to published fusions, junctions, and SNVs-derived TAs. **d** Heatmap of average RNA expression of published mutated TAs (*n* = 23) in indicated tissues. The number of genomic locations expressed is presented on the left. **e** Number of genomic locations at which the expression of the ERE TSAs was assessed by BamQuery vs. the original study. Light blue dots represent each assessed MAP, and the orange triangle represents the average. **f** Heatmap of average RNA expression of the EREs-derived TSAs in mTECs, normal breast tissues from GTEx (*n* = 50), and triple-negative breast cancer samples from TCGA (*n* = 158). **g** Heatmap of average RNA expression of published proteasomal splicing MAPs (*n* = 99) in indicated tissues. The number of genomic locations expressed is presented on the left. **h** Heatmap of average RNA expression of EBV MAPs in indicated tissues

Given the ability of BamQuery to capture MAP RNA expression exhaustively, we evaluated the genomic origin of previously reported MAPs. First, we examined 1062 colorectal cancer (CRC) TAs identified by their presence and absence from the immunopeptidome of malignant and paired benign cells, respectively, and reported by Hirama et al. [49]. To evaluate their probability of being presented by normal cells, we queried them in 3 datasets: GTEx, mTECs, and sorted dendritic cells (DCs) [50, 51] (Additional file 3). Four percent of TAs presented an expression < 8.55 RPHM (minimum expression required to result in a probability of > 5% of generating a MAP [9]) in all normal tissues, except for testis. These antigens can be classified as cancer-testis antigens (CTAs) (Fig. 5a). Strikingly, among the 7 TAs previously reported to be lowly expressed at RNA level in normal matched tissues, BamQuery revealed that only one (KYLEKYYNL) presented a low expression across all peripheral tissues. Finally, the only mutated TA reported by Hirama et al. (RYLAVAAVF) was found to be genuinely cancer-specific: RYLAVAAVF-coding RNA was absent in normal tissues, while its unmutated counterpart was highly expressed (Fig. 5b).

Second, we wondered whether all mutated TAs would be as tumor-specific as expected. We analyzed 45 8–11-amino-acid = long mutated peptides (7 from gene fusions, 28 from aberrant splice junctions, and 10 from single-nucleotide variations, SNV) reported as tumor-specific in medulloblastoma (no RNA expression in GTEx) [52]. BamQuery could attribute a genomic location to 39 of them and mapped 7/10 SNV peptides to their reported genes (Additional file 1: Fig. S9a). Unexpectedly, BamQuery attributed non-discontinued (“unspliced”) expressed genomic locations to 82% of fusion and spliced peptides, evidencing that non-mutated (and mostly non-canonical, Fig. 5c) genomic regions could also code for those peptides. Overall, only 26 of 45 TAs presented low expression in normal tissues (Additional file 1: Fig. S9b) including all detected SNV-derived peptides. Therefore, we wondered whether mutated MAPs reported as cancer-specific in previous publications and public databases [11, 46, 47] would be verified as such by BamQuery. From 393 mutated TAs (Additional file 7), 23 (5.85%) were highly expressed in normal tissues, where 25% of the peptides have more than five non-mutated genomic locations perfectly matching their MCS (Fig. 5d).

Third, we examined six ERE-derived MAPs reported as TAs in triple-negative breast cancer [53]. These TAs were identified by comparing the expression of a pre-determined list of human endogenous retroviruses (HERV-K) between normal and tumor samples. The existence of MAPs deriving from HERV sequences overexpressed by cancer cells was validated by MS. In the original report, the only possible sources considered for these MAPs were the HERVs in the study list and the canonical proteome, which was checked for the absence of the HERV MAP sequences. An average of eight different genomic locations (HERV sequences) were reported for each TA. In contrast, by interrogating the whole genome, and therefore without depending on specific HERV annotations, BamQuery identified ~ 66 expressed regions for each HERV TA, in agreement with the repetitive nature of the HERV-K sequences (Fig. 5e, Additional file 1: Fig. S9c). Hence, these MAPs showed higher expression in normal breast samples than in cancer samples (Fig. 5f). These results highlight the importance of considering all genomic locations able to generate a given MAP when measuring RNA expression to report TA.

Fourth, we evaluated whether BamQuery would detect non-discontinued genomic locations and RNA expression for MAPs supposedly impossible to be expressed by the human genome. We first examined 99 MAPs presumed to derive from proteasomal splicing (post-translational recombination of protein fragments) [54]. Fifteen could be generated by expressed regions (Fig. 5g), suggesting a possible misclassification of these peptides. Finally, considering the tight link between Epstein–Barr virus (EBV) infection and autoimmune disorders such as multiple sclerosis [55], we examined the expression of 511 EBV-derived MAPs in the IEDB database. Four could be coded by the human genome and were expressed at high levels by normal tissues (Fig. 5h). Interestingly, one of them, CPLSKILL, can be presented by HLA-B8 molecules, an allele frequently associated with autoimmune disorders [56].

Finally, we sought to evaluate whether BamQuery-based evaluation of TA expression might help predict potential off-target toxicities. We gathered a dataset of 12 MAPs targeted in phase I cancer immunotherapy trials where the occurrence of autoimmune toxicities was assessed. Then, we queried the expression of these TAs in the normal tissues of GTEx (Additional file 1: Fig. S9d). Five MAPs presented substantial expression in multiple tissues: WT1, CEA, PMEL, Titin, and NY-ESO-1. Notably, PMEL, Titin, and CEA induced toxicities in clinical trials when recognized by TCR-engineered T cells [57–59]. While no significant toxicities for WT1 and NY-ESO-1 were reported in multiple clinical trials, both have raised concerns about their innocuity. Indeed, WT1-targeted T cells were shown to target healthy renal cells (which express WT1) [60], and NY-ESO-1 vaccination induced multiple adverse events such as anorexia, hypertension, lung injury, vomiting, abdominal pain, and rash [61–63]. Besides, BamQuery evidenced substantial cell lineage-specific RNA expression of two MAPs: Melan-A in the skin and MAGE-A12 in the brain. Coherent with this, targeting Melan-A resulted in significant skin/eye autoimmunity in clinical trials [64–66], and MAGE-A12 targeting (by mistake) caused severe neurotoxicity and death [67]. For the five remaining MAPs (derived from SLC45A2, MAGE-A4, hTERT, or MAGE-A3), no toxicities were reported in phase I clinical trials when these antigens were accurately targeted [68–73]. Accordingly, BamQuery evidenced homogenously low expression patterns for these MAPs in normal tissues of GTEx (except in the testis, an immunoprivileged tissue).

Altogether, these results demonstrate that BamQuery is crucial to attribute an exhaustive RNA expression to MAPs and suggest that it could help select safe-to-target MAPs.

### Discovery of tumor-specific antigens in diffuse large B cell lymphoma and AML

Given the capacity of BamQuery to prioritize TAs, we wondered whether it could help identify tumor-specific antigens (TSAs) from raw immunopeptidomic data. Using a proteogenomic approach enabling the identification of TSAs [10], we identified 6869 MAPs from 3 published datasets of diffuse large B cell lymphoma samples (DLBCL) [5].

**Fig. 6:**
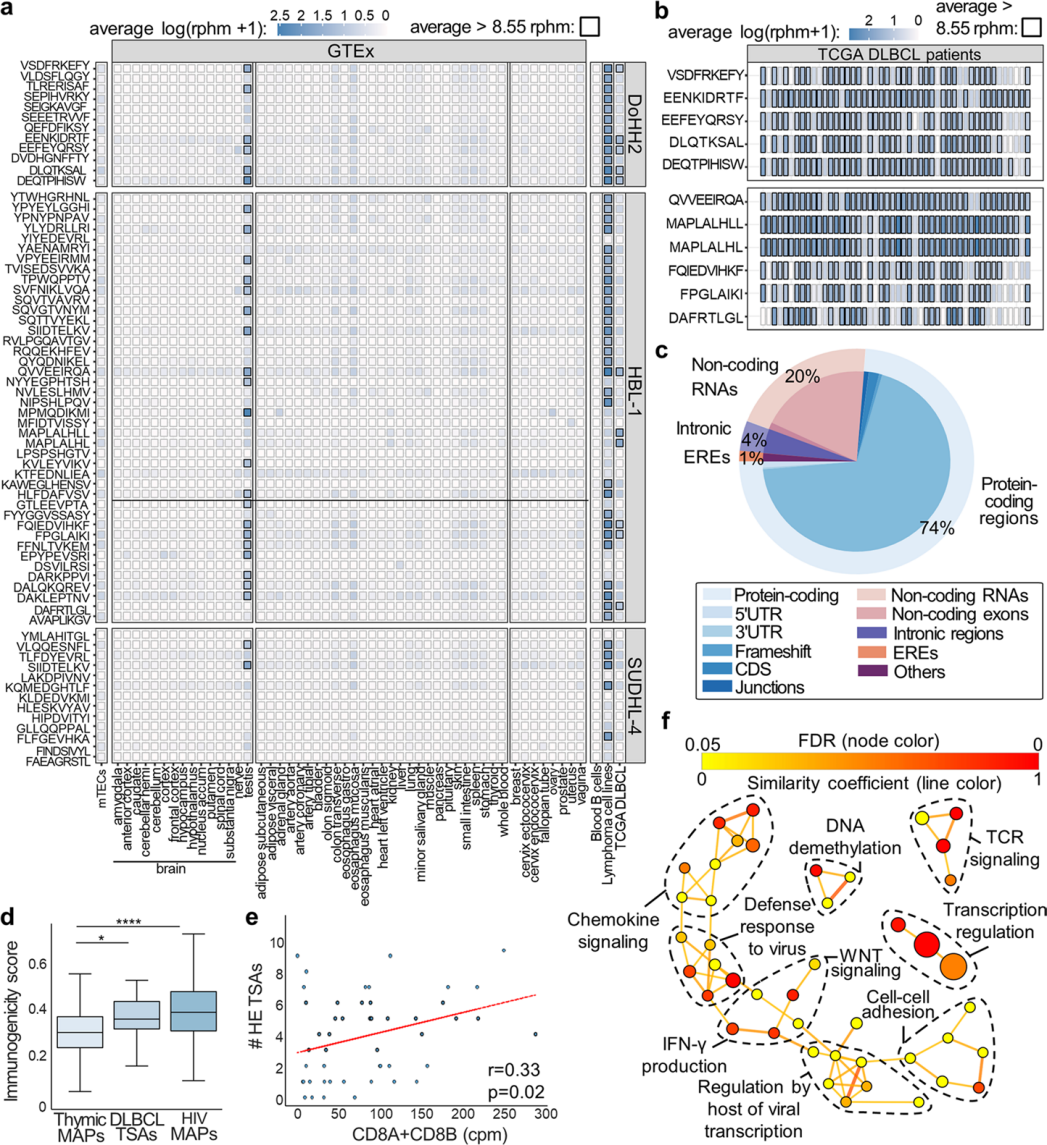
Discrimination of potential immunotherapeutic targets in DLBCL. **a–c** DLBCL MAPs, identified through a TSA-discovery proteogenomic approach, were searched with BamQuery in GTEx tissues (*n* = 12–50 / tissue), mTECs (*n* = 11), sorted blood B cells (*n* = 14), our DLBCL specimens (*n* = 3), and TCGA DLBCL (*n* = 48) bam files in unstranded mode with genome version GRCh38.104 and dbSNP version 155. **a** Heatmap of average RNA expression of 67 TSA candidates in indicated tissues. Boxes in which a peptide has an rphm > 8.55 are highlighted in black. **b** Heatmap of average RNA expression of the highest shared and expressed TSA candidates (11) in cancer samples DLBCL from TCGA (*n* = 48). Boxes in which MAPs expression (rphm) is > 8.55 are highlighted in black. **c** Percentage of the most likely biotype attributed by BamQuery for TSA candidates (*n* = 67). **d** Repitope immunogenic scores calculated for negative control thymic MAPs (*n* = 158), highly expressed DLBCL TSAs (*n* = 18, 25% of TSAs most upregulated by DLBCL TCGA versus normal blood in GTEx and sorted B cells), and positive control HIV MAPs (*n* = 450). Mann–Whitney *U* test was used for comparisons (*p < 0.05, ****p < 0.0001). **e** Pearson’s correlation in TCGA DLBCL patients (*n* = 48) between the count of highly expressed (HE) TSAs expressed by each patient and the expression of cytotoxic T cell markers (CD8A + CD8B, in counts per million (cpm)). The red line is a linear regression. **f** Network analysis of GO term enrichment among genes overexpressed by patients expressing an above-median number of HE-TSAs. Line color reflects the similarity coefficient between connected nodes. Node color reflects the false discovery rate (FDR) of the enrichment. Node size is proportional to gene set size

To discriminate TSAs, we performed sequential searches with BamQuery on different RNA-seq datasets to filter out uninteresting MAPs (Additional file 1: Fig. S10a, Additional file 8). We first quantified the expression of the 6869 MAPs in mTECs. A genomic location was found for 6833 of them, and most of them (~ 86%) were discarded because they were highly expressed in mTECs (≥ 8.55 RPHM). To discriminate MAPs at risk of causing off-target toxicity when targeted, the remaining MAPs (14%) were queried in normal GTEx samples and sorted benign B cells [50, 74]. Through this process, we retained only 5% of the queried MAPs, as they demonstrated minimal expression levels in these normal samples (< 8.55 RPHM). The 67 retained MAPs (of which 62 were unmutated) were flagged as TSAs based on two key features: (i) upregulation by at least fivefold in TCGA DLBCL vs. normal samples, and (ii) evidence of translation based on the presence of ribosomal profiling elongation reads (queried with BamQuery in matched RIBO-seq data [5], Additional file 1: Fig. S10b) (Fig. 6a, Additional file 1: Fig. S10c, Additional file 9). While only one mutated TSA was slightly shared between DLBCL patients (Additional file 1: Fig. S10d), 11 unmutated TSAs were highly shared in the TCGA DLBCL cohort (Fig. 6b), making them promising immunotherapeutic targets.

BamQuery biotype classification showed that most TSAs derived from protein-coding regions of the genome, while ~ 25% of them derived from non-coding RNA (20%), EREs (1%), and intronic (4%) regions (Fig. 6c). Furthermore, based on their high expression in testis, 29 TSAs were flagged as CTAs most of which are known cancer biomarkers [75] (Additional file 10), supporting their relevance as immunotherapeutic targets. Additionally, TSAs upregulated in DLBCL samples compared to normal tissues (GTEx blood and benign B cells) had higher immunogenicity scores predicted by Repitope [76] relative to previously published non-immunogenic controls [77] (Fig. 6d). The expression of these TSAs correlated also with a greater expression of cytotoxic T cell markers (CD8A + CD8B), as well as with TCR signaling and other pro-inflammatory responses in DLBCL patients (Fig. 6e, f, Additional file 11), supporting the biological value of TSAs discovered with BamQuery.

Next, we wondered whether we could use BamQuery to identify mutated TAs in acute myeloid leukemia (AML) since we could only identify non-mutated TAs in this cancer before [9]. From the complete MS identifications of the 19 AML samples reported, we selected all MAPs identified based on the custom cancer-specific proteomes (493) and kept only those expressed by at least 1 AML sample and by none of the normal myelocytic progenitor cells (MPC) controls (70 MAPs selected). Following the discarding of MAPs for which a non-mutated region could code for the peptide sequence, we ended up with only three mutated MAPs (Additional file 1: Fig. S11a). These three MAPs resulted from SNV mutations annotated in dbSNP (rs1177265316, rs1053817098, and rs1324533000) at unique genomic locations. Two of these MAPs were annotated as having a non-coding origin by BamQuery: ALMGNPKVK derived from an intron and ATDDIHHSDRY derived from a non-coding RNA. Interestingly, this low number of mutation-derived MAPs agrees with the low mutation burden that typically characterizes AML [78].

Finally, we wondered whether MAPs for which non-mutated genomic regions were expressed could also derive from other expressed mutated regions in AML. We first filtered MAP-coding regions bearing non-synonymous somatic mutations of the COSMIC database (which is also used by BamQuery to annotate MAPs) [79]. This showed that 127 MAPs could derive from 498 such regions. Next, we filtered these regions to keep only those expressed by at least one AML sample (36 regions left) and not by MPC samples (24 regions left), returning a list of 14 MAPs. The BamQuery annotation of the biotypes that had generated these MAPs showed that 75% of them derived from non-coding regions: EREs, introns, intergenic, and exon–intron junctions (Additional file 1: Fig. S11b, c). Altogether, these results provide evidence that BamQuery can be used to identify mutant TAs from immunopeptidomic data.

### BamQuery: an online tool to facilitate TA prioritization

We implemented an online portal to perform analyses on user-defined lists of MAPs. As we could not enable searches on GTEx (due to the restricted use of these data), we included queries of MAPs in mTECs and DCs [50, 51] (Additional file 3) as a proxy of tumor specificity and immunogenicity. The mTECs promiscuously express an extremely diversified repertoire of genes whose expression is otherwise limited to selected extrathymic epithelial lineages [80, 81]. However, we reported that mTECs share fewer transcriptomic features with hematopoietic cells than epithelial cells [9]. Furthermore, intrathymic central tolerance is established by MAPs displayed by both mTECs and DCs [80–82]. We, therefore, reasoned that the prediction of MAP tolerance should encompass RNA sequences expressed in both mTECs and DCs.

**Fig. 7:**
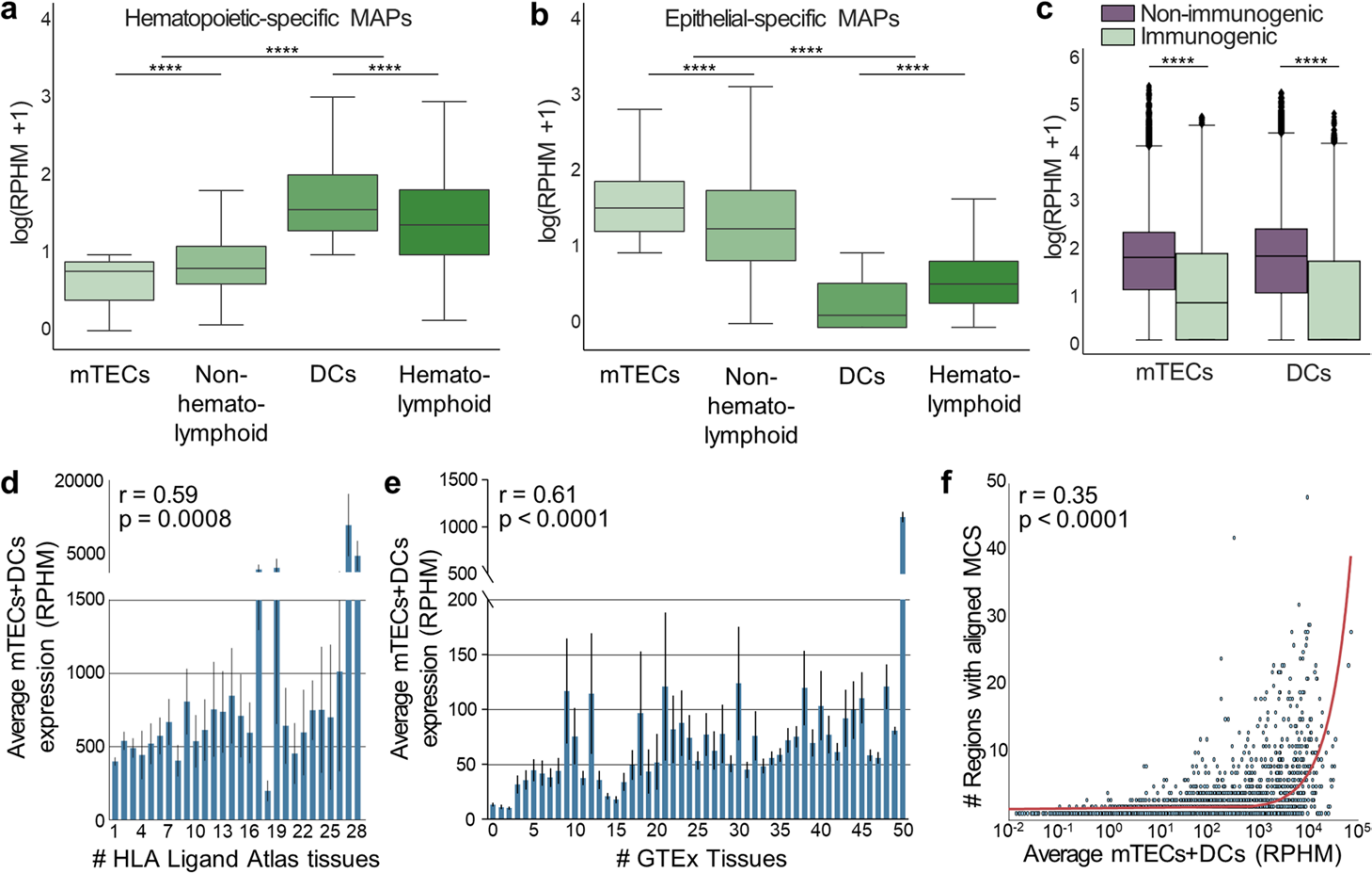
BamQuery: an online tool to facilitate TAs prioritization. **a, b** Average RNA expression of hematopoietic-specific (**a**) and epithelial-specific (**b**) MAPs in mTECs (*n* = 11), non-hematolymphoid GTEx tissues (*n* = 2389), DCs (*n* = 19), and hematolymphoid GTEx tissues (*n* = 196). Wilcoxon rank-sum test two-sided was used for comparisons (****p < 0.0001). **c** Average RNA expression of non-mutated human immunogenic (*n* = 1180) and non-immunogenic (*n* = 4917) MAPs in mTECs (*n* = 11) and DCs (*n* = 19). Mann–Whitney *U* test was used for comparisons (****p < 0.0001). **d, e** Average mTECs + DCs RNA expression of a random selection of MAPs from the HLA Ligand Atlas (*n* = 8621, 10% of the Atlas) as a function of the number of the HLA Ligand Atlas tissues presenting them (**d**) or as a function of the numbe/r of GTEx tissues in which the MAPs are expressed above an average of 8.55 RPHM (**e**). The average expression was correlated (Spearman) with the number of tissues. Error bar, SEM. **f** Spearman’s correlation between the number of expressed genomic locations and the average expression in mTECs and DCs of the same MAPs used in (**d**). The red line is a linear regression (distorted by the log transformation of the *x*-axis)

To validate this choice, we randomly selected 10% of hematopoietic-specific (2429) and 10% of epithelium-specific (3237) MS-validated MAPs from the HLA ligand atlas (Additional file 1: Fig. S12a, b). We queried their expression in mTECs, DCs, GTEx epithelial tissues, and a set of hematopoietic cells (Additional file 3). At the RNA level, DCs and mTECs presented the highest hematopoietic and epithelial MAP expression levels, respectively (Additional file 1: Fig. S12c, d). We refined our analysis by focusing on MAPs differentially expressed in mTECs and DCs; a threshold of 8.55 RPHM was used to differentiate low from high expression. Expression of hematopoietic MAPs followed the following hierarchy: DCs > hematolymphoid tissues > non-hematolymphoid tissues > mTECs (Fig. 7a). The expression hierarchy of epithelial MAPs was strikingly different: mTECs > non-hematolymphoid tissues > hematolymphoid tissues > DCs (Fig. 7b). We conclude that MAPs lowly expressed in mTECs are highly expressed in DCs, and vice versa.

Next, we tested whether MAP expression in mTECs and DCs would predict their immunogenicity. We queried in mTECs and DCs RNA expression of 1180 and 4917 non-mutated human MAPs verified experimentally as immunogenic and non-immunogenic, respectively, and curated in Ogishi et al. [76]. Immunogenic MAPs presented a lower expression than non-immunogenic MAPs in both mTECs and DCs (Fig. 7c). On this dataset, we trained a logistic regression model to classify immunogenic and non-immunogenic MAPs using the RPHM values of mTECs and DCs as features. Measurements of model performance and robustness using the cross-validation method (area under the ROC curve (AUC) =  ~ 0.75, Additional file 1: Fig. S12e) showed that the RPHM values of MAPs in mTECs and DCs are predictors of MAP immunogenicity.

Finally, we evaluated whether MAP expression in mTECs and DCs correlates with their presentation in benign tissues. We randomly selected 10% of MS-validated MAPs from the HLA Ligand Atlas (8,694), then analyzed their expression in mTECs, DCs, and various tissues. MAPs lowly expressed in both mTECs and DCs were less presented (Fig. 7d) and expressed (Fig. 7e) in tissues of the HLA Ligand Atlas and GTEx, respectively. Upon examination of these MAP features, we found that the probability of being highly expressed in mTECs and DCs increased exponentially with the number of potential genomic regions of origin (Fig. 7f). Altogether, these results show that concomitant expression in mTEC and DC expression is a reliable proxy of the presentation/expression in benign tissues and that MAPs having fewer possible regions of origin have a greater probability of being safe-to-target TAs.

The BamQuery public interface is accessible through http://bamquery.iric.ca/ and incorporates the logistic regression predictor model to report the conferred probability that a MAP is immunogenic. BamQuery is also available as a standalone version that can be configured to work with proprietary bam files. We believe that BamQuery will significantly help researchers in their attempts to identify cancer-specific and immunogenic TAs.

## Discussion

Fuelled by studies focused on TAs, the immunopeptidomics field is expanding rapidly [3, 83, 84]. This expansion comes with an impressive diversity of homemade methodological approaches addressing the challenges of characterizing non-canonical and mutated MAPs. Specifically, the fact that ~ 75% of the human genome can be transcribed [85] (and therefore possibly translated) evidenced the necessity of examining the expression of each region able to code for a presumed TA. BamQuery was designed not only to enable such examination but also to enable a uniformization of TA validation approaches across laboratories.

The recent discovery that a significant fraction of the immunopeptidome derives from non-coding regions has brought the contribution of the “dark genome” into the spotlight^2^. Since then, multiple studies have attempted to characterize cryptic MAPs, most often by using MS informed by databases dedicated to the identification of specific classes of ncMAPs (intron-derived, ERE-derived, etc.) [8, 23, 86]. However, these approaches suffer from their dedication as the identified MAPs could also derive from other transcripts absent from these databases. Accordingly, based on evidence showing that greater RNA expression confers a greater probability of MAP generation [7, 13], we implemented a biotype annotation tool in BamQuery and showed that many presumed ncMAPs could be coded with greater probability by regions annotated with different biotypes. Notably, cryptic proteins are translated as efficiently as canonical proteins and generate MAPs fivefold more efficiently per translation event [5]. Hence, BamQuery analyses highlight the need for more in-depth studies to elucidate the precise origin of MAPs, particularly when they are considered therapeutic targets. Nevertheless, we acknowledge that BamQuery’s biotype attributions are based only on RNA expression. Therefore, the biotypes attributed to MAPs coded by several regions with different biotypes should be considered predictions.

Therapies targeting truly tumor-specific antigens can be highly effective [87], while those targeting antigens unsuspectedly expressed by normal cells can be lethal for patients [67]. Notably, BamQuery evidenced a high expression of many TAs, including mutated and ERE MAPs, in normal tissues, resulting from previously unreported coding regions and suggesting that targeting them would be unsafe. Here, we acknowledge that our approach can be considered very cautious. Indeed, by summing the RNA-seq reads of all regions able to code a given TA, BamQuery does not assume that possibly only one region is translated and generates MAPs. Eventually, the availability of RIBO-seq data (which can be analyzed with BamQuery) could help address this question. Meanwhile, in the absence of tools robustly predicting the translational origin of MAPs, the approach reported herein is the most cautious for TA selection. Ideally, we recommend prioritizing TAs with a single possible region of origin (with cancer-specific expression) because other regions cannot code for such TAs in normal tissues.

## Conclusions

Thanks to its exhaustivity, speed, ease of use, and versatility (bulk & single-cell RNA-seq + RIBO-seq, usable with a mouse or human genome on any kind of wild-type or mutated MAPs), BamQuery enables for the first time a uniformization of proteogenomic analyses in MHC-I immunopeptidomics. In particular, we recommend using BamQuery to prioritize TAs having an absent RNA expression in normal tissues (and therefore unable to be presented in these tissues), as these MAPs would be the safest to test in clinical trials.

## Methods

### Availability of data and materials

The Python and R scripts generated during this study are available on GitHub: https://github.com/lemieux-lab/BamQuery (License: MIT [88]) and Zenodo (https://doi.org/10.5281/zenodo.7863816, License: CC-BY-4.0 [89]). BamQuery can be downloaded and installed from http://bamquery.iric.ca/installation.html. Details regarding samples used in this study are listed in Additional file 3. The eight human mTEC samples have been prepared and sequenced in previous studies of our team (GEO:GSE127825 & GEO:GSE127826 [10, 23]). Three additional mTEC samples were published (ArrayExpress:E-MTAB-7383) by Fergurson et al. [31]. Normal RNA-seq samples of healthy tissues were obtained from the GTEx consortium (dbGaP:phs000424.v8.p2). Other datasets include AML (GEO:GSE147524 & PRIDE:PXD018542 [9]), DLBCL (SRA:PRJNA647736 & PRIDE:PXD020620 [5]), single-cell normal lung (BIOPROJECT:PRJEB31843 [40]), single-cell lung cancer (ArrayExpress:E-MTAB-6653 [39]), and DC (GEO:GSE115736 & GEO:GSE76511 [50, 51]) samples. Finally, RNA-seq data for triple-negative breast cancer and high-grade serous ovarian cancer were obtained through the GDC portal of TCGA (https://portal.gdc.cancer.gov/).

### BamQuery

BamQuery is designed to analyze MAPs ranging in length from 8 to 11 amino acids (aa). As peptide input, BamQuery supports three different formats that can be pulled into a single input file.

(A) Peptide mode: only the amino acid sequence of the MAP is provided, hence BamQuery performs a comprehensive search for its RNA-seq expression. All results reported in the present article were obtained with this mode.
(B) MAP-coding sequence (MCS) mode: the amino acid sequence of the MAP is provided, hence BamQuery performs the search for the expression of the given MCS only.
(C) Manual mode: the amino acid sequence of the MAP is provided followed by an MCS, the corresponding location in the genome of the given MCS, and the strand (+ forward or − reverse), whereby BamQuery performs the expression search for the given MCS at the given genomic location and strand.

BamQuery performs five important steps for each peptide queried.

1. Reverse translation of MAPs
  Each input MAP in peptide mode is reverse-translated into all possible MCS. The MCS are compiled into a fastq file. All MCSs provided for peptides in MAP-coding sequence (MCS) mode are included in the same fastq file to facilitate the compilation of their genomic locations.
2. Identification of genomic locations
  MCS are then mapped to the reference genome (user-defined, meaning that several genome versions are supported (GENCODE 26, 33, or 38)) using STAR v2.7.9.a [20] running with default parameters except for –seedSearchStartLmax, –winAnchorMultimapNmax, –outFilterMultimapNmax, –limitOutSJcollapsed, –limitOutSAMoneReadBytes, –alignTranscriptsPerWindowNmax, –seedNoneLociPerWindow, –seedPerWindowNmax, –alignTranscriptsPerReadNmax that were replaced by 20, 10.000, 10.000, 5.000.000, 2.660.000, 1.000, 1.000, 1.000, 20.000, respectively. MCS genomic locations (perfect alignments) are selected from the output STAR file Aligned.out.sam. Perfect alignments are defined as MCS matching exactly the reference genomic sequence or as MCS bearing mismatches annotated as known polymorphisms in the dbSNP database (user-selected dbSNP 149, 151, or 155 releases). Therefore, each alignment included in Aligned.out.sam is examinated to compare the read sequence nucleotide by nucleotide against the reference genomic sequence at that position (assessed using the samtools fetch command within python via the pysam (https://github.com/pysam-developers/pysam) library at the genomic location of the given alignment). If a difference is detected between a nucleotide of the aligned read sequence and the nucleotide of the reference genomic sequence at a given position, the position is queried in the python dictionary containing the SNVs of the dbSNP database selected by the user. If all discrepancies in the current alignment are known (supported by the SNVs in the dbSNP database), the alignment is retained as it is considered perfect; otherwise, the alignment is discarded. To reduce the complexity of tracing perfect STAR alignments, only single-nucleotide variants (SNVs) of dbSNP annotations were considered to define perfect alignments.
3. MAP RNA-seq reads counting
  Next, the expression of each MCS is queried in each BAM file (CRAM files are also supported) using the samtools view [90] command within python via the pysam library (only primary alignment reads (pysam option -F0X100), originally present in fastq files, are queried) at their respective genomic location. BamQuery supports RNA-seq unstrandedness / strandedness libraries (user-defined parameter, default: strandedness). To collect reads in unstranded libraries, the -F0X100 option is used in the pysam view command. In stranded libraries, depending on the sequencing read type (single-end, paired-end), library preparation (forward or backward), and sense of the MCS genomic location (forward or backward), the options in the pysam view command are as follows: -F0X100 & -f0X50 for R1 mate and -F0X100 & -f0XA0 for R2 mate in paired-end, forward library, and reverse genomic location; -F0X100 & -f0X60 for R1 mate and -F0X100 & -f0X90 for R2 mate in paired-end, forward library, and forward genomic location; -F0X110 for R1 mate in single-end, forward library, and forward genomic location; -F0X100 & -f10 for R1 mate in single-end, forward library and reverse genomic location; -F0X100 & -f0X60 for R1 mate and -F0X100 & -f0X90 for R2 mate in paired-end, reverse library, and reverse genomic location; -F0X100 & -f0X50 for R1 mate and -F0X100 & -f0XA0 for R2 mate in paired-end, reverse library, and forward genomic location; -F0X110 for R1 mate in single-end, reverse library, and reverse genomic location; -F0X100 & -f10 for R1 mate in single-end, reverse library, and forward genomic location. The retrieved reads are examined one by one and counted if they exactly span the queried MCS at the genomic location. Therefore, each retrieved read is transformed into a list in Python and its alignment location is transformed into an array containing the location of each amino acid in the read. The indices of the array locations corresponding to the first and last amino acid locations in the MCS at a given genomic location are used to extract from the read list the subsequence that is compared to the MCS. If both the MCS and the subsequence of a retrieved read are the same, the read count for the current MCS increases by one. Finally, the total read count (${tr}_{MAP}$) for a given MAP is computed by summing all RNA-seq reads from all MCS genomic locations.
4. Normalization
  The ${tr}_{MAP}$ count is transformed into “reads per hundred million” values (RPHM) by normalizing them with the total number of primary reads sequenced (corresponding to the total read number present in fastq files) according to the formula: $RPHM= \frac{{tr}_{MAP}}{{R}_{t}}* {10}^{8}$ where ${R}_{t}$ represents the total number of primary RNA-seq reads of the sample. These final values are log-transformed ${log}_{10}\left(RPHM+1\right)$ to allow comparison and averaging between samples, thus removing the bias of large values.
5. Biotype classification
  All genomic locations identified for each MAP are compiled into a bed file and their biotypes are obtained using BEDtools [91] intersect with the following options -a (annotation file), -b (genomic locations), -wao (writes the original annotation, and genomic location entries along with the number of base pairs of overlap between the two features), and the following annotations: RepeatMasker (GRCh38/hg38 assembly, to annotate the EREs) and GENCODE (for all other biotypes, gene set annotations releases v26_88, v33_99, v38_104). The complete list of biotypes annotated by BamQuery based on RepeatMasker and GENCODE can be consulted at http://bamquery.iric.ca/biotype_classification.html.

Given that MAPs may have alignments in regions where several different biotypes overlap (such as protein-coding transcripts overlapping with non-coding RNAs, see the example shown in Additional file 1: Fig. S13), we used the expectation–maximization (EM) statistical model to estimate, for each biotype, the read distribution coefficient. In this model, reads at each genomic location are weighted for each biotype at the given location according to their coefficients, and consequently, the biotype of each MAP is scored according to the percentage of reads corresponding to each biotype (in-frame, introns, ncRNA, ERE, etc.). The EM algorithm iterates between the expectation (E) and maximization (M) step until the parameter set of the last iteration is unchanged, therefore finding the parameter set that maximizes the posterior probability of the observed data, in our case the reads that overlap with one or more biotypes. To train the EM algorithm, we first collected canonical and ncMAPs (Additional file 2) and ran BamQuery on normal and cancer datasets (normal: GTEx and mTECs, cancer: TCGA) to obtain the total reads covering each MAP at each MCS genome location. We then computed the probability of each biotype as follows:

Let $\varnothing =({\varnothing }_{A}, {\varnothing }_{B}, {\varnothing }_{c}\dots )$, be the set of parameters to estimate, where ${\varnothing }_{A}, {\varnothing }_{B}, {\varnothing }_{c}$… are the probabilities that the read belongs to the In_frame (A), non_coding_exon (B), intron (C), etc. biotypes. EM starts with an arbitrary initial estimation of 0.1 for each biotype’s probability. In the E-step, the distribution of the total number of reads for each MAP is computed using the current biotype’s parameters, as follows:

Let $$R_i=total\;reads\;of\;{MAP}_i$$

$$Z\left({\varnothing }_{j, }^{t}{L}_{i}\right)=\frac{{\sum }_{k=1}^{L}\left({r}_{k}*\frac{{\varnothing }_{j}^{t}}{{\sum }_{b=1}^{B}{\varnothing }_{b}^{k}}\right)}{{R}_{i}}$$

where ${\varnothing }_{j}^{t}$ is the current probability for biotype j in ${MAP}_{i}$. ${L}_{i}$ is the MCS genome locations for ${MAP}_{i}$. ${r}_{k}$ is the number of reads overlapping location k and B is the set of biotypes overlapping the location *k*.

In the M-step, the new set of parameters is determined using the current computations, as follows:

$$\varnothing_j^{t+1}=\frac{\sum_{i=1}^{MAPs}\varnothing_j^t}{total\;MAPS}$$

where ${\varnothing }_{j}^{t+1}$ is the new probability for biotype *j* obtained after summing all the probabilities distributions of all MAPs computed in the last E-step and normalizing by the total number of MAPs. The iterative process concludes if the following condition is met for all biotypes: ${\varnothing }_{j}^{t}={\varnothing }_{j}^{t+1}$ and the last set of estimated parameters is used to assign the proportion of reads assigned to each biotype at any genomic location.

Therefore, BamQuery scores for each MAP the biotype as the percentage of reads assigned to each biotype class (in-frame, introns, ncRNA, ERE, etc.). For example, a canonical MAP with alignments in non-canonical regions could be indicated as follows In_frame: 84.09%—Intronic: 15.91%, meaning that ~ 84% of the total reads overlap with a known transcript and that the MAP is within the known protein frame, while ~ 16% of the reads overlap with transcripts in an intronic region.

BamQuery informs the biotype of each MAP in three different settings, as follows:

1. Biotype computed for each MCS genome location: BamQuery reports the percentage contribution of the biotypes overlapping the given location. The percentage of each biotype is calculated as the coefficient of each biotype normalized by the sum of the coefficients of all biotypes in the location, as follows:
  $${\varnothing }_{i,j}^{k}=\frac{{\varnothing }_{i,j}^{k}}{{\sum }_{b=1}^{B}{\varnothing }_{b}^{k}}$$
  where ${\varnothing }_{i,j}^{k}$ is the coefficient assigned to the biotype *j* for the ${MAP}_{i}$ at the location *k*.
2. Biotype computed from all MCS genome locations found in the set of queried samples: the biotype of each MAP is assigned based on the total read count in the sample set. This calculation follows three steps:
  - The total number of reads in each MCS genome location is distributed according to the biotype percentages assigned to the location in the previous step.
  - Normalization of the distributed count of reads by the total number of reads in the entire set of samples.
  - The final biotype of each MAP is obtained by summing all normalized reads distributions across its MCS genomic location.
3. Biotype for each subset of samples (e.g., GTEx, TCGA, mTEC samples): the biotype of each peptide is assigned following the same steps as before but according to the total count of reads in each subset of samples.
4. Best guess biotype: BamQuery also reports the most likely biotype for each MAP (Best Guess) following the rules below:
  - Since a MAP is most likely to be generated from a known canonical protein if the MAP ever appears in-frame of a protein, the best guess assigned is In-frame with the certainty given in the biotype classification.
  - Otherwise, the best guess biotype is assigned according to the biotype with the highest percentage of the biotype ranking. If all biotypes have equal representation, BamQuery reports all of them as the “Best guess”.

Full documentation of supported options, examples of use, and descriptions of all BamQuery reports for biotype classification can be found at http://bamquery.iric.ca/

### K-mer databases

Primary mapped reads were first retrieved from the bam files of each mTEC sample with samtools view [90] (-F260 option), followed by SamToFastq from Picard tools to recover R1 and R2 fastq files (https://broadinstitute.github.io/picard/index.html). Next, R1 reads were reverse complemented using the fastx_reverse_complement function of the FASTX-Toolkit v0.0.14, and fastq files of all mTEC samples were concatenated. Finally, Jellyfish count (v2.2.3, options -m = 27 and -s = 1G) [24] was used to generate the k-mer database from the fastq file, and jellyfish query was used to query the MCS in the database.

### Kallisto quantification

Transcript expression quantifications of mTEC samples were performed with kallisto [15] v0.43.0 quant with default parameters except for –rf-stranded. The expression of each HLA atlas peptide was obtained from the mean TPM expression value of all transcripts associated with the peptide source genes.

### Single-cell RNA-seq analyses

Previously published single-cell RNA-seq data from the healthy and cancerous lungs were downloaded from the NCBI BIOPROJECT (accession number PRJEB31843) and Array Express (accession number E-MTAB-6653), respectively. Reads were aligned on the human reference genome (GRCh38) using STAR version 2.7.9a [20]. Cell population annotations were performed using gene lists from Madissoon et al. [40] and Lambrechts et al. [39] for the healthy and cancerous lung datasets, respectively. For the subsequent profiling of MAP expression with BamQuery, the HCATisStab7509734, and the BT1375 samples were subsampled from the healthy and cancerous lung datasets, respectively. For both genes and MAP expression, read counts were normalized based on the total number of reads detected in each cell (size factor) with the computeSumFactors function of the scran v1.18.7 R package. Normalized read counts were log-transformed with the logNormCounts function of the scuttle package (v1.8.4), and dimensionality reduction was performed with scran (v1.18.7). The differential expression analyses of MAPs between the cell populations of the healthy and cancerous lungs were performed with the FindAllMarkers function of Seurat with the MAST model [45]. Cells of the healthy lungs were also re-clustered based on their MAP expression using the runUMAP and runTSNE functions of the scater package (v1.26.1), and cell lineages and populations previously annotated based on gene expression were represented on the resulting UMAP and TSNE graphs. From the results of our differential expression analysis with MAST, we determined if a MAP has an expression restricted to the hematopoietic lineage, the stromal compartment, or shared by both, based on the cell types by which a given MAP is overexpressed. Finally, MAP expression in the cell populations of the healthy lung was visualized with violin plots using the VlnPlot function of the Seurat package (v.4.1.0) [92].

For heterogeneity of MAP expression in cancer cell analyses, the co-expression of MAPs in the tumor cells of the lung was also assessed. To do so, we selected the MAPs identified as overexpressed in lung cancer cells by the differential expression analysis with MAST and computed Spearman correlations between the expression of each possible pair of MAPs. Cancer cells were isolated from our dataset and were clustered based on gene expression with the same procedure as described above. MAPs differentially expressed between cancer cell clusters were identified with the FindAllMarkers function of Seurat with the MAST model, and MAP expression in the different cancer cell clusters was visualized on the UMAPs and violin plots using the plotUMAP and plotColData functions of scater, respectively.

### Immunogenicity predictions

Immunogenicity predictions of HE-TSAs were performed with Repitope [76]. Feature computation was performed with the predefined MHCI_Human_MinimumFeatureSet variable and updated (July 12, 2019) FeatureDF_MHCI and FragmentLibrary files provided on the Mendeley repository of the package (https://data.mendeley.com/datasets/sydw5xnxpt/1). HIV MAPs (positive control) were obtained from https://www.hiv.lanl.gov/content/immunology/tables/ctl_summary.html.

### Differential gene expression analysis

Transcript expression quantifications were performed on TCGA DLBCL bulk RNA-seq samples with kallisto v0.43.0 with default parameters. Then, with BamQuery, we attributed to each patient a count of highly expressed TSA transcripts (HE-TSA), i.e., the number of TSAs whose expression was above their median RNA expression across all patients having a non-null expression of the given TSAs. Patients having an above-median number of HE-TSAs (*n* = 26) were compared to those below-median (*n* = 22) through a differential gene expression analysis. This analysis was conducted in R3.6.1 as reported previously [93]. In brief, raw read counts were converted to counts per million (cpm), normalized relative to the library size, and lowly expressed genes were filtered out by keeping genes with cpm > 1 in at least 2 samples using edgeR 3.26.8 and limma 3.40.6. This was followed by voom transformations and linear modeling using limma’s lmfit. Finally, moderated t-statistics were computed with eBayes. Genes with *p*-values < 0.05 and − 1 ≥ log_2_(FC) ≥ 1 were considered significantly differentially expressed (386 genes upregulated and 1304 downregulated).

### GO term and enrichment map analyses

Biological-process gene-ontology (GO) term over-representation was performed with DAVID (https://david.ncifcrf.gov) on genes upregulated by DLBCL patients expressing high levels of HE-TSAs. Functional annotations with *p*-value < 0.05 were considered significant. The GO-term list was then imported in Cytoscape v3.7.2 and used to cluster redundant GO terms and visualize the results with EnrichmentMap v3.2.1 and default parameters. The network was visualized using the default “Prefuse Force-Directed Layout” in Cytoscape. Groups of similar GO terms were manually circled.

### Other bioinformatic analyses

Amino acid compositions were assessed with the ProtParam module of Biopython. Read coverage in scRNA-seq data was evaluated with the geneBody_coverage module of RSeQC on the bam file generated by CellRanger. Codon frequencies were obtained from the codon usage database (http://www.kazusa.or.jp/codon/).

To obtain a list of canonical MAPs able to be also coded by ERE regions (dataset used in fig S2b-d), we extracted all genomic regions corresponding to the EREs annotated in repeatmasker. Then, we generated k-mer databases (24/27/30/33 nucleotides) from these regions by using Jellyfish [24] and translated the k-mers into peptide sequences by using a homemade python script. Finally, we queried all the MAPs of the HLA Ligand Atlas in this list of peptides. Those present in this list were retained to perform the analyses shown in fig S2b-d.

To obtain a set of peptides similar to those of Fig. 3a, and not being MAPs, we have extracted from the reference genome the contiguous 27-nucleotide sequence of each region able to code for the peptides of Fig. 3a (two contiguous sequences were extracted for each peptide: one upstream and one downstream). Then we translated these sequences into peptide sequences, removed peptides containing stop signals, and performed netMHCpan predictions of their capacity to bind all known MHC-I alleles (by using a 2% rank threshold). We kept only the peptide sequences predicted as non-MHC binders, yielding a list of 14,810 peptides. This high number is because some EREs mapped to many genomic regions, and therefore one ERE could generate many different upstream and downstream peptide sequences (often varying by a single amino acid from one peptide to another). To gather a dataset comparable in size to the dataset of Fig. 3a, for each original MAP, we made random selections among the downstream and upstream non-binder peptides that were generated and assembled the downstream and upstream peptides into a single dataset. This yielded four lists of peptides: canonical (1478), ncRNA (136), intronic (43), and ERE (185). These peptide numbers are not twofold higher than those of Fig. 3a (1211, 207, 68, and 157, respectively), as could be expected because some MAPs generated neighbors with stop codons (that were discarded from the analysis) and the neighbors of others were all predicted to be MHC binders. This dataset was used to generate the results shown in Additional file 1: Fig. S6a-b.

### Logistic regression model

The cross-validation procedure was used to split the training data set into training and validation subsets using the StratifiedShuffleSplit function of the sklearn python library with 10 numbers of splits and 0.2 for test size. Next, the logistic regression model of the sklearn python library was used to classify immunogenic and non-immunogenic MAPs with the default parameters except for the liblinear solver.

### Construction of MS database for TSA identification

We used RNA-seq data from 3 published datasets of diffuse large B cell lymphoma samples (DLBCL) [5]. Cancer-specific proteomes were built using k-mer profiling as described previously [10]. RNA-Seq reads were chopped into 33-nucleotide k-mers and only those present < 2 in mTECs were kept. Overlapping k-mers were assembled into contigs, which were then three-frame translated and linked using “JJ” as separators*.* This database was concatenated with each sample’s canonical proteome for MAP identification.

### Quantification and statistical analysis

All statistical tests used are mentioned in the respective figure legends. For all statistical tests, *, **, ***, ***, and **** refer to *p* < 0.05, *p* < 0.01, *p* < 0.001, and *p* < 0.0001, respectively, and are reported in the figures. Correlations were assessed with the Pearson or Spearman correlation coefficient, a red line in the correlation plots represents the linear regression. Plots and statistical tests were performed using scipy.stats and seaborn packages of Python v3.6.8. Unless mentioned otherwise, all boxes in box plots show the third (75th) and first quartiles (25th) and the box band shows the median (second quartile) of the distribution; whiskers extend to 1.5 times the interquartile distance from the box. Unless mentioned otherwise, all bar plots show the average with error bars: 95% confidence interval (CI).

### Supplementary Information

**Additional file 1.** Supplemental Figures S1-S13.

**Additional file 2: Supplemental Table 1.** Origin of the MAP datasets used in the present study.

**Additional file 3: Supplemental Table 2.** Origin of the RNA-seq samples used in the present study.

**Additional file 4: Supplemental Table 3.** Enrichment of indicated MAPs in indicated clusters, computed from scRNA-seq data of normal lungs.

**Additional file 5: Supplemental Table 4.** Enrichment of indicated MAPs in indicated clusters, computed from scRNA-seq data of malignant lungs.

**Additional file 6: Supplemental Table 5.** List of TAs identified as co-expressed in lung tumor cells, and their attribution to our original clusters or to the new clusters computed by k-nearest neighbors.

**Additional file 7: Supplemental Table 6.** Origin of the mutated TAs evaluated in the present study.

**Additional file 8: Supplemental Table 7.** Report of the number of MAPs remaining after each filtering step in the TSA identification pipeline based on BamQuery.

**Additional file 9: Supplemental Table 8.** List of DLBCL TSAs identified through a BamQuery-based TSA identification pipeline.

**Additional file 10: Supplemental Table 9.** List of DLBCL CTAs identified through a BamQuery-based TSA identification pipeline.

**Additional file 11: Supplemental Table 10.** List of GO terms enriched in the fraction of TCGA DLBCL patients expressing the highest levels of TSAs (top median) identified with BamQuery.

**Additional file 12.** Review history.

## Data Availability

The Python and R scripts generated during this study are available on GitHub: https://github.com/lemieux-lab/BamQuery (License: MIT [88]) and Zenodo (doi: https://doi.org/10.5281/zenodo.7863816, License: CC-BY-4.0 [89]). BamQuery can be downloaded and installed from https://bamquery.iric.ca/installation. Details regarding samples used in this study are listed in Additional file 3. The eight human mTEC samples have been sequenced in previous studies of our team and deposited into the Gene Expression Omnibus (GEO) database (GEO:GSE127825 & GEO:GSE127826 [10, 23, 94, 95]). Three additional mTEC samples were published (ArrayExpress:E-MTAB-7383) by Fergurson et al. [31, 96]. Normal RNA-seq samples of healthy tissues were obtained from the GTEx consortium (dbGaP:phs000424.v8.p2) [32, 97]. Other datasets include AML (GEO:GSE147524 & PRIDE:PXD018542 [9, 98, 99]), DLBCL (BIOPROJECT:PRJNA647736 & PRIDE:PXD020620 [5, 100, 101]), single-cell normal lung (BIOPROJECT:PRJEB31843 [40, 102]), single-cell lung cancer (ArrayExpress:E-MTAB-6653 [39, 103]), and DC (GEO:GSE115736 & GEO:GSE76511 [50, 51, 104, 105]) samples. Finally, RNA-seq data for triple-negative breast cancer (dbGaP:phs000178 [106, 107]) and high-grade serous ovarian cancer (dbGaP:phs000178.v11.p8 [108, 109]) were obtained through the GDC portal of TCGA (https://portal.gdc.cancer.gov/).
