# Supplementary material for: BamQuery: a proteogenomic tool to explore the immunopeptidome and prioritize actionable tumor antigens
Source: Genome Biol. 2023 Aug 15;24:188. doi: 10.1186/s13059-023-03029-1 (PMC10426134; doi:10.1186/s13059-023-03029-1)
Supplement: Supplementary file 1 — Additional file 1. Supplemental Figures S1-S13. [file 13059_2023_3029_MOESM1_ESM.pdf]

BamQuery: a proteogenomic tool to explore the immunopeptidome and prioritize actionable tumor antigens

Maria Virginia Ruiz Cuevas[1,2], Marie-Pierre Hardy[1], Jean-David Larouche[1,3], Anca Apavaloaei[1,3], Eralda Kina[1,3], Krystel Vincent[1], Patrick Gendron[1], Jean-Philippe Laverdure[1], Chantal Durette[1], Pierre Thibault[1,4,6], Sébastien Lemieux[1,2,6], Claude Perreault[1,3,6,7] and Grégory Ehx[1,5,6,7]

[1] Institute for Research in Immunology and Cancer (IRIC), Université de Montréal, Montreal, Quebec H3C 3J7, Canada.

[2] Department of Biochemistry and Molecular Medicine, Université de Montréal, Montreal, Quebec H3C 3J7, Canada.

[3] Department of Medicine, Université de Montréal, Montreal, Quebec H3C 3J7, Canada.

[4] Department of Chemistry, Université de Montréal, Montreal, QC H3C 3J7, Canada

[5] Laboratory of Hematology, GIGA-I3, University of Liege and CHU of Liege, Liege, Belgium

[6] Senior authors

[7] Lead Contact

***Correspondence**: g.ehx@uliege.be (G.E.)

Supplemental Figures

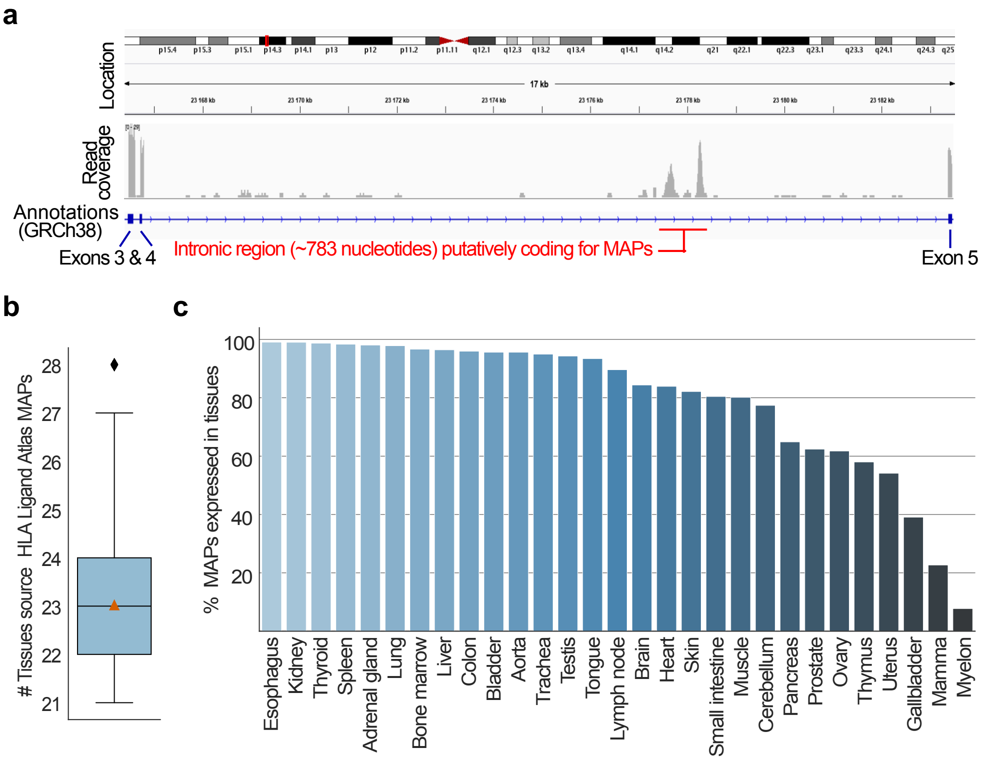

Fig. S1 | Origin canonical MAPs.

a, Genome browser (IGV) illustration for the gene LINC02718 (chr11:23,166,352-23,183,625) in a sample of acute myeloid leukemia (GSM4432540 on GEO) of the heterogeneity of read coverage observed in a typical intronic region (between exon 4 and 5). This heterogeneity would make the usage of genomic annotations irrelevant to quantify the expression of the small region putatively coding for MAPs as most of the annotated intron is not, or lowly, covered by reads (depth of coverage represented in grey).

b, Number of tissues at the origin of the canonical MAPs from the HLA ligand atlas shared in at least 20 tissues (n=1,702). The orange triangle represents the average (23).

c, Percentage of MAPs (n=1,702) presented by the indicated tissues.

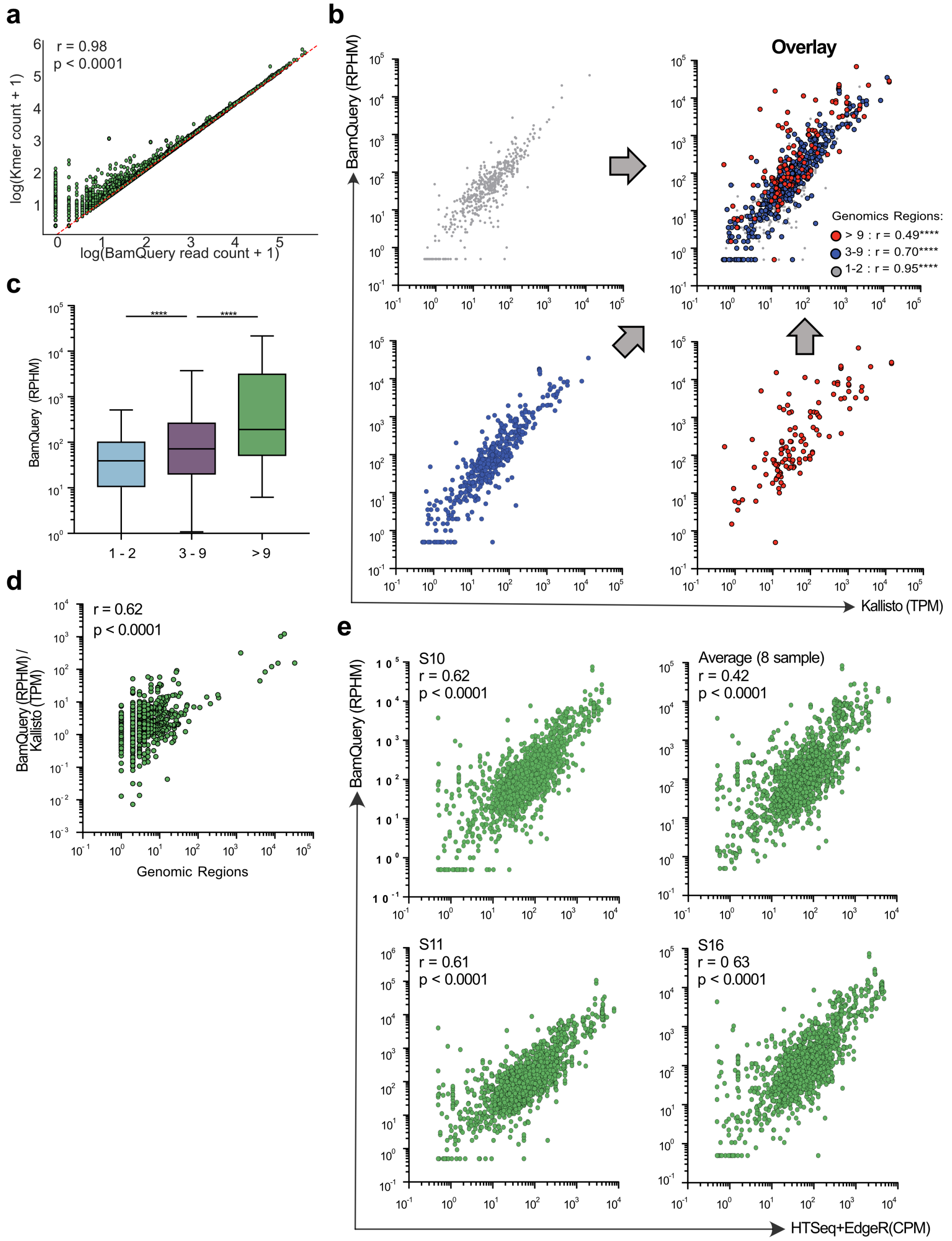

Fig. S2 | BamQuery's quality control.

a,e, Published MAPs reported as canonical (n=1,702) were searched with BamQuery in mTEC bam files in stranded with genome version GRCh38.p13, gene set annotations release v38_104, dbSNP release 151, keeping variants alignments, and allowing higher levels of MCS alignments by STAR.

b-d, Published MAPs reported as canonical and overlapping ERE regions (n=1,126) were searched with BamQuery in mTEC bam files stranded with genome version GRCh38.p13, gene set annotations release v38_104, dbSNP release 151, keeping variants alignments, and allowing higher levels of MCS alignments by STAR.

a, Pearson's correlation between BamQuery-acquired read counts and Jellyfish's K-mer counts for MCS of canonical nonamer MAPs (n=1,211) from the HLA Ligand Atlas (present in at least 20 different tissues) and eight mTEC samples.

b, Pearson's correlation on the average of 8 mTECs samples for MAPs (n=1,126) segregated in three groups as a function of their number of source genomic regions, determined by BamQuery.

c, Box plots of the average expression of MAPs (n=1,126) across the eight mTECs, segregated based on the number of coding regions.

d, Pearson's correlation between the ratio, for each MAP (n=1,126), between the BamQuery and the Kallisto quantification (average of eight mTECs), as a function of the number of coding regions

e, Pearson's correlation between BamQuery's (in RPHM) and HTSeq's (in CPM, generated from the read count of HTSeq with the EdgeR package) quantifications of 1,702 MAPs from the HLA Ligand Atlas in eight mTEC samples. Because HTSeq does not perform direct quantifications of MAPs' RNA expression, the expression of their gene of origin was used as a surrogate. A value of 0.5 was added to each RPHM or CPM value to enable visualization on a logarithmic axis. Correlations for three representative samples and the average of the eight samples are shown.

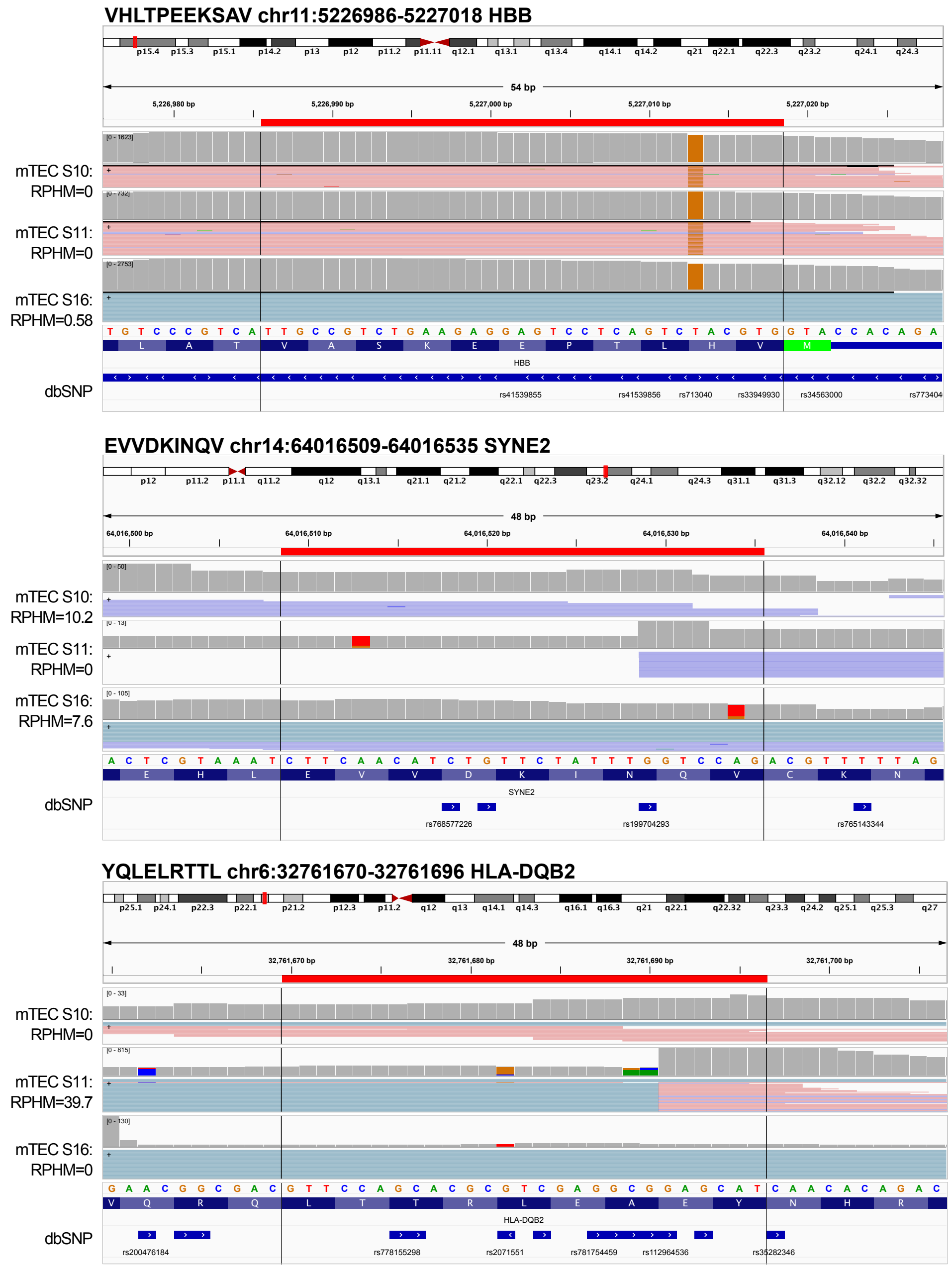

Fig. S3 | Manual validation of peptides with divergent quantifications between BamQuery and Kallisto.

IGV (genome browser) view of the coding regions of three MAPs for which large differences were observed between BamQuery's and Kallisto's quantifications. Each of these MAPs overlapped annotated mutations. The read coverage is shown for each of the three mTEC representative samples (light blue/red) and the positions of dbSNP annotations are shown at the bottom of the plot.

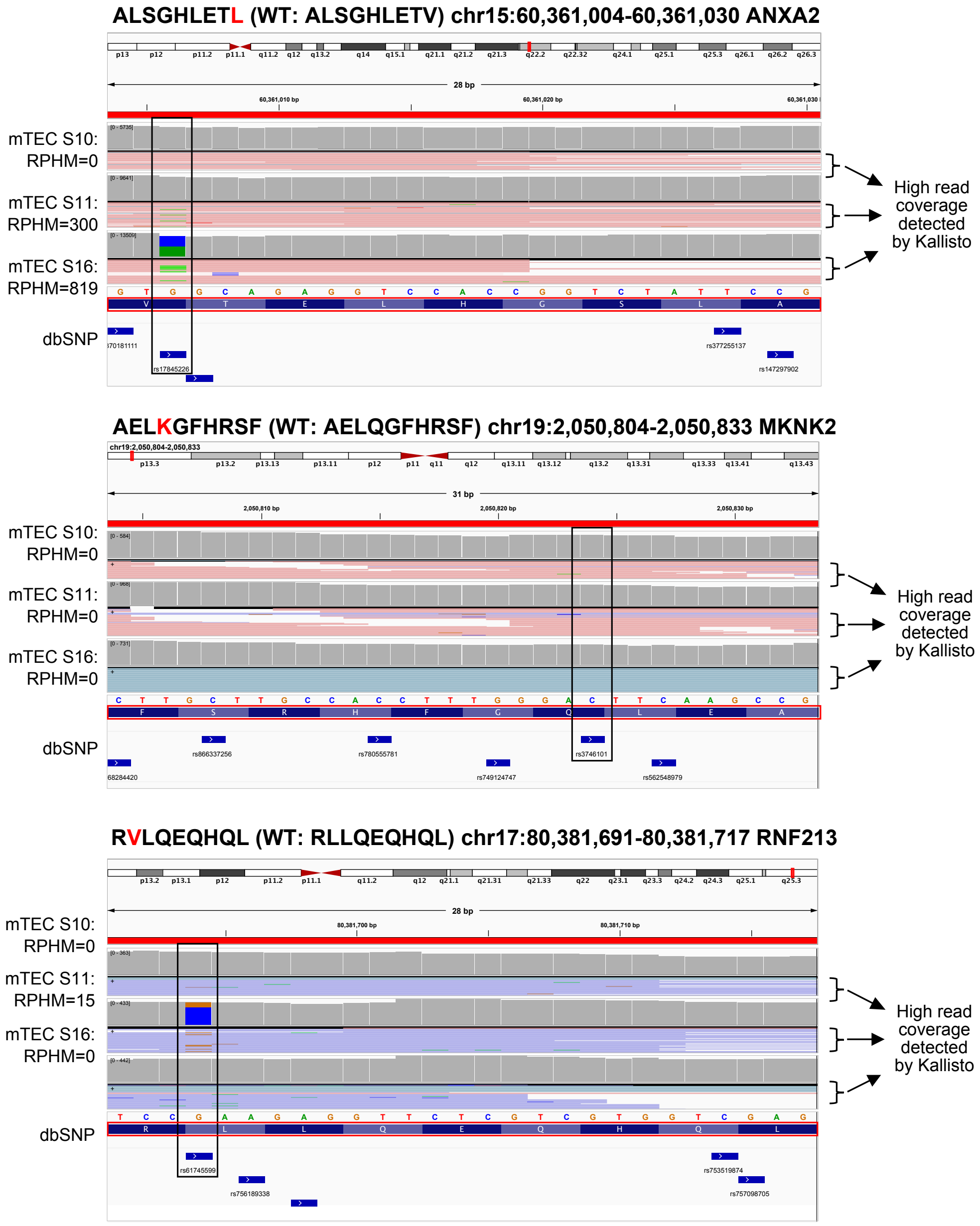

Fig. S4 | Manual validation of miHAs peptides with divergent quantifications between BamQuery and Kallisto.

IGV (genome browser) view of the coding regions of three miHAs for which large differences were observed between BamQuery's and Kallisto's quantifications. Each of these MAPs overlapped annotated mutations. The read coverage is shown for each of the three mTEC representative samples (light blue/red), and the positions of dbSNP annotations are shown at the bottom of the plot.

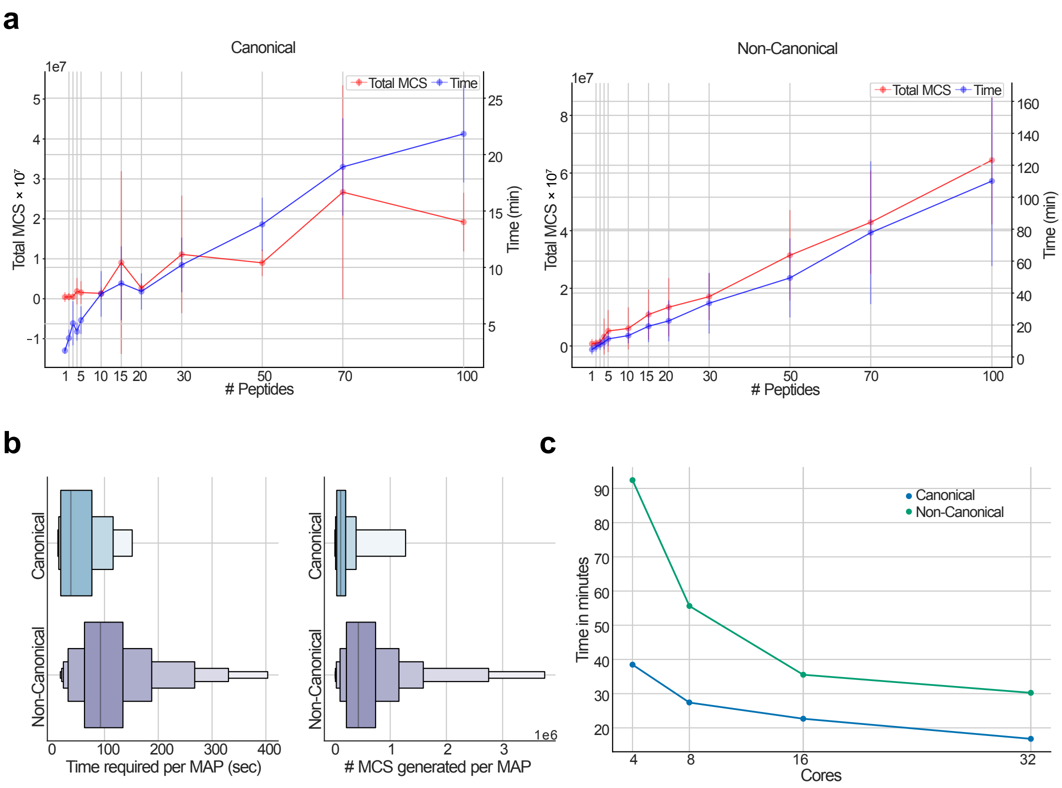

Fig. S5 | BamQuery's time evaluation.

a, Random sampling of canonical MAP peptides (1,702) and non-canonical MAPs (724) to create ten subsets of 1, 2, 3, 4, 5, 10, 15, 20, 30, 50, 70, or 100 peptides. The y-axis shows the total MCS of the peptides (red dots) and the time (blue dots) that BamQuery takes to process. Each dot shows the mean values and standard deviation as error bars.

b, Time required by BamQuery to analyze the expression in eight mTEC of 10 randomly sampled subsets of 1 to 100 canonical and non-canonical MAPs peptides using a minimum capacity of 4 cores (left panel). Number of MCS generated per canonical and non-canonical MAP (right panel).

c, Time required (min) by BamQuery to analyze the expression in eight mTEC of a set of 100 randomly sampled canonical and non-canonical MAPs peptides using 4,8,16, and 32 cores.

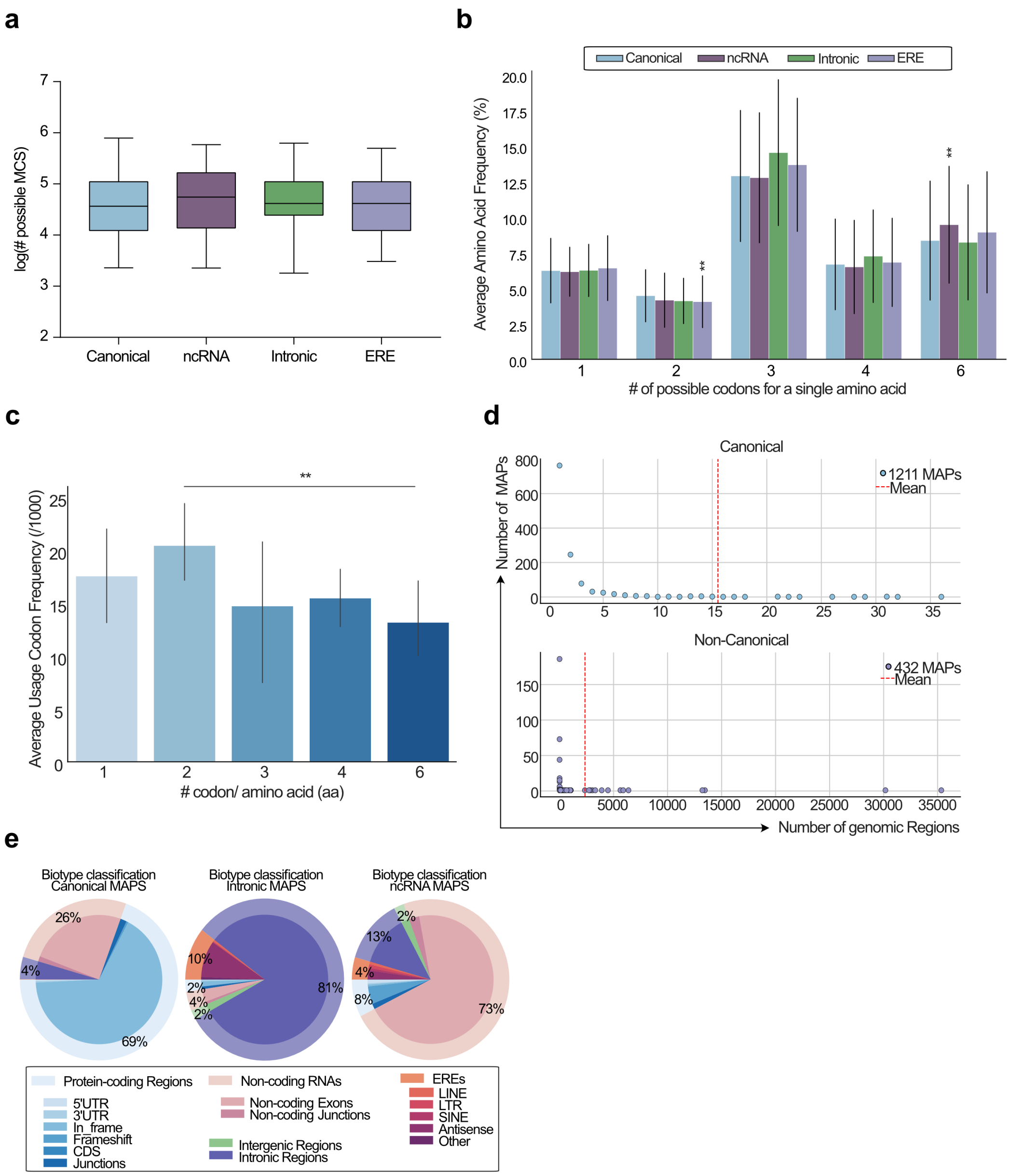

Fig. S6 | Immunopeptidome properties of canonical and noncanonical MAPs.

a, Comparison of the number of possible MCS after reverse translation of a set of nonbinders of MHC molecules (peptides which are not MAPs).

b, Average frequency (%) of amino acids encoded by the indicated number of synonymous codons in indicated groups of peptides which cannot be presented by MHC molecules.

c, Average frequency of codons (among 1000 codons located in human reference protein-coding sequences) encoding each of the 20 amino acids. Codons of amino acids encoded by the same number of synonymous codons were grouped (x-axis).

d, Number of genomic locations from which canonical and non-canonical peptides could originate. The red line represents the mean of 15 and 2,356 genomic locations for canonical and non-canonical peptides, respectively.

e, Percentage of the most likely biotype attributed by BamQuery to canonical, intronic, and ncRNA MAPs.

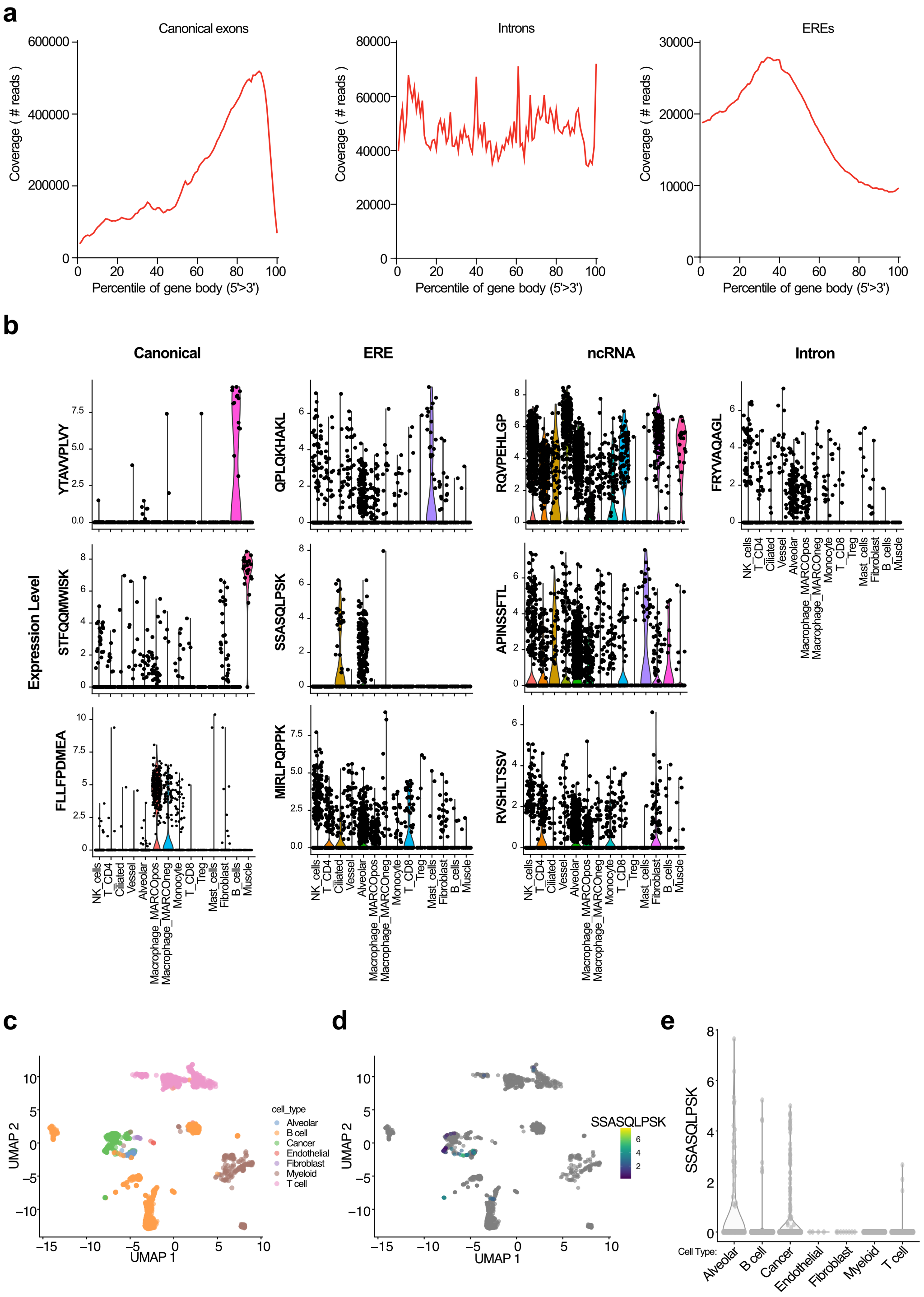

a
Canonical exons
Introns
EREs
Coverage (# reads)
Percentile of gene body (5'>3')
b
Canonical
ERE
ncRNA
Intron
Expression Level
YTAVVPLVY
STFQQMWISK
FLLFPDMEA
QPLQKHAKL
SSASQLPSK
MIRLPQPPK
RQVPEHLGP
APINSSFTL
RVSHLTSSV
FRYVAQAGL
NK_cells
T_CD4
Ciliated
Vessel
Alveolar
Macrophage_MARCOpos
Macrophage_MARCOneg
Monocyte
T_CD8
Treg
Mast_cells
Fibroblast
B_cells
Muscle
c
d
e
UMAP 1
UMAP 2
cell_type
Alveolar
B cell
Cancer
Endothelial
Fibroblast
Myeloid
T cell
SSASQLPSK
Cell Type:

Fig. S7 | BamQuery analysis of normal and cancer lung single-cell datasets.

a, Number of lung scRNA-seq reads covering canonical genes, Intronic regions, and EREs.

b, Expression of canonical, ERE, ncRNA, or intronic MAPs identified as differentially expressed in the normal lung dataset.

c, UMAP showing lung cancer cell clusters based on canonical gene expression.

d, Expression of SSASQLPSK, identified as overexpressed in alveolar and cancer cells is projected on the UMAP lung cancer cells.

e, Expression of SSASQLPSK, identified as overexpressed in alveolar and cancer cells is represented on a violin plot for each cluster of cancer cells.

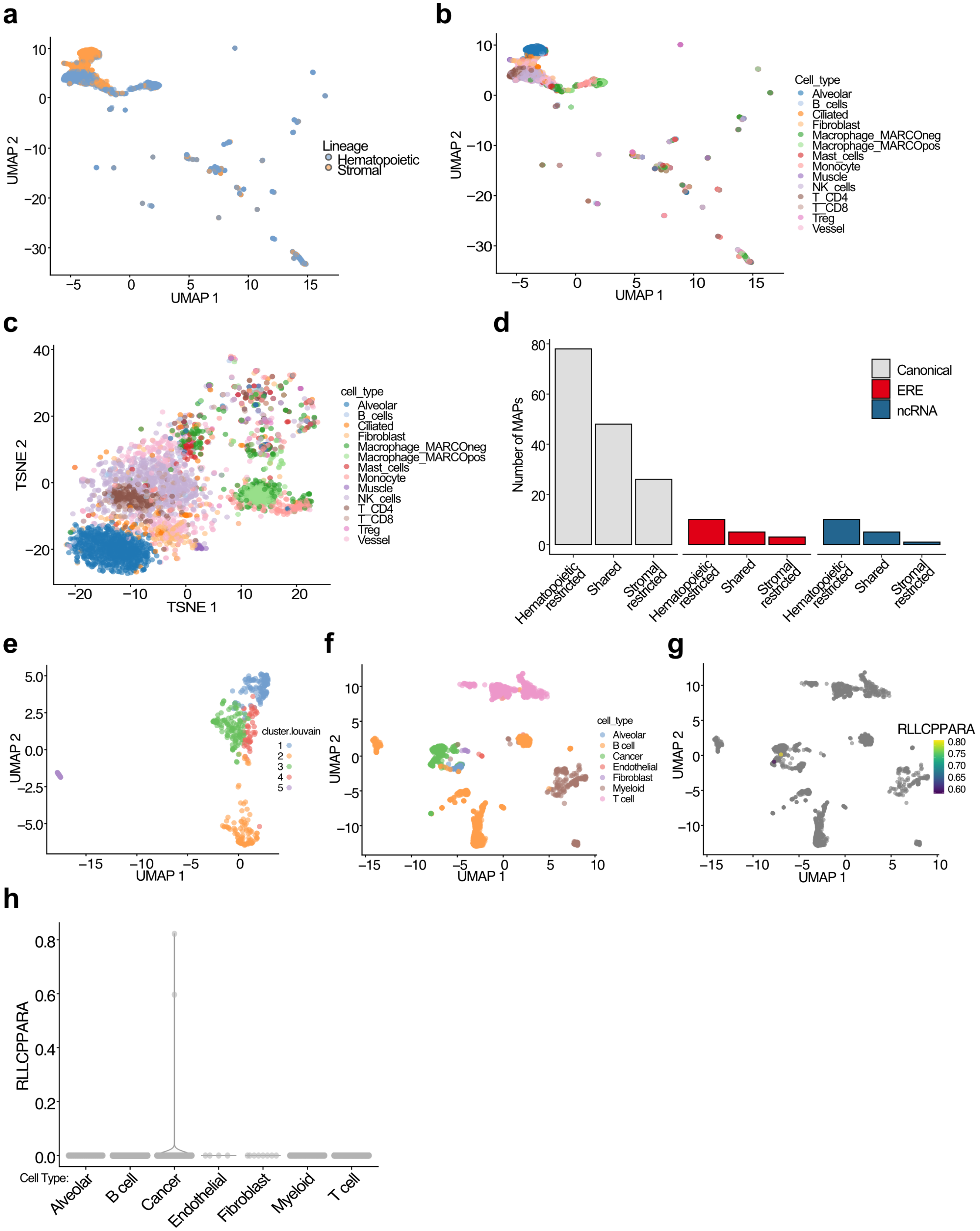
a
UMAP 1
UMAP 2
Lineage
Hematopoietic
Stromal
b
Cell_type
Alveolar
B_cells
Ciliated
Fibroblast
Macrophage_MARCOneg
Macrophage_MARCOpos
Mast_cells
Monocyte
Muscle
NK_cells
T_CD4
T_CD8
Treg
Vessel
c
TSNE 1
TSNE 2
cell_type
d
Number of MAPs
Canonical
ERE
ncRNA
Hematopoietic restricted
Shared
Stromal restricted
e
cluster.louvain
f
cell_type
Alveolar
B cell
Cancer
Endothelial
Fibroblast
Myeloid
T cell
g
RLLCPPARA
h
RLLCPPARA
Cell Type:
Alveolar
B cell
Cancer
Endothelial
Fibroblast
Myeloid
T cell

Fig. S8 | BamQuery analysis of normal and cancer lung single-cell datasets.

a, UMAP depicting the clustering of the hematopoietic and stromal cells from the normal lung based on their MAP expression.

b, UMAP showing the clustering of the cell populations from the normal lung based on their MAP expression.

c, TSNE showing the clustering of the cell populations from the normal lung based on their MAP expression.

d, Number of canonical, ncRNA, or ERE MAPs identified by the differential expression analysis as restricted to the hematopoietic or stromal compartments or shared by cells of both lineages.

e, UMAP showing lung cancer cell clusters based on gene expression.

f, UMAP showing lung cancer cell clusters based on canonical gene expression.

g, Expression of RLLCPPARA, identified as tumor-specific expressed in cancer cells is projected on the UMAP lung cancer cells.

h, Expression of RLLCPPARA, identified as tumor-specific expressed in cancer cells is represented on a violin plot for each cluster of cancer cells.

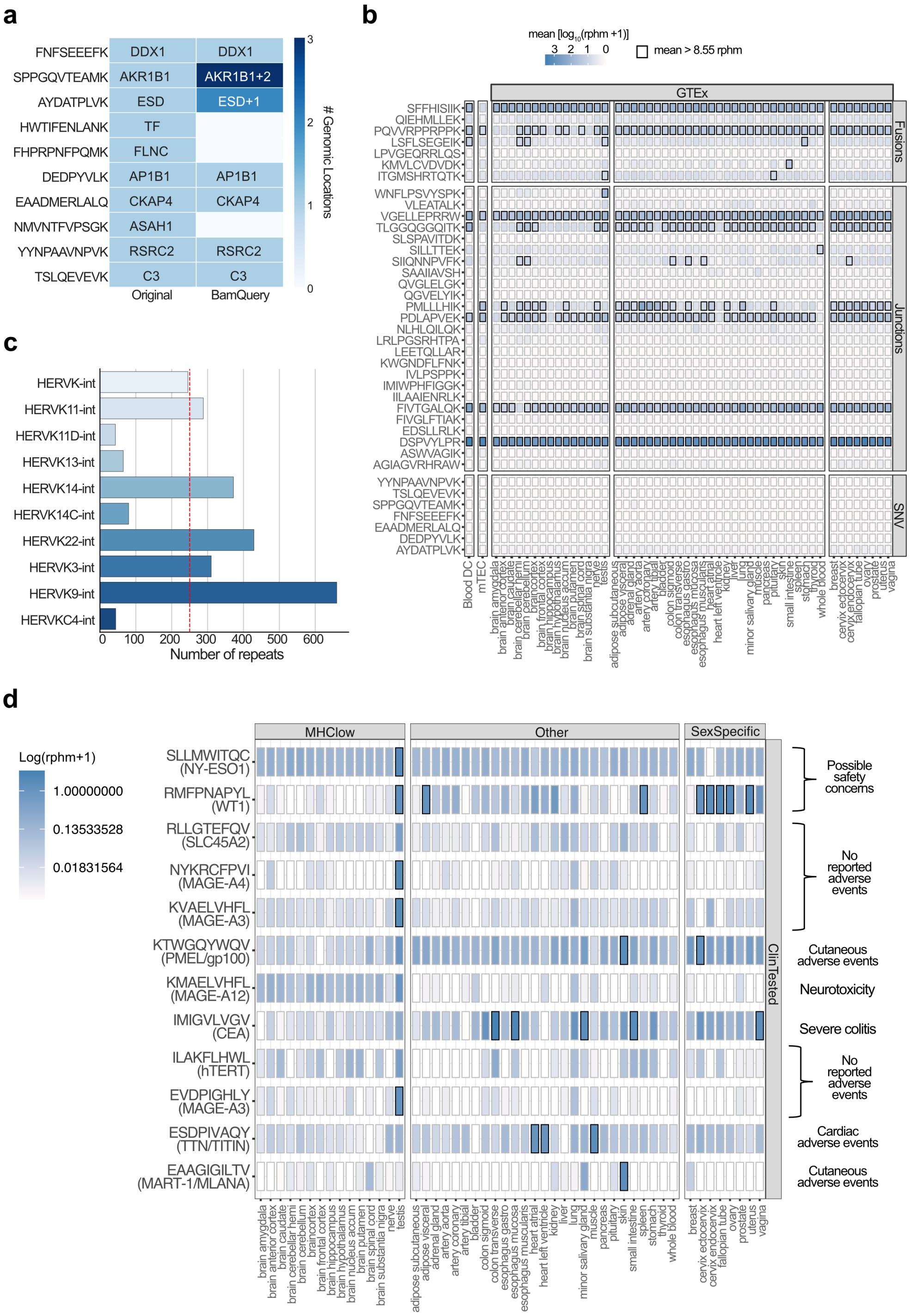

Fig. S9 | BamQuery elucidates safer immunotherapeutic targets.

a, Heatmap of the number of genomic locations at which the expression of the SNVs-derived TAs was assessed by BamQuery vs. by the original study.

b, Heatmap of average RNA expression of published fusions, junctions, and SNVs-derived TAs in indicated tissues. Boxes in which a peptide has an average rphm count >8.55 are highlighted in black.

c, Number of repeats of each HERV-K class collected in the repeat masker database. The red line represents the average of repeats (253).

d, Heatmap of average RNA expression of published TAs suspected to induce autoimmune toxicities. Boxes in which a peptide has an average rphm count >8.55 are highlighted in black.

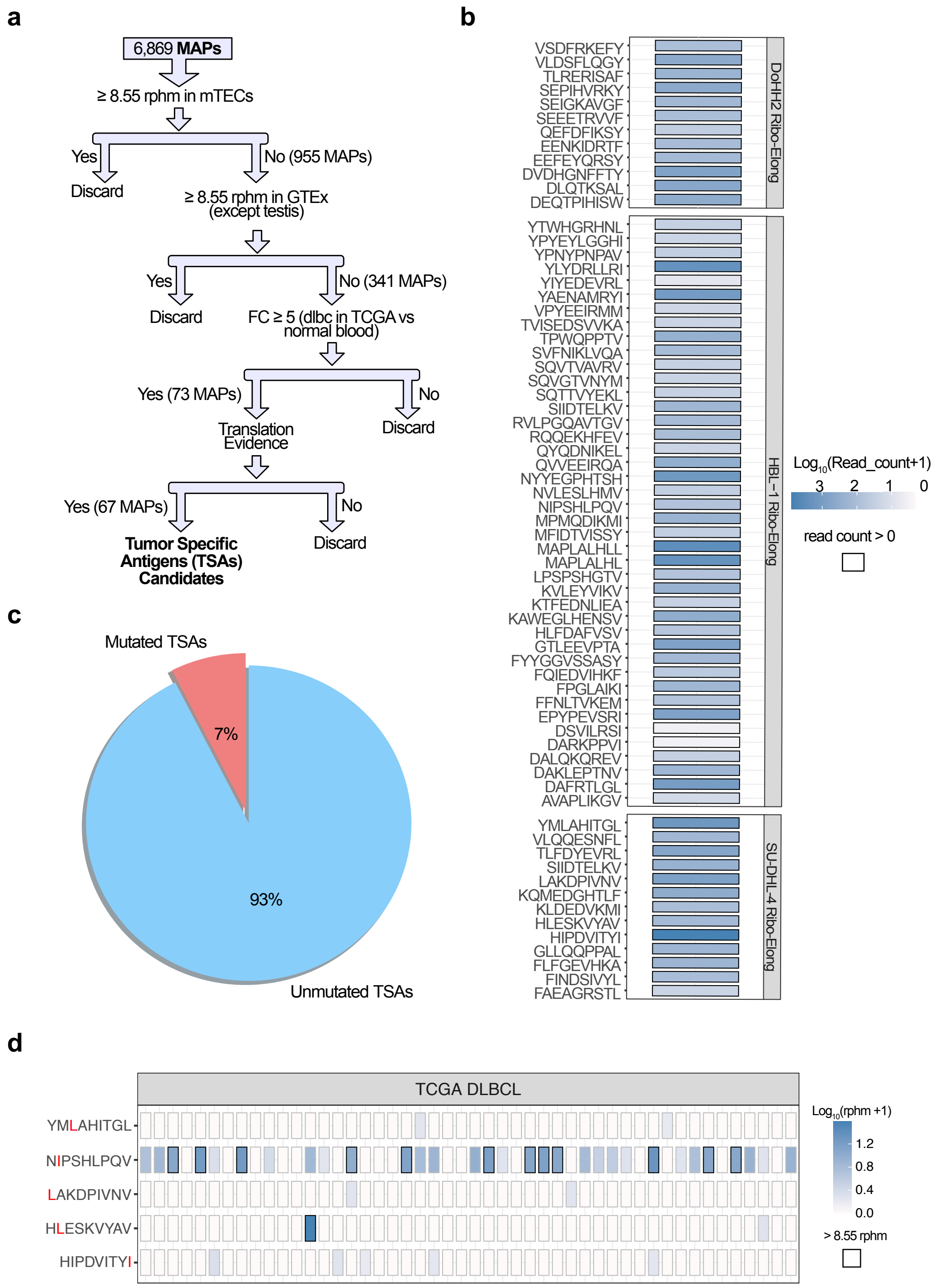

Fig. S10 | Discrimination of potential immunotherapeutic targets in DLBCL.

a, Decision tree to discriminate TSAs from DLBCL.

b, Heatmap of average BamQuery-acquired read count of the 67 TSA candidates in indicated samples. Boxes in which a peptide has an rphm count >8.55 are highlighted in black.

c, Pie chart of the percentage of mutated MAPs among DLBCL TSAs.

d, Heatmap of average RNA expression of mutated TSA candidates (5) in cancer samples DLBCL from TCGA (n=48). Boxes in which MAPs expression (rphm) is >8.55 are highlighted in black.

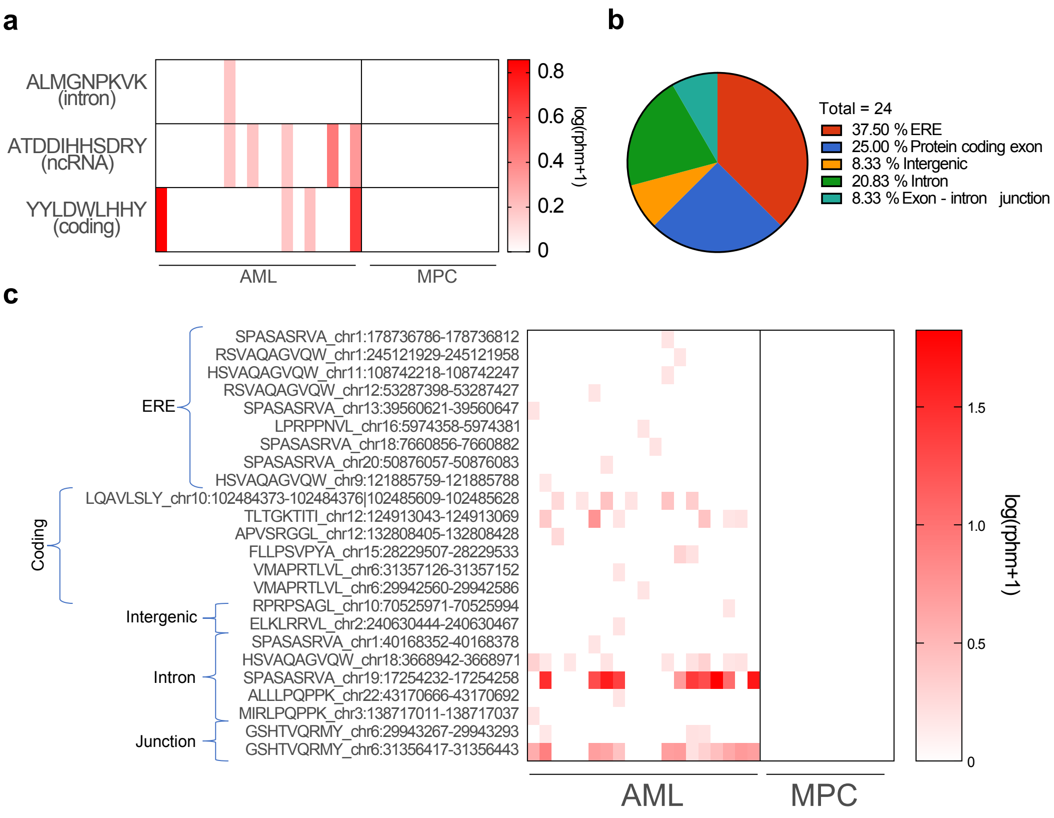

Fig. S11 | Discrimination of potential immunotherapeutic targets in AML.

a, BamQuery's RNA quantification of 3 MAPs unambiguously derived from mutations specific to the 19 AML samples tested (two being shared between samples).

b, Distribution of the biotypes of different regions coding ambiguously for mutated MAPs (MAPs possibly also coded by non-mutated regions).

c, BamQuery's RNA quantification of the different regions coding ambiguously for AML-specific mutated MAPs.

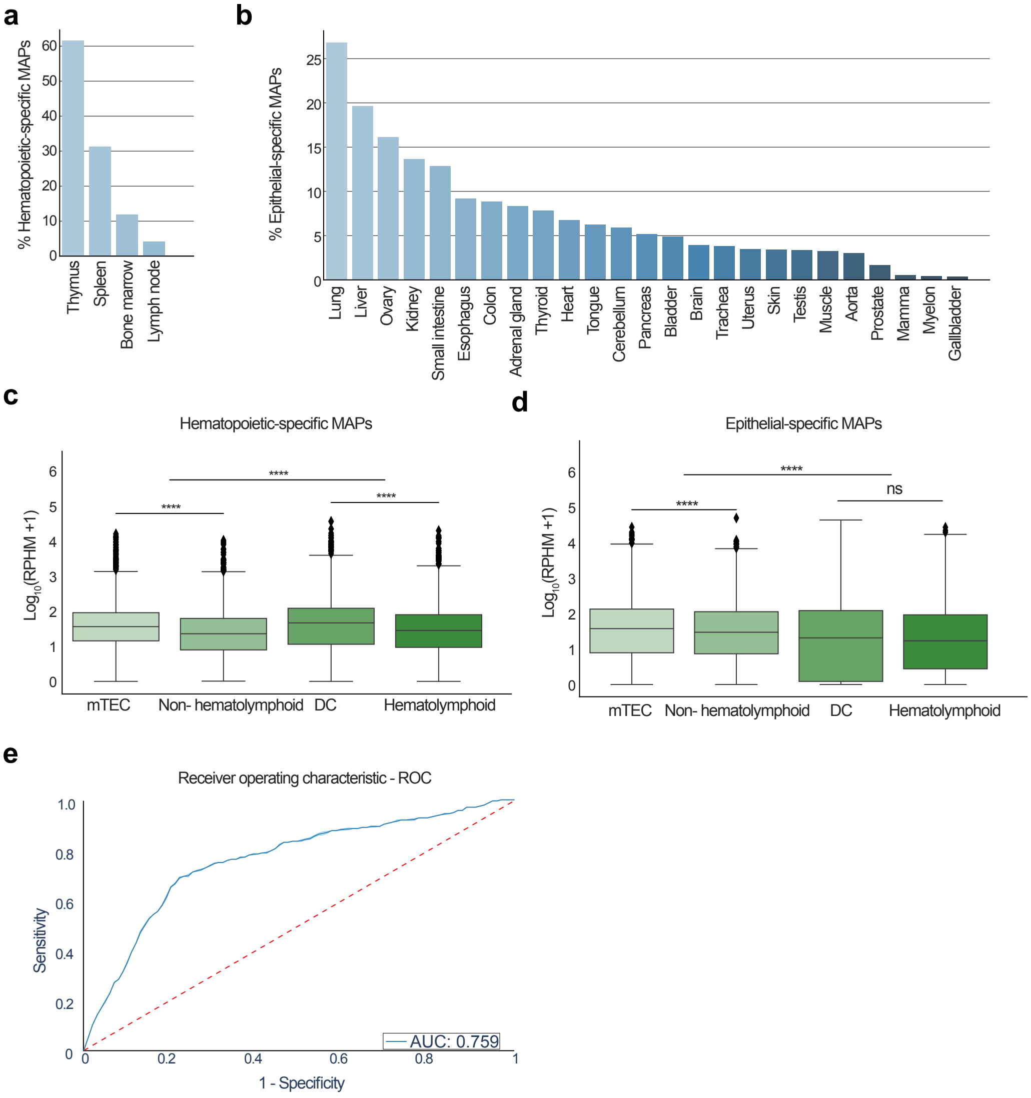

Fig. S12 | mTECs and Blood_DC for TAs prioritization.

a-b, Percentage of hematopoietic-specific MAPs (n=2,429) (**a**) and epithelial-specific (n=3,237) (**d**) presented by the indicated tissues.

c-d, Average RNA expression of hematopoietic-specific (**c**) and epithelial-specific (**d**) MAPs in mTECs (n = 11), non-hematolymphoid GTEx tissues (n = 2,389), DCs (n =19) and hematolymphoid GTEx tissues (n=196). Wilcoxon rank-sum test two-sided was used for comparisons (****p<0.0001).

e, Receiver operating characteristic curve (ROC) for prediction of immunogenicity based on RNA expression (RPHM) in mTEC and DC samples. AUC= ~0.75 with a 95% confidence interval (CI): 0.7588 - 0.7591.

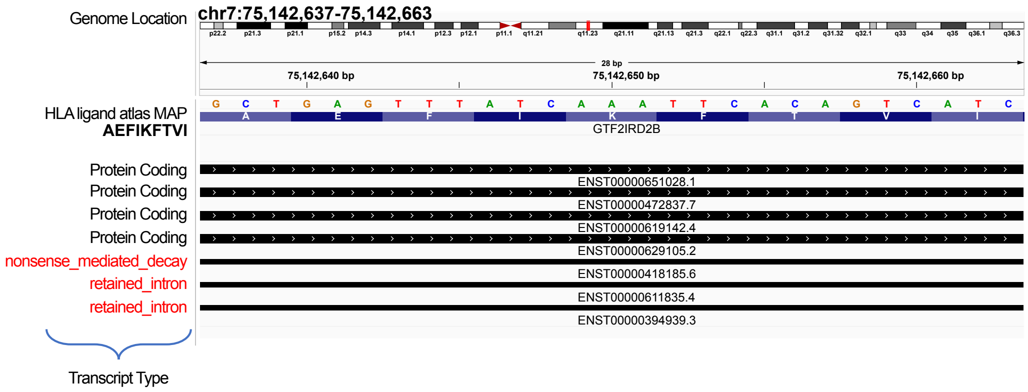

Fig. S13 | Different biotypes overlap at the same genomic location.

The AEFIKFTVI peptide (HLA ligand atlas) at the indicated genomic location overlaps with protein-coding and non-coding RNA transcripts.
