# Supplementary material for: BamQuery: a proteogenomic tool to explore the immunopeptidome and prioritize actionable tumor antigens
Source: Genome Biol. 2023 Aug 15;24:188. doi: 10.1186/s13059-023-03029-1 (PMC10426134; doi:10.1186/s13059-023-03029-1)
Supplement: Supplementary file 12 — Additional file 12. Review history. [file 13059_2023_3029_MOESM12_ESM.docx]

Review History

**First round of review**

**Reviewer 1**

**Were you able to assess all statistics in the manuscript, including the appropriateness of statistical tests used?**

Yes, and I have assessed the statistics in my report.

**Comments to author:**

This manuscript provides some potentially useful tools for evaluating the expression levels of candidate tumor associated antigens that include attempts to map transcripts that encode peptides eluted from cell surface MHC molecules identified by mass spec analysis. There were, however, several points that need to be further addressed.

1. The major issue is that it is not clear that BAMQuery takes into consideration translation, or potential translation, of RNAseq reads. Even though transcript reads might be higher for ncRNA, it doesn't mean that the ncRNA is translated, and therefore processed into a MAP.
2. Another major issue is that prioritization of TAs that are tumor-specific with a single origin appears to be the most important application of this tool, and as such, it seems that the focus of these studies should be on tumor-specific mutations. While identifying highly shared antigens is also important, the danger that the expression in normal tissues might lead to toxicity is a major issue. For example, targeting the melanocyte differentiation antigens MELANA and PMEL with T cells transduced with high affinity TCRs led to high levels of skin, eye and ear toxicity in melanoma patients. In addition, it is difficult or impossible to determine the levels of expression in normal tissue that are acceptable due to the high sensitivity of antigen-reactive T cells.
3. A brief description of how BAMQuery identifies genomic origin (Kmer construction, database formation, etc) in the results shown in Figure 1 should be provided. Although there are previous citations using this method, it would be helpful to the reader to have an explanation in this manuscript.
4. It is not clear how BAMQuery differ from, and why is it objectively better than the other available programs and algorithms designed to detect non-canonical epitopes and peptides. The same dataset should be analyzed using BAMQuery along with additional tools to determine if it is superior to the other methods. The BAMQuery analysis should also be performed on matched tumor and normal tissue samples to determine whether mutant peptides can be identified from introns, EREs, or other non-coding regions of the genome. BAMQuery should have also been used to assess tumor-specific mutations in the lung cancer scRNA-seq data. This would be valuable as it would eliminate the potential risk of targeting epitopes that are expressed at significant levels in normal tissues.
5. The significance of the fact that 32 more peptides using BAMQuery vs Kallisto is unclear, and if this difference is not statistically significant, then it cannot be designated as a superior method. Also, please indicate how Kallisto differs from other transcript abundance quantification tools available that are 'similar', but slower, and indicate whether they be able to detect the 32 peptides that were missing from the analysis with Kallisto.
6. ERE sequences can reside in most genomic locations that are not deleterious to the cell, such as the non-coding regions, but it is not clear how BAMQuery takes this into account. If an ERE was inserted into an intron or another non-coding portion of the genome, what biotype would BAMQuery call the resulting MAP—an ERE or intron? In addition, degraded ERE sequences are scattered throughout the genome that are not active anymore, but do still share sequence homology with their younger, active retroelements. It was unclear if the BAMQuery algorithm references these older, non-active EREs retroelements.
7. The statement was made that 'Canonical MAPs essentially derive from a single genomic location (60%)', but it is not clear why this was referred to as 'essentially' as 60% is just over half of the peptides, as 40% of canonical MAPs can be derived from multiple genomic locations. The percentages of ncMAPs that are derived from a single genomic location should also be noted.
8. It is not clear why there is more homogenous read coverage of intronic and ERE MAPs, as shown in Extended data Fig. 3a when previous results shown in Fig. 3b indicate that the coverage of the 5' and 3' ends of canonical MAPs is nearly identical.
9. In Figure 3f and 3g the results of probing the heterogeneity of intra-tumoral TAs carried out using BAMQuery were presented, but it would be informative to compare these results with those obtained using established pipelines.
10. When referring to the mutant RYLAVAAVF peptide and its wild-type counterpart it not clear why this would be a promising target, as expression of the mutant peptide was not detected whereas the wild type peptide was detected, which of course would not represent a good target.
11. An explanation should be provided as to why BAMQuery able to detect ~66 locations per ERE-derived MAP whereas a previous study was only able to identify 8. A discussion of comparisons between the methods is merited given the wide disparity between the 2 results.
12. It is not clear for DLBCL whether tumor specific antigens also include mutated antigens. It is not clear why RYLAVAAVF why this represented the only mutated peptide analyzed in this manuscript.

There were also several general issues in regarding the definitions of terms used in the manuscript, grammatical errors and other points that should be addressed.

1. It is important to be precise when classifying peptides into their biotypes (tumor antigens (TAs), ncMaps, etc). When introducing 'tumor antigens' (TAs) in the first paragraph of the introduction, a reference was made to '…mutated tumor antigens (TAs)…'. This implies that all tumor antigens discussed in the manuscript will be mutated when in fact most of them are not.
2. The term 'Biotype' should be defined when it is first mentioned on in the introduction on page 4.
3. There are several cases where either 'MAP' or 'MAPs' are the grammar in terms of matching the tense to the sentence are incorrect or the wording is awkward such as in the statement on page 4 '… comparing reliably MAPs RNA expression …'. There are also other instances where the case does not match the noun. For example, '…residues peptide…' should either be changed to '…residue peptide…' or '…peptides of 8-11 residues…'.
4. There are examples of incomplete sentences that need to be altered to contain verbs. These include 2 sentence fragments in the introduction: 'First, the attribution of an exact RNA expression to MAPs' and 'Second, the attribution of a biotype to MAPs.'.
5. Terms such as 'almost perfect' should be avoided, as when reporting correlations of different analysis tools.
6. On Page 10, line 51 'of' should be inserted before >5%.
7. On page 16, line 16, the use of the term 'important' to describe the fraction of canonical MAPs is subjective and should be replaced with another term.
8. On page 12, line 58 the meaning of the phrase '…being retained as being lowly expressed…' is unclear. Does this refer to DLBCL, GTEx, or benign B-cells?
9. The differences between 'Peptide Mode' and 'MCS Mode' should be clarified. Also, as the second and third modes were not evaluated or described in this manuscript (or elsewhere), these should be evaluated in terms of their effects on the BAMQuery algorithm.
10. There are examples where some additional editing may improve the flow of the manuscript. For examples, the 3 aims in the introduction could be combined into 1 paragraph as the second aim is essentially a single sentence. In addition, the second aim, stated as ' Due to the multiplicity of genomic regions able to generate the same MAP, and possibly having different biotypes, a MAP could be
mislabeled for example as ERE-derived while a canonical region could also generate it through
out-of-frame translation.' is confusing and should be reworded.
11. Parts of Figure 2 (panels B, C, and D) showing codon composition do not add substantially to the overall message of the manuscript but could be mentioned briefly and included in the supplemental data.
12. The text in the main figures is too small and can only be read when it is blown up on the screen and so the font size should be increased for these figures.

**Reviewer 2**

**Were you able to assess all statistics in the manuscript, including the appropriateness of statistical tests used?**

Yes, and I have assessed the statistics in my report.

**Comments to author:**

The manuscript describes an approach to infer the expression of MHC-I associated peptides coming from both coding and non-coding genomic regions. It is based on the idea of counting for each peptide the number of occurrences of each RNA sequence that could encode the peptide in RNA-Seq data. This has an advantage compared to standard approaches that consider only protein-coding genes, or predict MAP expression based on the expression of full genomic region(s) where the MAPs come from. The authors then analyzed expression of possibly cancer-specific MAPs in the thymus and/or benign tissues to shed light on whether some of these MAPs may actually be explained be found in such tissues by expanding the search space to non-canonical regions.


- Figure 1B: It still seems that several MAPs have predicted count of 0 (or very low) with BamQuerry, but have > 50 counts in the k-mer database. How can this be? Does it mean that many MAPs would be predicted to have very low expression, when this is actually not the case?
- Figure 1C: It seems that Kallisto actually failed for only 32 out of 1211 MAPs. This is not so bad, considering that Kallisto may discard many of the reads coming from non-coding regions. More generally, I find the comparison between BamQuery and existing tools quite limited. This should be expanded to demonstrate the improvements brought by BamQuery over other approaches.
- In Figure 2, it could be useful to know whether the reported results are specific for MAPs, and whether the same results would be observed for any peptide encoded by canonical/ncRNA/Intronic/ERE elements.
- Figure 3: Although it is nice to see that BamQuery can in principle be applied to scRNA-Seq data, I'm wondering if it practically makes sense to look at each cell separately, and if it would not be better to combine the RNA-Seq data of cells of the same cluster/cell type. How much is the fact that some cells do not express a MAP the result of dropout?
- I think it would be nice to have some time evaluation of the tool. If all possible MCS need to be searched for hundreds of peptides, this could take some time.

Minor comments:

- Page 3, line 34: I do not understand the logic. It is fully expected that the majority of ncMAP are not mutated (mutation represents only a very small fraction of the genome).
- Page 4, line 9: Not sure the degeneracy of the genetic code is the reason why some MAP could map to different regions. To me, it seems more likely that most of the ambiguous MAPs could be due to paralogs/orthologs. This is also supported by Fig. 2f (some MAPs with a limited number of possible MCS are found in many more regions).
- Would be good to define what is meant by 'biotypes'.
- Page 5, line 55: not sure 'benign MAPs" is the best word. A MAP cannot be benign/malignant.
- Figure 1A: I struggled to understand it. Would it not be simpler if only one peptide was used? Also, where does the 73'728 comes from (number of DNA sequences coding for this peptide?) I also suspect that the MCS mode could still lead to multiple mapping to the genome?
- Figure 1B and text: What is JellyFish (not mentioned in the main text). Also what is 'accuracy of 94%' for a correlation, with a Pearson coefficient of 0.97

**Authors Response**

**Point-by-point responses to the reviewers’ comments:**

**Reviewer #1:**

This manuscript provides some potentially useful tools for evaluating the expression levels of candidate tumor associated antigens that include attempts to map transcripts that encode peptides eluted from cell surface MHC molecules identified by mass spec analysis. There were, however, several points that need to be further addressed.

We thank the reviewer for acknowledging the potential of BamQuery. We also wish to thank the reviewer for the time dedicated to the review of our manuscript. We believe that her/his comments have greatly improved our article!

**Major concerns:**

1. The major issue is that it is not clear that BAMQuery takes into consideration translation, or potential translation, of RNAseq reads.  Even though transcript reads might be higher for ncRNA, it doesn't mean that the ncRNA is translated, and therefore processed into a MAP.

**Response to major concern #1:**

We thank the reviewer for this comment. BamQuery counts all reads in a bam file which can code for a peptide of interest given as input to the program. Bam files can be generated from many kinds of sequencing data. The main input type is RNA-seq, for which the most popular method is mRNA capture through polyA selection preliminary to sequencing. When assessing such kind of data with BamQuery, the translation of the tested RNA molecules cannot be taken into account since the bam file does not contain such information. However, bam files can also be generated from ribo-sequencing data, a sequencing following the capture of the RNA molecules actively translated by ribosomes. When assessing that kind of data with BamQuery, the translation is considered since the bam files contain only RNA molecules that are translated. We already used ribo-seq data as the last step in the identification of the best target for diffuse large B-cell lymphoma (DLBCL) immunotherapy, and we identified tumor-specific antigens in 3 cell lines (section 6 of the manuscript). We only retained MAPs that have evidence of translation according to the three ribo-seq elongation data from the three cell lines we analyzed (Fig. S10b). The translation is therefore considered when using adequate data as input of BamQuery.

However, most analyses performed in our article were made on RNA-seq data, and therefore the translation is not assessed directly. Nevertheless, many previously published articles reported a tight correlation between the transcript and the protein abundances (PMID 24870543 30343672 28481885), showing that the RNA abundance can be used as a proxy of the protein abundance (and therefore, translation). To further support this, we have analyzed high-grade serous ovarian cancer data published in PMID 27372738, where large-scale proteomic data on TCGA samples are available. Therefore, we performed correlation analyses between the protein levels reported in this article and the RNA-seq data of the matched samples available on the GDC portal (<https://portal.gdc.cancer.gov/>) of the TCGA group. This showed an excellent correlation between RNA and protein levels (Figure 1 below and new Figure 2a in main text), with only ~15% of transcripts/protein couples not being positively and significantly correlated.

Figure 1. Correlations were made between the proteomic data reported in PMID 27372738 for high-grade serous ovarian cancer patients and matched RNA-seq data. The 3,586 available proteins were used and correlated to their source gene abundance in matched RNA-seq samples obtained on the GDC portal of TCGA. Gene expression abundance was measured with Kallisto. (A) Representative correlation obtained for the TP53BP1 gene across the 115 patients for which matched proteomic and transcriptomic data were available. (B) Distribution of the frequency of genes (n=3,586) and p-values among different categories of correlation values (r), incremented by 0.05 from -0.25 to +0.9.

However, this “only” shows the correlation between protein and transcript abundance, not between transcript and MAP presentation. While multiple previous articles^1-5^ have clearly shown that transcript abundance is a robust predictor of the MAP presentation probability, we also wished to add such evidence to our study. Therefore, we used three DLBCL cell lines, for which matched RNA-seq and mass spectrometry data are available, to compare the expression of transcripts having generated MAPs identified by MS vs. transcripts that have not generated MAPs. We observed that the RNA expression of MAP source genes in DLBCL samples was significantly higher than the expression of the non-MAP source genes (Figure 2 below and new Figure 2b in main text). Furthermore, we also correlated the BamQuery RPHM values between RNA-seq and ribo-seq data for all identified MAPs in the three cell lines investigated. This showed a tight correlation between the two parameters (Figure 3 below and new Figure 2c in main text), showing that transcripts more abundantly expressed are also more abundantly translated. Altogether, these data show that RNA expression, by itself, is a good proxy for predicting the probability of MAP presentation. These data were added to our manuscript on pages 9-10 and associated main figure 2.

Figure 2. Transcription expression level distribution of MAP source and non-source genes in 3 DLBCL cell lines.

Figure 3. Correlation between BamQuery RNA expression and BamQuery Ribo-seq expression (in RPHM) of 6,833 MAPs detected in 3 DLBCL cell lines.

Next, regarding the comment “Even though transcript reads might be higher for ncRNA, it doesn't mean that the ncRNA is translated, and therefore processed into a MAP.”, we understand that the reviewer questions the possibility that an ncRNA could have greater chances of generating the MAP tested than a protein-coding exon also able to generate the same MAP. We apologize if our understanding of this comment is erroneous.

First, we wish to clarify that all MAPs tested in the present manuscript have been identified by mass spectrometry in other articles (the source of the tested MAPs is always indicated in the text). Therefore, their presentation/existence/translation is not questionable. Furthermore, before saying that a MAP unambiguously derives from non-coding regions (such as ncRNA) we verify that the corresponding sequence is found only in non-coding regions (absent in protein-coding exons).

Second, we have designed BamQuery intending to enable exhaustive analyses, not unambiguously attribute a source transcript to MAPs when many of them generate the same peptide. Performing such attribution is a major challenge that would require a fully dedicated article. Nevertheless, with BamQuery, we aim to provide the user the full list of possible transcripts of origin, with an annotation of their nature, also called the biotype (protein coding/introns/ncRNA/etc.), and a machine learning prediction of the most likely transcript of origin, based on the expression of all transcripts able to code for the peptide of interest. It is important to note that our previous research (PMID 33691108) demonstrated that non-canonical proteins are translated as efficiently as canonical proteins and generate MHC-I peptides with 5-fold greater efficiency per translation event. As a result, the predictive power of transcript expression on the likelihood of generating MHC-I peptides also applies to non-canonical MAPs such as introns, non-coding RNAs, EREs, and others. Consequently, attributing an ncRNA origin to a MAP when ncRNA transcripts can code for this MAP and are the most expressed transcripts of origin (even vs. protein-coding transcripts) is scientifically sound. Nevertheless, BamQuery provides many biotype outputs to allow the user to make the best-informed decision when attributing a biotype to the tested MAPs. As explained in our text, two of these outputs are the “best-guess” and the “likelihood ranks”. The first attributes a “protein-coding” biotype to MAPs when at least one such region can code for the peptide (illustrated in Figure 3g, left panel in main text, we have brought this figure back from the supplementary files to better illustrate the different methods used to attribute a biotype), while the other uses only the RNA expression of each of the coding regions to determine the most likely biotype (illustrated in Figure 3g, righ panel in main text). Nevertheless, we have added to the discussion of our manuscript some warning about the biotypes attributed by BamQuery by emphasizing that these attributions are predictions based on RNA expression only (page 23).

In our sense, one of the key applications of BamQuery is assessing the safety of cancer antigens identified by mass spectrometry. To prioritize antigens, we recommend using BamQuery to evaluate their expression in mTECs+DCs and/or all GTEx tissues, with a low expression indicating a higher level of safety. In an ideal scenario, tumor antigens with an RNA expression of zero in all normal tissues should be evaluated in clinical settings, as this suggests they cannot generate peptides. While we agree that “even though transcript reads might be higher for ncRNA, it doesn't mean that the ncRNA is translated, and therefore processed into a MAP”, we prefer considering that BamQuery addresses the following statement: “if transcript reads are absent for that MAP, then it means that the sample considered cannot translate protein products able to generate that MAP”. These considerations have been added to the conclusion of our manuscript (page 24).

2. Another major issue is that prioritization of TAs that are tumor-specific with a single origin appears to be the most important application of this tool, and as such, it seems that the focus of these studies should be on tumor-specific mutations. While identifying highly shared antigens is also important, the danger that the expression in normal tissues might lead to toxicity is a major issue. For example, targeting the melanocyte differentiation antigens MELANA and PMEL with T cells transduced with high affinity TCRs led to high levels of skin, eye and ear toxicity in melanoma patients. In addition, it is difficult or impossible to determine the levels of expression in normal tissue that are acceptable due to the high sensitivity of antigen-reactive T cells.

**Response to major concern #2:**

We agree with the reviewer that validating tumor-derived MAPs is a peculiarly exciting usage of BamQuery. We have altered the title of our article to better reflect this usage. As shown in Fig. 5d, we have evidenced that multiple mutation-derived MAPs, presumably tumor-specific, could be coded by other non-mutated genomic regions. These regions were abundantly expressed in normal cells, suggesting that the coded MAPs could not be as tumor-specific as originally claimed. Since these MAPs are referenced in well-established neoantigen databases and are flagged as possible anti-cancer therapeutic targets, we believe these results demonstrate the necessity of validating neoantigen candidates with BamQuery to prevent off-target toxicities when vaccinating against them. In addition, we added new data regarding mutated MAPs (pages 7-8+Fig 1g; page 14+Fig S8f-h; page 18+Fig S10c-d; page 19+Fig S11), as a response to other questions in the present rebuttal letter.

Nevertheless, the creation of BamQuery was also motivated by recent discoveries showing that non-mutated cryptic genomic regions represent rich sources of actionable tumor-specific antigens (Laumont et al. Sci. Transl. Med. 2018 PMID 30518613; Zhao et al. Cancer Immunol. Res. 2020 PMID 32047025; Ehx et al. Immunity 2021 PMID 33740418). Notably, we have shown in the past that endogenous retroelements (**EREs**), non-coding RNAs, and introns can generate tumor-specific antigens inducing anti-tumor cytotoxic responses upon vaccination (Laumont 2018) or in basal conditions in acute myeloid leukemia patients (Ehx 2021). Other groups reported similar findings in melanoma (Chong et al. Nat. Comm. 2020 PMID 32157095), breast cancer (Bonaventura P et al. Sci. Adv. 2022 PMID 35080970), leukemia (Alcazer et al. Am. J. Hematol. 2022 PMID 35759575) and various other cancers (Smart et al. Nat. Biotech. 2018 PMID 30114007; Lozano-Rabella et al. Clin. Cancer Res. 2023 PMID 36749875; Shah NM et al. Nature Genetics 2023 PMID 36973455).

These recent signs of progress were made possible by using proteogenomics to characterize the tumor-specificity of the MAPs discovered by mass spectrometry. Immunopeptidome analyses by mass spectrometry (**MS**) are characterized by high inter-experiment variability. This variability is well-illustrated by Figure 4E of PMID 34723952 showing that three MS detection of MAPs from the same cell line provide only a 40% overlap between the three samples. Therefore, the absence of detection of a cancer-associated MAP in the immunopeptidome of normal cells does not prove its tumor-specificity. To overcome this limitation, BamQuery examines the RNA expression of the MAP in normal cells since RNA-seq is much more reproducible and sensitive than MS. Performing such analysis is easy for canonical MAPs for which the genomic region is well-defined (whereas BamQuery has shown us that the genomic origin of these MAPs is unclear in ~40% of cases). However, for EREs, which are increasingly investigated as vaccination targets, validating their tumor-specificity is more challenging due to the multiple genomic regions able to code for them, as evidenced by BamQuery. The results in Fig. 5e, f illustrate this challenge well, as BamQuery could detect many more coding regions than previously reported for a set of breast cancer-specific ERE MAPs. BamQuery enables accurate cancer antigens validations by providing a tool evaluating the RNA expression of all regions able to code for a given MAP. Thereby, it could prevent underestimation of MAPs RNA expression, especially in normal tissues where such underestimation could result in toxicities, as highlighted by the reviewer. For all these reasons, we believe that the usage of RNA expression and the extensive discussion of non-mutated cryptic MAPs is adequate for our article. We hope that the reviewer will agree with us.

Regarding the reviewer’s comment about the toxicity issues linked to targeting non-mutated and highly shared MAPs, we agree that targeting this class of peptides represents greater risks of inducing off-target toxicities. However, BamQuery’s analyses in normal tissues can help anticipate such toxicities. We have selected 12 MAPs that were shown/suspected to cause autoimmune toxicities or not in phase I clinical trials of cancer vaccination therapies. As now shown in Fig. S9d (Figure shown hereunder), we have queried their expression in the normal tissues of GTEx. We found that MAPs deriving from WT1, CEA, PMEL, Titin, and NY-ESO-1 presented substantial expression in multiple tissues. Accordingly, PMEL, Titin, and CEA were shown to induce toxicities in clinical trials when recognized by TCR-engineered T cells (on purpose or not, PMID 19451549 21157437 23926201). While no major toxicities were reported for WT1 and NY-ESO-1, concerns have been raised about their innocuity: WT1-targeted T cells were shown to destroy healthy renal cells (which express WT1, PMID 36816758), and NY-ESO-1 vaccination induced multiple adverse events such as anorexia, hypertension, lung injury, vomiting, abdominal pain, and rash (PMID 31980914 35768164 30546433). Interestingly, BamQuery evidenced substantial RNA expression in skin and brain tissues for Melan-A and MAGE-A12, respectively. Accordingly, targeting Melan-A resulted in significant skin/eye autoimmunity in clinical trials (PMID 35865122 11104805 25896248), as mentioned by the reviewer, and MAGE-A12 targeting (by mistake) caused severe neurotoxicity and death (PMID 23377668). For the five remaining MAPs (derived from SLC45A2 (1), MAGE-A4 (1), hTERT (1), or MAGE-A3 (2)), no toxicities were reported in phase I clinical trials (PMID 28630054 25855804 14871958 29908991 14978137 30546947). Accordingly, BamQuery evidenced homogenously low expression patterns for these MAPs in normal tissues of GTEx (except in testis). Importantly, two out of five MAPs for which no toxicities were observed could derive from more than one region (3 regions for KVAELVHFL [MAGE-A3] and 2 regions for NYKRCFPVI [MAGE-A4]). This suggests that MAPs having multiple possible genomic regions of origin should not be automatically ignored and that their exhaustive RNA expression in normal tissues should rather be considered as key selection criteria. These results and considerations have been added to the manuscript (page 17 + figure S9d). Altogether, these data support the relevance of using the RNA expression in normal tissues to predict the probability that a given MAP could induce autoimmunity when targeted.

Finally, we agree that it is challenging to determine the acceptable RNA expression levels in normal tissue due to the high sensitivity of antigen-reactive T cells. However, such levels can still be evaluated since we have shown before (and now in page 9-10 + Fig. 2b,c) that the RNA expression of a transcript reflects its probability of generating MAPs. While a low transcript expression does not guarantee that it won’t generate MAPs (and therefore cannot be recognized by T cells), it still reduces the T-cell recognition probability. We do not pretend to offer “one-size-fits-all” guidelines to discriminate tumor-specific antigens (mutated or not) with BamQuery. However, the data presented in our article (and especially in Fig. 6) demonstrate that promising tumor-specific MAPs (not or very lowly expressed in normal tissues), recognized by CD8+ T-cells, can be identified by using BamQuery and RNA-seq data of normal tissues. Nevertheless, to fully rule out the probability of presentation in normal tissues, one could still use the most parsimonious BamQuery threshold possible to select tumor-specific antigens: null expression in all normal tissues.

3. A brief description of how BAMQuery identifies genomic origin (Kmer construction, database formation, etc) in the results shown in Figure 1 should be provided.  Although there are previous citations using this method, it would be helpful to the reader to have an explanation in this manuscript.

**Response to major concern #3:**

We thank the reviewer for this comment. Here, we want to mention that BamQuery does not use k-mers. We have used k-mer databases to benchmark the accuracy of BamQuery, but such databases are not used by BamQuery (please see also our response to the next comment, where we discuss these k-mer databases). Nevertheless, we have rephrased the first section of the manuscript to introduce the Jellyfish tool used to generate the K-mer database and gave more details in the Methods section as follows:

First section:

“As a control, we used the primary reads contained in the mTEC bam files previously aligned with STAR to generate a database of 27-nucleotide-long k-mers (reads chunked into smaller sequences) using the Jellyfish^6^ tool that counts their occurrences in the primary reads sequences (Methods).”

Methods section:

“**K-mer databases**

Primary mapped reads were first retrieved from the bam files of each mTEC sample with samtools view 61 (-F260 option), followed by SamToFastq from Picard tools to recover R1 and R2 fastq files (https://broadinstitute.github.io/picard/index.html). Next, R1 reads were reverse complemented using the fastx_reverse_complement function of the FASTX-Toolkit v0.0.14, and fastq files of all mTEC samples were concatenated. Finally, Jellyfish count (v2.2.3, options -m = 27 and -s =1G)21 was used to generate the k-mer database from the fastq file, and jellyfish query was used to query the MCS in the database.”

Finally, in both Section 1 and Methods, we provide details on how BamQuery identifies the genomic origin of MAPs:

First section:

“(2) mapping of MCS to the genome using STAR16 to identify those having perfect matches with the reference and attribute them a genomic location. At this step, we also include to the reference genome the mutations from the dbSNP annotations17 to enable the mapping of mutated sequences;”.

Methods section:

***“ 1) Identification of genomic locations.***

MCS are then mapped to the reference genome (user-defined, meaning that several genome versions are supported (GENCODE 26, 33 or 38)) using STAR v2.7.9.a^7^ running with default parameters except for –seedSearchStartLmax, --winAnchorMultimapNmax, --outFilterMultimapNmax, --limitOutSJcollapsed, --limitOutSAMoneReadBytes, --alignTranscriptsPerWindowNmax, --seedNoneLociPerWindow, --seedPerWindowNmax, --alignTranscriptsPerReadNmax that were replaced by 20, 10.000, 10.000, 5.000.000 , 2.660.000, 1.000, 1.000, 1.000, 20.000, respectively. MCS genomic locations (perfect alignments) are selected from the output STAR file Aligned.out.sam. Perfect alignments are defined as MCS matching exactly the reference genomic sequence or as MCS bearing mismatches annotated as known polymorphisms in the dbSNP database (user-selected dbSNP 149, 151, or 155 releases). Therefore, each alignment included in Aligned.out.sam is exanimated to compare the read sequence nucleotide by nucleotide against the reference genomic sequence at that position (assessed using the samtools fetch command within python via the pysam (https://github.com/pysam-developers/pysam) library at the genomic location of the given alignment). If a difference is detected between a nucleotide of the aligned read sequence and the nucleotide of the reference genomic sequence at a given position, the position is queried in the python dictionary containing the SNVs of the dbSNP database selected by the user. If all discrepancies in the current alignment are known (supported by the SNVs in the dbSNP database), the alignment is retained as it is considered perfect; otherwise, the alignment is discarded. To reduce the complexity of tracing perfect STAR alignments, only single nucleotide variants (SNVs) of dbSNP annotations were considered to define perfect alignments.”

We have also improved the clarity of Figure 1a which summarizes how BamQuery works.

4. It is not clear how BAMQuery differ from, and why is it objectively better than the other available programs and algorithms designed to detect non-canonical epitopes and peptides. The same dataset should be analyzed using BAMQuery along with additional tools to determine if it is superior to the other methods. The BAMQuery analysis should also be performed on matched tumor and normal tissue samples to determine whether mutant peptides can be identified from introns, EREs, or other non-coding regions of the genome.  BAMQuery should have also been used to assess tumor-specific mutations in the lung cancer scRNA-seq data. This would be valuable as it would eliminate the potential risk of targeting epitopes that are expressed at significant levels in normal tissues.

**Response to major concern #4:**

We thank the reviewer for these suggestions.

First, BamQuery is objectively better than other available programs designed to detect non-canonical peptides because there are no such (published) programs to our knowledge. However, we could imagine two other methods reaching results similar to BamQuery. We have therefore compared BamQuery to these two other methods (see below). We first summarize the functions of BamQuery to provide a comparison point.

What BamQuery does is (1) detect all genomic regions (and sequences) able to code for a given peptide (it works for peptides deriving from any genomic region: exonic, non-coding, mutated or not); (2) query at each of these regions all RNA-seq reads able to code for the peptide; (3) sum and normalize the RNA expression of each queried peptide; (4) annotate these peptides to attribute their most-likely genomic region of origin and (5) compute an immunogenicity score for the peptides if using our public web portal on mTECs+DCs samples. The main result given by BamQuery is an exhaustive RNA expression of any peptide of interest, in any RNA-seq sample of interest.

The two other methods that we imagined performing analyses “similar” to BamQuery are: (1) queries of all possible peptide-coding sequences (reverse-translated from the peptide) in the fastq files of a given sample and (2) queries of all possible peptide-coding sequences in k-mer databases generated from the RNA-seq reads of the tested sample. While these two methods give accurate counts of the total number of RNA-seq reads able to code for a given peptide (we have notably validated the accuracy of BamQuery vs. k-mer databases in Fig. 1b), they would not provide information about the number, nature, heterogeneity of sequences, and location of genomic regions coding for the peptide. They would also not enable the identification of the most likely genomic region of origin nor provide insights into the immunogenicity of the peptide. Finally, performing single-cell analyses with such methods would raise insurmountable challenges due to the absence of filtering of poor-quality reads (a function performed by the STAR mapper when generating BAM files, on which BamQuery works).

In addition, each of these methods has inherent limitations. The main limitation of the fastq query method is the extremely long time needed for each query (~1 min/sequence queried, so 51 days to query the TVPLNHNTL peptide, which can be coded by 73,728 difference nucleotide sequences in 1 sample vs. 2 min and 57 sec with BamQuery (8 threads) for the same analysis).

The main limitation of the k-mer database query is the disk space required. Indeed, such databases need to be generated from each of the fastq files of the RNA-seq sample considered, and four databases need to be generated for each size of MAP considered (MAPs can be 8-11 residues-long, so k-mer databases of 24-27-30-33 nucleotides length must be generated). Each of these databases occupies a disk space at least twice bigger than the fastq file from which they originate, so from a fastq file of 2Gb, the disk space needed is multiplied by ~9 (18Go). For the GTEx data (which already requires disk space of 5To on our server), it means that at least 45To would be needed. In contrast, BamQuery works on BAM files (which is a format almost automatically generated by all bioinformatic pipelines, including scRNA-seq, and having a size similar to fastq files) or on CRAM files (which are a compressed version of BAM files, requiring ~40% less disk space). One could argue that k-mer databases could be generated from the fastq files each time a query is performed, circumventing the disk space issue. However, this would significantly increase the time required to execute queries as each database takes ~4min to be built (8 threads), resulting in ~16 min needed to query one sample. For GTEx data, this means that 27 days would be necessary to interrogate all 2500 samples (with 8 threads). Please see the speed curves of BamQuery that we have added in Fig. S5a-c for evaluations of our software’s speed. For all these reasons (genomic information, speed, disk space, ease of use), BamQuery is by far superior to the other possible methods. This has been further discussed in the manuscript (page 6).

Here we want to mention that classical transcript-abundance quantification tools, such as Kallisto, do not enable the same type of analysis as those enabled by BamQuery. Therefore, the validation of BamQuery’s accuracy or the benchmark of its speed cannot be performed vs. such tools. What is achieved by these tools is the counting of all reads overlapping a pre-defined region often corresponding to protein-coding transcript annotated in reference databases such as Ensembl (https://ensemblgenomes.org/), UCSC (https://genome.ucsc.edu/) or NCBI (https://www.ncbi.nlm.nih.gov/genome/). These tools are frequently used to evaluate the RNA expression of MAPs by using their gene-of-origin expression as a proxy (PMID 27841757 22438248 35705813 18299400). However, in contrast with BamQuery, these tools do not:

1) Enable the quantification of the RNA expression of MAPs deriving from non-coding regions. This is mainly due to the absence of annotations of the open-reading frames coding for the non-canonical MAPs. Since these tools require such annotations to work, it is, therefore, unable to quantify non-coding transcripts. This is mentioned in the introduction of our manuscript: “Typically, proteogenomic pipelines quantify MAPs RNA expression by estimating their parental transcript expression with conventional tools such as Kallisto ^8^ or HTSeq ^9^. However, such tools cannot be used for ncMAPs which often derive from unannotated transcripts.”

2) Consider all genomic regions able to code for the peptide examined. Therefore, these tools miss some expressed non-exonic regions, also able to code for the peptide. The total RNA expression of the peptides is therefore under-evaluated by these tools. This is notably true for MAPs coded by multiple genomic regions. As now shown in Fig. 1c-f (Figure hereunder), the correlations between Kallisto and BamQuery are generally good (r~0.7). However, they decrease for MAPs deriving from multiple genomic regions, and the difference between BamQuery and Kallisto’s results grows with the number of regions of origin. Here, the BamQuery (RPHM) / Kallisto (TPM) values increase with the number of regions, showing that Kallisto fails to capture an increasing amount of RNA expression with the increase of coding region numbers. Since Kallisto does not assess non-coding regions, while BamQuery does, this increasing difference results from the expression of non-exonic regions. Importantly, these MAPs coded by numerous regions presented a significantly greater expression than those coded by fewer regions, showing that Kallisto’s imprecision is greater for highly expressed peptides (therefore, more likely to be presented widely in normal tissues).

**(A)** Correlation between BamQuery’s (in RPHM) and Kallisto’s (in TPM) quantifications of 1702 MAPs from the HLA Ligand Atlas in 8 mTEC samples. Because Kallisto does not perform direct quantifications of MAPs’ RNA expression, the expression of their gene of origin was used as a surrogate. A value of 0.5 was added to each RPHM or TPM value to enable visualization on a logarithmic axis. Correlations for three representative samples and the average of the eight samples are shown. **(B)** Segregation of MAPs based on their number of coding regions and re-computation of correlations for the average of eight mTEC samples. **(C)** Box plots of the average expression of MAPs in the eight mTECs, segregated based on the number of coding regions. **(D)** Correlation between the ratio, for each MAP, between the BamQuery and the Kallisto quantification (average of eight mTECs), as a function of the number of coding regions.

Because there were few MAPs with high numbers of regions in our original dataset, we built another one from the HLA Ligand Atlas. In brief, we performed a 3-frame translation of the genomic regions overlapping EREs and queried all the HLA Ligand Atlas epitopes in that database. It returned a list of 1,126 MAPs that we analyzed with BamQuery and Kallisto. Identical results were found. The correlation between BamQuery’s and Kallisto’s quantifications decreased for MAPs coded by many regions. The MAPs coded by multiple regions were more expressed than the others, and the ratio BamQuery (RPHM) / Kallisto (TPM) increased with the number of coding regions (Figure 1 below).

Figure 1. Comparison between BamQuery and Kallisto’s quantifications in 8 mTEC samples for 1,126 MAPs from the HLA Ligand Atlas also overlapping genomic regions of EREs (see methods for selection criteria). Because Kallisto does not perform direct quantifications of MAPs’ RNA expression, the expression of their gene of origin was used as a surrogate. A value of 0.5 was added to each RPHM or TPM value to enable visualization on a logarithmic axis. Correlations for three representative samples and the average of the eight samples are shown. (**A**) Correlation on the average of eight mTEC samples for three groups of MAPs segregated as a function of their number of source genomic regions, determined by BamQuery. (**B**) Box plots of the average expression of MAPs in the eight mTEC samples, segregated based on the number of coding regions. **(C)** Correlation between the ratio, for each MAP, between the BamQuery and the Kallisto quantification (average of eight mTEC samples), as a function of the number of coding regions.

3) Quantify the RNA expression of mutated peptides. As explained in our response to comment #5, the transcript quantification tools only count the reads overlapping the gene body. If a non-synonymous mutation is present in that gene body, coding for an alternative version of the protein product, the transcript quantification tools will not be able to assess it. In contrast, BamQuery performs a quantification based on the exact genomic region of each peptide and on the nucleotide sequences able to code for the given peptide. Therefore, it can detect whether the alleles coding for the mutated peptide are expressed or not, yielding an accurate RNA expression value in any sample considered.

In summary, we do not consider that BamQuery and transcript-abundance quantification tools are genuinely comparable in performance/accuracy or speed because they perform radically different types of analyses. Nevertheless, we have performed such comparisons to illustrate the benefits of using BamQuery and because these tools were the most widely used so far to attribute an RNA expression to MAPs by using their assumed source gene RNA expression as a proxy. We have demonstrated hereabove the power of BamQuery for quantifying MAPs coded by many genomic regions. Moreover, we show in response to reviewer 1’s question #5 the power of BamQuery for the analysis of mutated MAPs.

Next, to address the reviewer’s request to perform a BamQuery analysis to identify mutated MAPs deriving from introns or EREs, we used a proteogenomic dataset of 19 AML samples we published recently (Ehx et al. Immunity 2021). We have gathered all MAPs identified by mass spectrometry from these samples and kept only the MAPs flagged as deriving from non-coding regions or mutations. We used BamQuery to quantify the RNA abundance of these 493 MAPs in the parental AML samples and 13 myelocytic progenitor cell (MPC) samples, the normal equivalent of AML blasts. Here we used unmatched samples because we could not find matched cancer and normal samples for which mass spectrometry immunopeptidomic analyses (the only reliable method to identify MAPs, PMID 28985418) were performed and for which matched high-coverage RNA-seq data were available. However, this is not a limitation because we have designed BamQuery to enable such identification even without matched samples, as you will see hereafter.

To identify the most cancer-specific and specifically mutated MAPs, we kept only the MAPs expressed by at least 1 AML sample and none of the MPC samples (70 MAPs). Next, we discarded all MAPs for which a non-mutated region could code for the peptide sequence, leaving 3 MAPs (ALMGNPKVK, ATDDIHHSDRY, and YYLDWLHHY). These 3 MAPs resulted from SNV mutations annotated in dbSNP (rs1177265316, rs1053817098, and rs1324533000, respectively) located at unique genomic locations (chr11:5234120-5234146, chr1:44626363-44626395, and chr16:1242196-1242222, respectively). Two of these MAPs were annotated as having a non-coding origin by BamQuery (based on the RNA expression profile of the AML samples): ALMGNPKVK derived from an intron and ATDDIHHSDRY derived from a non-coding RNA (Panel A of the figure below). Interestingly, this low number of unambiguous mutation-derived MAPs among non-coding MAPs (3 among 493) agrees with the low mutation burden that typically characterizes AML [PMID 23770567]. Nevertheless, we wondered whether MAPs for which non-mutated genomic regions were expressed could also derive from other expressed mutated regions. To address this question, we first filtered MAP-coding regions bearing non-synonymous somatic mutations of the COSMIC database (PMID 30371878) (also used by BamQuery to annotate the analyzed MAPs). This showed that 127 MAPs (out of the 493) could derive from 498 of such regions. Next, we filtered these regions to keep only those expressed by at least one AML sample (36 regions left) and not by MPC samples (24 regions left), returning a list of 14 MAPs. The BamQuery annotation of the biotypes (nature of the genomic region) that had generated these MAPs showed that 75% of them derived from non-coding regions: EREs, introns, intergenic, and exon-intron junctions (Panels B-C of the figure below). Altogether, these results demonstrate that BamQuery can be used to identify mutant peptides deriving from non-canonical genomic regions, from malignant and normal samples. The RNA expression of these MAPs in AML and MPC samples is now reported in Fig. S11, and details about this analysis have been added to our manuscript (pages 19-20).

**(A)** BamQuery’s RNA quantification of 3 MAPs unambiguously derived from mutations specific to the 19 AML samples tested (two being shared between samples). **(B)** Distribution of the biotypes of different regions coding ambiguously for mutated MAPs (MAPs possibly also coded by non-mutated regions). **(C)** BamQuery’s RNA quantification of the different regions coding ambiguously for AML-specific mutated MAPs.

Finally, to respond to the reviewer’s request to identify mutated TAs in the single-cell RNA-seq data of the lung cancer, we queried all 393 mutated neoantigens reported in our Supplementary Table 5 in the single-cell RNA-seq data of the lung cancer. Few (11) of these peptides presented an RNA expression, and among them only one (RLLCPPARA, a melanoma neoantigen: PMID 25837513) presented a tumor-specific expression in the cancer cells (Figure below). These data were added to our manuscript (Fig. S8f-h). This demonstrates the usage of BamQuery to assess the tumor-specificity of mutated neoantigens.

The expression of **RLLCPPARA**, identified as tumor-specific expressed in cancer cells is projected on the UMAP lung cancer cells (Top-panels) or represented on a violin plot for each cluster of cancer cells (Bottom-panel).

5. The significance of the fact that 32 more peptides using BAMQuery vs Kallisto is unclear, and if this difference is not statistically significant, then it cannot be designated as a superior method.  Also, please indicate how Kallisto differs from other transcript abundance quantification tools available that are 'similar', but slower, and indicate whether they be able to detect the 32 peptides that were missing from the analysis with Kallisto.

**Response to major concern #5:**

We thank the reviewer for this comment. While investigating the origin of the missed expression of these 32 peptides by Kallisto, we noticed two sources of bias in our original Kallisto analysis. First, some peptides provided by the HLA Ligand Atlas (the source of these peptides: <https://hla-ligand-atlas.org/>) were not mapped properly to all proteins able to code for them by the atlas team. Therefore, our original Kallisto quantifications were not considering all protein-coding genes able to code for each assessed peptide. Second, the annotations used by the Atlas team for these proteins are from Uniprot, and the annotations used in our Kallisto analyses are from Ensembl. Because these two types of annotations are too different (e.g., multiple Uniprot annotations do not have matched Ensembl annotations, and vice versa), the attribution of source genes to each peptide of the atlas in our original analysis could lack precision. These two sources of bias led us to wonder whether we could improve the analysis shown in previous Fig 1c (and new figure 1c). Therefore, we repeated this analysis by using a different method, independent from the atlas annotations, detailed hereafter: (1) each of the 1,702 peptides was queried in the reference proteome of Ensembl to obtain the complete list of genes able to code for each peptide; (2) the Kallisto transcript-level quantifications were converted to gene-level quantifications by using the Ensembl annotations and the tximport R package; (3) the Kallisto TPM expression of all genes able to code for the same peptide was summed; (4) Kallisto TPM data were compared to BamQuery RPHM data of the same peptides (data shown in new Fig. 1c).

This new analysis showed that Kallisto missed no peptides, so our previous statement that Kallisto missed 32 peptides is no longer valid. This change is due to a better annotation of the source genes of each peptide, as detailed above. Instead, we found good correlations (~0.7 for single samples and 0.51 when averaged all together) between Kallisto and BamQuery results for these peptides (new Fig. 1c-f, Figure 1 below). This result is expected since Kallisto is designed to evaluate the expression of protein-coding transcripts. The similarity demonstrates the accuracy of BamQuery (for a reminder, all MAPs reported in the HLA ligand atlas were identified by mass spectrometry-based on protein-coding gene annotations). However, some peptides presented divergent quantifications between the two methods, with expression values lower with BamQuery than with Kallisto. Upon manual evaluation of these peptides, we found that most of them mapped to genomic regions bearing mutations in the mTECs samples examined. Fig. S3 (Figure 2 below) shows the genomic region of three of these peptides that we used to illustrate the source of these divergences. These examples are detailed hereafter: the VHLTPEEKSAV peptide (as well as its subpeptides HLTPEEKSAV and VHLTPEEKSA) derives from the hypervariable region of the hemoglobin subunit beta gene (HBB). The Kallisto quantification of the HBB gene reported an expression of 98.48 TPM for this peptide in the S10 sample. Kallisto only counts all reads mapping to the gene body of the HBB gene, without considering local allelic variations which can affect the amino acid sequence of its protein product. In contrast, BamQuery successfully captured this variation and returned a count of 0 RPHM in that sample (S10), showing that the sample did not express the alleles enabling the expression of this peptide (a mutation is clearly visible in orange in all examined samples). Similar results were obtained for the EVVDKINQV peptide (which prevented its expression in the S11 sample). The YQLELRTTL peptide is a variant of the YEAELRTTL peptide that could be expressed only by the S11 sample presenting the required alleles in the HLA-DB2 gene. Altogether, these results demonstrate the efficacy of BamQuery at detecting the exact expression of a given peptide. These results were added to the manuscript (pages 7-8).

Figure 1. **(A)** Correlation between BamQuery’s (in RPHM) and Kallisto’s (in TPM) quantifications of 1702 MAPs from the HLA Ligand Atlas in 8 mTEC samples. Because Kallisto does not perform direct quantifications of MAPs’ RNA expression, the expression of their gene of origin was used as a surrogate. A value of 0.5 was added to each RPHM or TPM value to enable visualization on a logarithmic axis. Correlations for three representative samples and the average of the eight samples are shown. **(B)** Segregation of MAPs based on their number of coding regions and re-computation of correlations for the average of eight mTEC samples. **(C)** Box plots of the average expression of MAPs in the 8 mTECs, segregated based on the number of coding regions. **(D)** Correlation between the ratio, for each MAP, between the BamQuery and the Kallisto quantification (average of eight mTECs), as a function of the number of coding regions.

Figure 2. IGV (genome browser) view of the coding regions of three MAPs for which significant differences were observed between BamQuery’s and Kallisto’s quantifications. Each of these MAPs overlapped annotated mutations. The read coverage is shown for each of the three mTEC representative samples (light blue/red), and the positions of dbSNP annotations are shown at the bottom of the plot.

To further demonstrate the superiority of BamQuery at detecting the exact expression of peptides over methods such as Kallisto, we queried the expression of 108 minor histocompatibility antigens (**miHAs**, reported before in PMID 26857467) in the eight mTECs samples tested. These MAPs derive from non-synonymous germline (benign) mutations recognized as immunogenic by allogeneic MHC-matched T cells in hematopoietic cell transplantations. BamQuery could attribute an exact (matching the location reported in the original publication) genomic location to 96% of miHAs. Here, the correlations between BamQuery and Kallisto were very poor, with Kallisto overestimating the expression of many miHAs (Figure 1 below, now Fig 1g-h in manuscript). Three examples of peptides are shown in Fig. S4 (Figure 2 below). For each of these peptides (ALSGHLETL, AELKGFHRSF, RVLQEQHQL), Kallisto detected a high expression (100-1000 TPM), but BamQuery detected an expression of 0 RPHM in the mTEC samples that did not express the adequate allele to code for these peptides. As mentioned above, this overestimation is due to the method used by Kallisto to quantify the expression of genes: by counting reads mapping to the gene body without considering local allelic variations. The number of miHAs having an expression =0 was significantly greater with BamQuery than with Kallisto (Figure 1B below). Together with the impossibility of quantifying unannotated regions with Kallisto (preventing the analysis of non-canonical MAPs, so ~10% of MAPs and currently promising tumor-specific targets), these data further demonstrate the versatility and superiority of BamQuery over other methods in assessing the expression of MAPs (these results were added to the manuscript on page 8).

Figure 1. **(A)** Correlation between BamQuery’s (in RPHM) and Kallisto’s (in TPM) quantifications of 108 minor histocompatibility antigens in eight mTEC samples. Because Kallisto does not perform direct quantifications of MAPs’ RNA expression, the expression of their gene of origin was used as a surrogate. A value of 0.5 was added to each RPHM or TPM value to enable visualization on a logarithmic axis. Correlations for three representative samples and the average of the 8 samples are shown. **(B)** The number of miHAs having an expression =0 according to Kallisto or BamQuery is reported in each of the eight tested mTEC samples (dots).

Figure 2. IGV (genome browser) view of the coding regions of three miHAs for which large differences were observed between BamQuery’s and Kallisto’s quantifications. Each of these MAPs overlapped annotated mutations. The read coverage is shown for each of the three mTEC representative samples (light blue/red), and the positions of dbSNP annotations are shown at the bottom of the plot. The figure shows a high read coverage measured by Kallisto) overlapping the region of the target peptides, suggesting erroneously high expression of the peptide if relying on Kallisto. However, BamQuery counts the exact number of reads able to code for the peptide locally, thereby identifying which samples express the alleles necessary for the expression of the mutated peptide. For the ALSGHLETV peptide in the S11 sample, the allelic frequency of the reads coding for the peptide was too low to be highlighted in color by IGV.

Regarding the reviewer’s question about how Kallisto differs from other quantification tools, we believe that such a detailed explanation is out of the scope of our article. Therefore, we did not add much data about this to our manuscript. Nevertheless, we will respond to the reviewer’s question here. Kallisto is a tool enabling the quantification of transcripts' abundance. The main difference between Kallisto and other tools measuring transcript abundance (such as the very popular HTSeq and Cufflinks) is that Kallisto takes as input the raw reads (fastq files) instead of reads that are already mapped to the genome by an aligner like STAR (bam files). Kallisto performs the alignment + quantification steps altogether, while other tools perform only the quantification. In addition, it works very fast (~15 min/sample with eight cores where HTSeq takes more than 1h). As shown previously, Kallisto provides transcript quantifications similar to other tools but at a much faster speed. It is, therefore, often preferred by bioinformaticians. Nevertheless, as required, we have compared HTSeq and BamQuery, now shown in Fig. S2e (Figure below). Once again, it evidenced relatively good correlations between BamQuery and HTSeq for our reference dataset of 1,702 canonical MAPs. We found that the correlations were less accurate than for Kallisto, probably because HTSeq is a more ancient (and less accurate, PMID 34034652 35337265) tool than Kallisto. Furthermore, HTSeq failed to detect expression for six genes coding for MAPs, while BamQuery found an important expression for these peptides. Unfortunately, we could not find an explanation for this failure because these genes were well present in the gtf file provided to HTSeq and the gtf file (GRCh38.88 from Ensembl) used to build the bam file with STAR was identical to the one used to make the quantification with HTSeq. These results were added on page 7 of our manuscript.

Correlation between BamQuery’s (in RPHM) and HTSeq’s (in CPM, generated from the read count of HTSeq with the EdgeR package) quantifications of 1,702 MAPs from the HLA Ligand Atlas in 8 mTEC samples. Because HTSeq does not perform direct quantifications of MAPs’ RNA expression, the expression of their gene of origin was used as a surrogate. A value of 0.5 was added to each RPHM or CPM value to enable visualization on a logarithmic axis. Correlations for three representative samples and the average of the eight samples are shown.

6. ERE sequences can reside in most genomic locations that are not deleterious to the cell, such as the non-coding regions, but it is not clear how BAMQuery takes this into account.  If an ERE was inserted into an intron or another non-coding portion of the genome, what biotype would BAMQuery call the resulting MAP—an ERE or intron? In addition, degraded ERE sequences are scattered throughout the genome that are not active anymore, but do still share sequence homology with their younger, active retroelements. It was unclear if the BAMQuery  algorithm references these older, non-active EREs retroelements.

**Response to major concern #6:**

We thank the reviewer for this comment. Indeed, during the design process of BamQuery, this was one of the first questions we faced.

To address the possible overlap between different biotypes for a genomic location, as in the case of EREs located in an intron, we wanted to avoid introducing arbitrary rules to determine which biotype to report. Therefore, we implemented and trained the machine learning Expectation Maximization (EM) algorithm on expression data of canonical MAPs, ncMAPs (intron, EREs, ncRNAs), and mutated MAPs in normal (GTEx and mTECs), and cancer (TCGA) samples. This allowed us to estimate a coefficient for each biotype (in-frame, introns, ncRNAs, EREs, etc.) that represents the probability that the biotype is at the origin of the MAPs, thus facilitating the distribution of reads underlying the regions to each biotype. BamQuery then provides each MAP with the percentage of reads assigned to each biotype class based on the coefficient, meaning that the final biotype assignment for the peptide is based on the relative abundance of each biotype in the samples studied.

In the particular case where a MAP, e.g., **AEAGGSPEV**, is in a genomic location where Ensembl and Repeat Masker annotations overlap (Figure 1 below), BamQuery first reports that the MAP location overlaps with four intronic regions and a ERE sequence (AluSx) of the SINE class (Figure 2 below).

Figure 1. IGV (genome browser) view of the **AEAGGSPEV** peptide located at genomic location chr11:117,277,106-117,277,132. At the same location, the peptide overlaps with the introns of four Ensembl transcripts and a RepeatMasker repeat sequence.

Figure 2. BamQuery output *1_Genomic_and_ERE_Annotations_Full.csv*: For a given peptide carrying an MCS mapped to a given genomic location, BamQuery reports all overlapping biotypes in the Ensembl and Repeat Masker annotations.

In addition to the above output, BamQuery reports two consensus biotypes considering all genomic locations of the peptide.

The first consensus biotype is generated by aggregating the reads assigned to each biotype and distributing them according to their frequencies at each genomic location. The aggregated reads per biotype are then weighted by the total reads of all samples investigated. In cases where the In_frame biotype is absent, the "Best guess" biotype is chosen as the biotype with the highest percentage representation. When BamQuery detects that all biotypes have equal representation, it will report all of them as the "Best guess".

In the peptide example, the consensus biotype suggests that the origin of the peptide could be either Introns or SINE repeat biotypes, as they have an equal likelihood. Therefore, in this case, the "Best guess" is reported as both having an equal probability, as shown in Figure 3a.

The second consensus biotype is generated by aggregating the reads assigned to each biotype and distributing them according to the biotype coefficients acquired through EM at each genomic location. The process for generating the consensus and "Best guess" follows the same rules as the first consensus biotype above.

However, for the peptide example, the weighted consensus biotype suggests that the peptide most likely derives from the intronic region rather than the ERE, as shown in Figure 3b. This approach provides an additional level of analysis and allows for a more informed determination of the peptide's biotype based on the expression levels of each biotype across all genomic locations and samples studied.

Figure 3. Consensus biotype for the **AEAGGSPEV** peptide. (A) Consensus biotypes are calculated according to the frequencies of biotypes at all genomic locations. All biotypes have the same weight in the consensus calculation. B) Consensus biotype calculated according to the biotype coefficients acquired with MS at all genomic locations.

To ensure users can make an informed decision regarding biotype attribution for the tested MAPs, we provided multiple outputs containing all functional annotations (biotypes). However, we want to emphasize that the biotype attributions reported by BamQuery are predictions based on RNA expression data only. This limitation is discussed in detail in the manuscript.

For more specific examples of the various outputs that BamQuery generates for each MAP, please refer to this link: <https://bamquery.iric.ca/documentation/examples.html#res>.

Regarding the comment on degraded ERE sequences, we want to first mention that if these degraded sequences can code for the peptide examined, they will be found by BamQuery, no matter if they are annotated or not (BamQuery locates the genomic regions independently of genomic annotations, see our responses to previous comments). Next, BamQuery utilizes the RepeatMasker database to annotate the genomic regions of ERE MAPs. RepeatMasker is a tool developed to detect and label repetitive elements in the genome. These annotations encompass not only younger repetitive elements but also other libraries of repeats such as Repbase. Repbase (PMID: 16093699; <http://www.girinst.org/>) is the largest repeat curated library, featuring expanded and enhanced consensus sequences for already known (older) repetitive families, as well as for new biological data. Therefore, BamQuery addresses older and new repetitive sequences, as long as they are included in the RepeatMasker annotations.

As pointed out by the reviewer, numerous ERE sequences may have become inactive due to sequence degradation, resulting in their inability to generate stable proteins. However, MAPs can still arise from these sequences through DRiPs (defective ribosomal products), which are truncated or misfolded proteins resulting from translation errors stemming from alternative/defective RNAs (PMID: 24532645). Hence, even if ERE sequences cannot produce stable proteins, they can still generate MAPs through DRiPs.

7. The statement was made that 'Canonical MAPs essentially derive from a single genomic location (60%)', but it is not clear why this was referred to as 'essentially' as 60% is just over half of the peptides, as 40% of canonical MAPs can be derived from multiple genomic locations. The percentages of ncMAPs that are derived from a single genomic location should also be noted.

**Response to major concern #7:**

We thank the reviewer for this comment. It is true that the use of the word essentially does not adequately reflect the 60% of peptides derived from a single genomic location. Therefore, we have changed the wording of this sentence and included a supplementary figure (Fig. S6d, Figure below) in the revised version of the manuscript to show the number of genomic locations for canonical and noncanonical MAPs.

As suggested, we included the number of noncanonical peptides derived from single genomic locations as follows:

“Canonical MAPs are primarily derived from a small number of genomic locations, with 63% originating from a single genomic location. In contrast, non-canonical MAPs can be derived from multiple regions, with only 43% originating from a single genomic location (Fig. 3e, Fig. S6d).”

Number of genomic locations from which canonical and non-canonical peptides could originate. The red line represents the mean of 15 and 2,356 genomic locations for canonical and non-canonical peptides, respectively.

8. It is not clear why there is more homogenous read coverage of intronic and ERE MAPs, as shown in Extended data Fig. 3a when previous results shown in Fig. 3b indicate that the coverage of the 5' and 3' ends of canonical MAPs is nearly identical.

**Response to major concern #8:**

We thank the reviewer for this comment. The results shown in former Extended data Fig. 3a and Fig. 3b (now Fig. S7a and Fig. 4b, respectively) are radically different types of analyses, explaining the counter-intuitive observation pointed out by the reviewer. Indeed, in Fig. S7a, we show a bias toward the 3’ end of transcripts for the read coverage. This is due to the chromium technology of 10x Genomics used to perform scRNA-seq. This method uses polydT primer located on the gel bead oligo for reverse transcription, thereby favoring the coverage of the 3’ end of transcripts. The bias observed for canonical transcripts in Fig. S7a is therefore expected. In contrast, in Fig. 4b, we show no bias toward the 3’ end of transcripts. This observation is due to the analysis performed in Fig. 4b itself. Indeed, here we only checked whether a peptide could be detected in the 3’ or 5’ sides of transcripts. Here, “detected” means: “Is the read count equal to zero or not?”. As shown in extended data Fig. S7a, the read coverage on the 5’ end of transcripts is lower than on the 3’end but not null. Therefore, Fig. 4b shows we can still detect (RNA expression different from zero) MAPs on the 5’end of transcripts. We have clarified this in the manuscript (page 13).

Regarding the more homogenous read coverage in introns and EREs than in canonical genes, we could not find an explanation for this observation. We could only speculate that it either results from the shorter length of non-canonical transcripts or from the technology itself. We consider elucidating this difference’s origin beyond the scope of our article.

9. In Figure 3f and 3g the results of probing the heterogeneity of intra-tumoral TAs carried out using BAMQuery were presented, but it would be informative to compare these results with those obtained using established pipelines.

We thank the reviewer for this comment. As requested, we have performed a new analysis of the tumor heterogeneity by using an established pipeline: UMAP+k-nearest neighbors clustering. In brief, we performed a clustering of tumor cells based on the expression of protein-coding genes and identified five clusters of cells (Figure 1 below), hereafter referred to as clones. We compared the expression of the 45 MAPs which were identified as overexpressed by tumor cells in our original differential expression analysis (Fig. 4d in manuscript) between tumor clones. We identified that MAPs’ expression is strongly associated with 3 specific groups of tumor cells, namely clones 1, 2, and 3 (Figure 2 below).

Figure 1. UMAP showing lung cancer cell clusters based on gene expression.

Figure 2. Barplot depicting the number of MAPs identified as overexpressed in each cluster of cancer cells by a differential expression analysis with MAST. Color indicates the biotype of each MAP.

Interestingly, 40 of the 45 MAPs were identified as differentially expressed between tumor clones, confirming our results that MAPs’ expression present extensive heterogeneity in tumor cells. We provide two representative examples of this differential expression of MAPs between tumor clones: FPEELTQTF overexpressed by clone 1, and KTYGEIFEK overexpressed by clones 2 and 5 (Figures 3-4 below). Finally, we compared the results obtained by our previous methodology (Fig. 4f) and this one to determine if they are consistent. Ten of the fourteen MAPs included in the clusters 1 and 2 of Fig. 4f were identified as differentially expressed between tumor clones. Interestingly, all MAPs from cluster 1 in Fig. 4f are overexpressed by tumor clone 2 whereas MAPs from cluster 2 (Fig. 4f) are associated to clones 1 and 2, confirming their co-expression in tumor cells (Supplementary Table 5). The overlap between this new method and our original results has been added to our manuscript (page 14).

Figure 3. The expression of **FPEELTQTF**, identified as overexpressed in cancer cell cluster 1 is projected on the UMAP lung cancer cells (Left-panel) or represented on a violin plot for each cluster of cancer cells (Right-panel).

Figure 4. The expression of **KTYGEIFEK**, identified as overexpressed in cancer cell clusters 2 and 5 is projected on the UMAP lung cancer cells (Left-panel) or represented on a violin plot for each cluster of cancer cells (Right-panel).

10. When referring to the mutant RYLAVAAVF peptide and its wild-type counterpart it not clear why this would be a promising target, as expression of the mutant peptide was not detected whereas the wild type peptide was detected, which of course would not represent a good target.

**Response to major concern #10:**

The RYLAVAAVF peptide was the only mutated MAP detected in the immunopeptidome of primary colorectal cancer (CRC) tissues in the study of Hirama et al^10^ (this is now specified clearly in our manuscript, page 15). It is, therefore, a tumor antigen. We sought to assess whether this peptide could be presented in normal tissues, thereby inducing auto-immune disorders if targeted with a vaccine. The BamQuery analysis shown in Fig. 5b evidenced a completely absent expression of this peptide in all normal tissues. Hence, it is a promising target since it is presented by colorectal cancer cells (verified by mass spectrometry in Hirama et al^10^) and cannot be presented (since not expressed at RNA level) in the normal tissues of GTEx. Therefore, it could be targeted with minimal risks of causing off-target toxicity. Our aim with this analysis is to demonstrate the usefulness of BamQuery to discriminate Tumor-Specific Antigens (TSAs) among uninteresting MAPs based on their expression patterns.

11. An explanation should be provided as to why BAMQuery able to detect ~66 locations per ERE-derived MAP whereas a previous study was only able to identify 8. A discussion of comparisons between the methods is merited given the wide disparity between the 2 results.

**Response to major concern #11:**

In their study, Bonaventura et al. attempted to identify tumor-specific HERVs (=EREs) generating MAPs (which would automatically be tumor-specific). They have performed large-scale transcriptomic analyses on cancer and normal samples and selected all HERVs highly overexpressed by cancer samples. Critically, the HERV annotations used in that article to quantify HERV transcripts (with a tool similar to Kallisto, HERVquant, PMID 30137025) came from a study focussed on discovering complete proviral sequences (PMID 26800882). Therefore, this database could be missing many “incomplete” HERV sequences, which could nevertheless code for MAPs. Next, they selected the HERVs whose expression is associated with cytotoxic T-cell signatures in cancer samples to obtain a list of tumor-specific and immunogenic HERVs. Next, they performed an in silico 6-frame translation of their HERVs, selected the protein sequences having a 90% homology with known retroviral proteins (HERV-K/HML-2 Gag and Pol), and performed predictions of HLA-A*0201 binding capacities on the obtained protein sequences. This yielded a list of HERVK MAP candidates. Finally, they performed mass spectrometry analyses to confirm the existence of these peptides within the proteome/immunopeptidome of cancer samples. Due to this targeted approach, they did not consider the possibility that other HERVs than their target sequences in the genome could code for the MAPs identified. Their article reports that their HERV MAPs can come on average from eight different HERVs in the PMID 26800882 database (Table S4 of Bonaventura et al.), which can code for the MAPs.

BamQuery performs the reverse translation of peptides and aligns the peptide-coding sequences on the genome to find all regions able to code for the peptide. Therefore, this approach is the opposite of a targeted approach since it performs its analyses at the genome level independently of any annotations. BamQuery evidenced a dramatically higher number of coding regions than the ~8 originally reported for each HERV MAP. Upon examining the biotypes (e.g., introns/ncRNA/coding exon/ERE/…) of the source regions identified by BamQuery, we observed that most of them were annotated as LTR (category to which HERVs belong). Therefore, our results suggest that the greater number of regions observed by BamQuery results from repetitions of the HERVs across the genome. Bonaventura et al. did not assess these repetitions because their approach was restricted to a limited number of genomic positions. To illustrate the highly repetitive nature of HERVs, we evaluated the number of repeats of each HERV-K class collected in the repeat masker database. We found that, on average, HERVK sequences have 253 repeats (regions) across the genome (Figure 1 below). We have added these data to our manuscript and have added more discussion of these results (page 16), as requested by the reviewer.

Figure 1. Number of repeats of each HERV-K class collected in the repeat masker database.

12. It is not clear for DLBCL whether tumor specific antigens also include mutated antigens. It is not clear why RYLAVAAVF why this represented the only mutated peptide analyzed in this manuscript.

**Response to major concern #12:**

First, we wish to mention that this peptide is not the only mutated peptide analyzed in our manuscript. In Fig. S9a-b and Fig. 5c (figures already present before revisions), we have analyzed 45 mutated MAPs (7 from gene fusions, 28 from aberrant splice junctions, and 10 from single nucleotide variations) identified in medulloblastoma. Furthermore, in Fig. 5d (figure also already present before revisions), we analyzed 393 mutated peptides collected from different reference databases of cancer neoantigens. Our conclusions for these two analyses were that many mutated peptides could also be coded by non-mutated genomic regions, sometimes highly expressed in normal tissues. These results question the validity of the “neoantigen” status of these MAPs, suggest that targeting them with immunotherapies could cause off-target toxicities, and demonstrates the usefulness of BamQuery to attribute a genomic origin or tumor-specificity to a given MAP. We have added multiple other analyses focused on the mutated peptides to our manuscript (pages 7-8+Fig 1g; page 14+Fig S8f-h; page 18+Fig S10c-d).

In response to the reviewer’s request, there were indeed mutated MAPs among our final list of TSAs. We first did not mention this information because these peptides were rare in our results. Indeed, 5 out of 67 mutated peptides (7%) were among our TSAs (Figure 1 below). One was highly shared among patients (NIPSHLPQV); see Figure 2 below. We have added these data and figures to our manuscript (page 19+Fig S10b-c).

Figure 1. Proportion of mutated MAPs among DLBCL TSAs.

Figure 2. Inter-patient sharing of the RNA expression of the mutated TSAs identified from DLBCL cell lines.

There were also several general issues in regarding the definitions of terms used in the manuscript, grammatical errors and other points that should be addressed.

1. It is important to be precise when classifying peptides into their biotypes (tumor antigens (TAs), ncMaps, etc).  When introducing 'tumor antigens' (TAs) in the first paragraph of the introduction, a reference was made to '…mutated tumor antigens (TAs)…'.  This implies that all tumor antigens discussed in the manuscript will be mutated when in fact most of them are not.

**Response to minor concern #1:**

We apologize for this confusion and agree with the reviewer. This has been corrected/clarified in the manuscript as shown hereafter:

“In recent years, immunopeptidomic data has been harvested to identify relevant and targetable tumor antigens (TAs). Indeed, MAPs deriving from mutations characterizing the neoplastic transformation (mutated TAs, also known as neoantigens) can be recognized by cytotoxic T cells and used as anti-cancer therapeutic targets.”

2. The term 'Biotype' should be defined when it is first mentioned on in the introduction on page

**Response to minor concern #2:**

We thank the reviewer for pointing this out to us. We have now clarified in the introduction that the biotype is a classification given to each MAP based on the annotations that overlap their genomic locations, as follows:

In Introduction:

“Second, the attribution of a biotype to MAPs corresponds to their annotation in terms of available genomic features that overlap with their genomic locations.”

And in the result part, in the fifth step of the BamQuery pipeline:

“…and (5) attribution of biotypes to MAPs that classify them in terms of genomic features in reference annotations that overlap with the various expressed regions (RPHM>0).”

3. There are several cases where either 'MAP' or 'MAPs' are the grammar in terms of matching the tense to the sentence are incorrect or the wording is awkward such as in the statement on page 4 '… comparing reliably MAPs RNA expression …'. There are also other instances where the case does not match the noun. For example, '…residues peptide…' should either be changed to '…residue peptide…' or '…peptides of 8-11 residues…'.

**Response to minor concern #3:**

We thank the reviewer for pointing this out to us. We have made the requested changes and have checked our manuscript for similar errors.

4. There are examples of incomplete sentences that need to be altered to contain verbs. These include 2 sentence fragments in the introduction: 'First, the attribution of an exact RNA expression to MAPs' and 'Second, the attribution of a biotype to MAPs.'.

**Response to minor concern #4:**

We thank the reviewer for pointing this out; we have corrected these sentences and checked our whole manuscript for similar errors.

5. Terms such as 'almost perfect' should be avoided, as when reporting correlations of different analysis tools.

**Response to minor concern #5:**

We agree with the reviewer. Such terms are no longer used.

6. On Page 10, line 51 'of' should be inserted before >5%.

**Response to minor concern #6:**

We thank the reviewer for pointing this out. We have made the change.

7. On page 16, line 16, the use of the term 'important' to describe the fraction of canonical MAPs is subjective and should be replaced with another term.

**Response to minor concern #7:**

As per reviewer 1’s recommendation, we have replaced the word "important" with the word "substantial".

The final sentence of the revised version of the manuscript is as follows:

“Strikingly, a substantial fraction of the canonical MAPs (~30%) could also be translated, with a greater probability, from non-canonical regions.”

8. On page 12, line 58 the meaning of the phrase  '…being retained as being lowly expressed…' is unclear.  Does this refer to DLBCL, GTEx, or benign B-cells?

**Response to minor concern #8:**

We regret the confusion introduced by this.

We have changed the phrasing to indicate that MAPs retained at that stage refer to MAPs poorly expressed in all normal samples, which include GTEx and benign B cells.

“To discriminate MAPs at risk of causing off-target toxicity when targeted, the remaining MAPs (14%) were queried in normal samples including GTEx and sorted benign B cells^37, 46^. Through this process, we retained only 5% of the queried MAPs, as they demonstrated poor expression levels in these normal samples (<8.55 RPHM).”

9. The differences between 'Peptide Mode' and 'MCS Mode' should be clarified. Also, as the second and third modes were not evaluated or described in this manuscript (or elsewhere), these should be evaluated in terms of their effects on the BAMQuery algorithm.

**Response to minor concern #9:**

We thank the reviewer for this comment. The difference between these two modes was indeed missing in our manuscript. These are different acquisition options offered by BamQuery. We have introduced more details in the Methods section as follows:

“A) Peptide mode: only the amino acid sequence of the MAP is provided by the user. BamQuery performs a reverse-translation of the peptide sequence and comprehensively searches all possible coding sequences across the genome. All results reported in the present article were obtained with this mode.

B) MAP coding sequence (MCS) mode: the nucleotide sequence of the MAP is provided in addition to the peptide sequence by the user. Hence BamQuery only searches for the genomic regions coding exactly for this MCS, and the expression of the peptide will be restricted to this MCS only.”

The MCS mode was implemented to increase the versatility of BamQuery. In addition to these two modes, BamQuery has a “manual” mode where the region is provided in addition to the peptide and MCS. The mention of this mode has also been added to our manuscript.

10. There are examples where some additional editing may improve the flow of the manuscript. For examples, the 3 aims in the introduction could be combined into 1 paragraph as the second aim is essentially a single sentence. In addition, the second aim, stated as ' Due to the multiplicity of genomic regions able to generate the same MAP, and possibly having different biotypes, a MAP could be mislabeled for example as ERE-derived while a canonical region could also generate it through out-of-frame translation.' is confusing and should be reworded.

**Response to minor concern #10:**

We thank the reviewer for this suggestion; we have edited this part of our manuscript to clarify it as shown hereafter (paragraphs were merged, as suggested):

“The presence of multiple genomic regions that can produce identical MAPs, and exhibit different biotypes, can lead to misidentification of their origins. For instance, a MAP may be attributed an ERE origin, while a canonical exon could also generate it through out-of-frame translation, possibly with a greater probability.”

11. Parts of Figure 2 (panels B, C, and D) showing codon composition do not add substantially to the overall message of the manuscript but could be mentioned briefly and included in the supplemental data.

**Response to minor concern #11:**

We kindly disagree with the reviewer as we feel that our manuscript is not only focused on tumor antigens but also on the new biologic insights provided by BamQuery. This observation will interest researchers working on the biology of the immunopeptidome. This is notably supported by many questions that we received about these results during the presentation of BamQuery at the 2023 AACR annual congress. We have therefore left these panels in the figure, now Fig. 3.

12. The text in the main figures is too small and can only be read when it is blown up on the screen and so the font size should be increased for these figures.

**Response to minor concern #12:**

We agree with the reviewer and have increased the font size in all the figures to improve readability. We have also refined our figures to optimize the size of panels.

**Reviewer #2:**

The manuscript describes an approach to infer the expression of MHC-I associated peptides coming from both coding and non-coding genomic regions. It is based on the idea of counting for each peptide the number of occurrences of each RNA sequence that could encode the peptide in RNA-Seq data. This has an advantage compared to standard approaches that consider only protein-coding genes, or predict MAP expression based on the expression of full genomic region(s) where the MAPs come from. The authors then analyzed expression of possibly cancer-specific MAPs in the thymus and/or benign tissues to shed light on whether some of these MAPs may actually be explained be found in such tissues by expanding the search space to non-canonical regions.

We thank the reviewer for the time dedicated to revising our article. Her/his comments have greatly contributed to improving our manuscript.

**Major comments:**

-Figure 1B: It still seems that several MAPs have predicted count of 0 (or very low) with BamQuerry, but have > 50  counts in the k-mer database. How can this be? Does it mean that many MAPs would be predicted to have very low expression, when this is actually not the case?

**Response to major comment #1:**

We thank the reviewer for pointing this out. While investigating for these differences’ causes, we noticed that almost all the MCS for which a large-scale difference between the k-mer database and BamQuery was observed coded for the same peptide, RVHPQVTVY. This peptide is located in a hypervariable region of the HLA-DRB3 MHC gene. Due to the great natural genetic variation in MHC genes, the STAR aligner presents limitations, already observed before^11^, to perform read alignments in these genes. Therefore, the disparity between the k-mer database results and BamQuery was mainly due to a single peptide coded by many highly variable MCS. When aggregating the MCS expression into peptide expression (the abundances of all MCS coding for the same peptide were summed together), the correlation was greatly improved, as shown below. We have replaced the Fig. 1b with this one and have moved the old Fig. 1b to supplemental data (Fig. S2a). We have also modified the text in the manuscript (page 6):

“The comparison of total read counts between BamQuery and total k-mer occurrences for each MAP showed a correlation equal to 1, demonstrating the exhaustivity of BamQuery (Fig. 1b). Importantly, the main outlier in this correlation was the RVHPQVTVY peptide, deriving from the HLA-DRB3 gene. Previously, the STAR aligner was shown to have poor performance in hypervariable genomic regions such as HLA genes ^11^. Consequently, this outlier results from the limited capacity of STAR to map MCS to the HLA-DRB3 gene when performing the BamQuery analysis. A more detailed comparison between MCS counts given by BamQuery and k-mer counts in the database also showed an excellent correlation, except for the MCS coding for the RVHPQVTVY peptide (Fig. S2a).”

Pearson’s correlation between BamQuery-acquired read counts and Jellyfish’s K-mer counts for canonical nonamer MAPs (n=1,211) from the HLA Ligand Atlas (present in at least 20 different tissues) in eight mTEC samples.

- Figure 1C: It seems that Kallisto actually failed for only 32 out of 1211 MAPs. This is not so bad, considering that Kallisto may discard many of the reads coming from non-coding regions. More generally, I find the comparison between BamQuery and existing tools quite limited. This should be expanded to demonstrate the improvements brought by BamQuery over other approaches.

**Response to major comment #2:**

We thank the reviewer for this comment. Before responding to the reviewer’s comment, we wish to mention that while investigating the origin of the missed expression of these 32 peptides by Kallisto, we noticed two sources of bias in our original Kallisto analysis. First, some peptides provided by the HLA Ligand Atlas (the source of these peptides: <https://hla-ligand-atlas.org/>) were not mapped properly to all proteins able to code for them by the atlas team. Therefore, our original Kallisto quantifications were not considering all protein-coding genes able to code for each assessed peptide. Second, the annotations used by the Atlas team for these proteins are from Uniprot, and the annotations used in our Kallisto analyses are from Ensembl. Because these two types of annotations are too different (e.g., multiple Uniprot annotations do not have matched Ensembl annotations, and vice versa), the attribution of source genes to each peptide of the atlas in our original analysis could lack precision. These two sources of bias made us wonder whether we could improve the analysis shown in previous Fig 1c. Therefore, we repeated this analysis by using a different method, independent from the atlas annotations, detailed hereafter: (1) each of the 1702 peptides was queried in the reference proteome of Ensembl to obtain the full list of genes able to code for each peptide; (2) the Kallisto transcript-level quantifications were converted to gene-level quantifications by using the Ensembl annotations and the tximport R package; (3) the Kallisto TPM expression of all genes able to code for the same peptide was summed; (4) Kallisto TPM data were compared to BamQuery RPHM data of the same peptides (data shown in new Fig. 1c).

This new analysis showed that Kallisto missed no peptides, so our previous statement that Kallisto missed 32 peptides is no longer valid. This change is due to a better annotation of the source genes of each peptide, as detailed above. Instead, we found good correlations (~0.7 for single samples and 0.51 when averaged all together) between Kallisto and BamQuery results for these peptides (new Fig. 1c-f, Figure 1 below).

Figure 1. **(A)** Correlation between BamQuery’s (in RPHM) and Kallisto’s (in TPM) quantifications of 1,702 MAPs from the HLA Ligand Atlas in 8 mTEC samples. Because Kallisto does not perform direct quantifications of MAPs’ RNA expression, the expression of their gene of origin was used as a surrogate. A value of 0.5 was added to each RPHM or TPM value to enable visualization on a logarithmic axis. Correlations for three representative samples and the average of the eight samples are shown. **(B)** Segregation of MAPs based on their number of coding regions and re-computation of correlations for the average of 8 mTEC samples. **(C)** Box plots of the average expression of MAPs in the eight mTECs, segregated based on the number of coding regions. **(D)** Correlation between the ratio, for each MAP, between the BamQuery and the Kallisto quantification (average of 8 mTECs), as a function of the number of coding regions.

As pointed out by the reviewer, Kallisto’s results are not so bad! However, this result is expected since Kallisto is designed to evaluate the expression of protein-coding transcripts, and the similarity demonstrates the accuracy of BamQuery (for a reminder, all MAPs reported in the HLA ligand atlas were identified by mass spectrometry, based on protein-coding gene annotations). However, we noticed that the correlation between Kallisto and BamQuery decreased for MAPs deriving from multiple genomic regions, and the difference between BamQuery and Kallisto’s results grows with the number of regions of origins (Figure 1B above). Here, the BamQuery (RPHM) / Kallisto (TPM) values increase with the number of regions, showing that Kallisto fails to capture an increasing amount of RNA expression with the increase of coding region numbers. Since Kallisto does not assess non-coding regions, while BamQuery does, this increasing difference results from the expression of non-exonic regions. Importantly, these MAPs coded by numerous regions presented a significantly greater expression than those coded by fewer regions, showing that Kallisto’s imprecision is greater for highly expressed peptides (therefore, more likely to be presented widely in normal tissues). These results were added to our manuscript (page 7).

Because there were few MAPs with high numbers of regions in our original dataset, we built another one from the HLA Ligand Atlas. In brief, we performed a 3-frame translation of the genomic regions overlapping EREs and queried all the HLA Ligand Atlas epitopes in that database. It returned a list of 1126 MAPs that we analyzed with BamQuery and Kallisto. Identical results were found (Figure 2 below). The correlation between BamQuery’s and Kallisto’s quantifications decreased for MAPs coded by many regions. The MAPs coded by multiple regions were more expressed than the others, and the ratio BamQuery (RPHM) / Kallisto (TPM) increased with the number of coding regions. These results were added to our manuscript (page 7).

Figure 2. Comparison between BamQuery and Kallisto’s quantifications in 8 mTEC samples for 1126 MAPs from the HLA Ligand Atlas also overlapping genomic regions of EREs (see methods for selection criteria). Because Kallisto does not perform direct quantifications of MAPs’ RNA expression, the expression of their gene of origin was used as a surrogate. A value of 0.5 was added to each RPHM or TPM value to enable visualization on a logarithmic axis. Correlations for three representative samples and the average of the eight samples are shown. (**A**) Correlation on the average of eight mTECs samples for three groups of MAPs segregated as a function of their number of source genomic regions, determined by BamQuery. (**B**) Box plots of the average expression of MAPs in the 8 mTECs, segregated based on the number of coding regions. **(C)** Correlation between the ratio, for each MAP, between the BamQuery and the Kallisto quantification (average of eight mTECs), in function of the number of coding regions.

In addition, some peptides presented divergent quantifications between the two methods, with expression values lower with BamQuery than with Kallisto (Figure 1 above). Upon manual evaluation of these peptides, we found that most of them mapped to genomic regions bearing mutations in the mTECs samples examined. Fig. S3 (Figure 3 below) shows the genomic region of three of these peptides that we used to illustrate the source of these divergences. These examples are detailed hereafter: the VHLTPEEKSAV peptide (as well as its subpeptides HLTPEEKSAV and VHLTPEEKSA) derives from the hypervariable region of the hemoglobin subunit beta gene (HBB). The Kallisto quantification of the HBB gene reported an expression of 98.48 TPM for this peptide in the S10 sample. Kallisto only counts all reads mapping to the gene body of the HBB gene, without considering local allelic variations which can affect the amino acid sequence of its protein product. In contrast, BamQuery successfully captured this variation, and returned a count of 0 RPHM in that sample (S10), showing that the sample did not express the alleles enabling the expression of this peptide (a mutation is clearly visible in orange in all examined samples). Similar results were obtained for the EVVDKINQV peptide (which prevented its expression in the S11 sample). The YQLELRTTL peptide is a variant of the YEAELRTTL peptide that could be expressed only by the S11 sample presenting the required alleles in the HLA-DB2 gene. Altogether, these results demonstrate the efficacy of BamQuery at detecting the exact expression of a given peptide.

Figure 3. IGV (genome browser) view of the coding regions of three MAPs for which large differences were observed between BamQuery’s and Kallisto’s quantifications. Each of these MAPs overlapped annotated mutations. The read coverage is shown for each of the three mTEC representative samples (light blue/red), and the positions of dbSNP annotations are shown at the bottom of the plot.

To further demonstrate the superiority of BamQuery at detecting the exact expression of peptides over methods such as Kallisto, we queried the expression of 108 minor histocompatibility antigens (**miHAs**, reported before in PMID 26857467) in the eight mTECs samples tested. These MAPs derive from non-synonymous germline (benign) mutations recognized as immunogenic by allogeneic MHC-matched T cells in hematopoietic cell transplantations. BamQuery could attribute an exact (matching the location reported in the original publication) genomic location to 96% of miHAs. Here, the correlations between BamQuery and Kallisto were very poor, with Kallisto overestimating the expression of many miHAs (Figure 1 below). Three examples of peptides are shown in Fig. S4 (Figure 2 below). For each of these peptides (ALSGHLETL, AELKGFHRSF, RVLQEQHQL), Kallisto detected a high expression (100-1000 TPM), but BamQuery detected an expression of 0 RPHM in the mTEC samples that did not express the adequate allele to code for these peptides. As mentioned above, this overestimation is due to the method used by Kallisto to quantify the expression of genes: by counting reads mapping to the gene body without considering local allelic variations. The number of miHAs having an expression =0 was significantly greater with BamQuery than with Kallisto (Figure 1B). Together with the impossibility of quantifying unannotated regions with Kallisto (preventing the analysis of non-canonical MAPs, so ~10% of MAPs and currently promising tumor-specific targets), these data further demonstrate the versatility and superiority of BamQuery over other methods for assessing the expression of MAPs. These results were added to our manuscript (page 8).

Figure 1. (A) Correlation between BamQuery’s (in RPHM) and Kallisto’s (in TPM) quantifications of 108 minor histocompatibility antigens in 8 mTEC samples. Because Kallisto does not perform direct quantifications of MAPs’ RNA expression, the expression of their gene of origin was used as a surrogate. A value of 0.5 was added to each RPHM or TPM value to enable visualization on a logarithmic axis. Correlations for three representative samples and the average of the eight samples are shown. (B) The number of miHAs having an expression =0 according to Kallisto or BamQuery is reported in each of the eight tested mTEC samples (dots).

Figure 2. IGV (genome browser) view of the coding regions of three miHAs for which large differences were observed between BamQuery’s and Kallisto’s quantifications. Each of these MAPs overlapped annotated mutations. The read coverage is shown for each of the three mTEC representative samples (light blue/red), and the positions of dbSNP annotations are shown at the bottom of the plot. The figure shows a high read coverage (measured by Kallisto) overlapping the region of the target peptides, suggesting erroneously high expression of the peptide if relying on Kallisto. However, BamQuery counts the exact number of reads able to code for the peptide locally, identifying which samples express the alleles necessary for the expression of the mutated peptide. For the ALSGHLETV peptide in the S11 sample, the allelic frequency of the reads coding for the peptide was too low to be highlighted in color by IGV.

Altogether, we believe that these new analyses better illustrate the interest of BamQuery, as well as expand the comparison between BamQuery and existing tools, as per the reviewer’s request. Please also see our response to major concern #4 of reviewer 1 for a discussion of the advantage of BamQuery over k-mer database queries (another method used to benchmark BamQuery).

- In Figure 2, it could be useful to know whether the reported results are specific for MAPs, and whether the same results would be observed for any peptide encoded by canonical/ncRNA/Intronic/ERE elements.

**Response to major comment #3:**

We thank the reviewer for this question. To obtain a set of peptides similar to those of Fig. 2 (now Fig. 3), and not being MAPs, we have extracted from the reference genome the contiguous 27-nucleotide sequence of each region able to code for the peptides of Fig. 3 (two contiguous sequences were extracted for each peptide: one upstream and one downstream). Then we translated these sequences into peptide sequences, removed peptides containing stop signals, and performed netMHCpan predictions of their capacity to bind all known MHC-I alleles. We kept only the peptide sequences predicted as non-MHC binders, yielding a list of 14,810 peptides. This high number is due to the fact that some EREs mapped to many genomic regions, and therefore one ERE could generate many different upstream and downstream peptide sequences (often varying by a single amino acid from one peptide to another). To gather a dataset comparable in size to the dataset of Fig. 3, for each original MAP, we made random selections among the downstream and upstream non-binders peptides that were generated and assembled the downstream and upstream peptides into a single dataset. This yielded four lists of peptides: canonical (1,478), ncRNA (136), intronic (43), and ERE (185). These peptide numbers are not 2-fold higher than those of Fig. 3a (1,211, 207, 68, and 157, respectively), as could be expected because some MAPs generated neighbors with stop codons (that were discarded from the analysis) and the neighbors of others were all predicted to be MHC binders. Nevertheless, we believe this dataset is comparable to our original dataset of MAPs while being non-MHC binders since these peptides derive from the same genomic region as the MAPs shown in Fig. 3a.

Comparing the number of possible coding sequences showed no difference between the four groups of peptides (Figure 1 below). No enrichment for 6-codons-encoded amino acids was also observed (Figure 2 below). Altogether, these results show that our observations in Fig. 3 are specific to MAPs, in agreement with our assumption that the generation of DRiPs (which is involved in the biogenesis of MAPs) plays a significant role in these results. We have added these results to our manuscript (page 11).

Figure 1. Comparison of the number of possible MCS after reverse translation of a set of nonbinders of MHC molecules (peptides which are not MAPs, see methods for a description of their selection).

Figure 2. Average frequency (%) of amino acids encoded by the indicated number of synonymous codons in indicated groups of peptides which cannot be presented by MHC molecules.

-Figure 3: Although it is nice to see that BamQuery can in principle be applied to scRNA-Seq data, I'm wondering if it practically makes sense to look at each cell separately, and if it would not be better to combine the RNA-Seq data of cells of the same cluster/cell type. How much is the fact that some cells do not express a MAP the result of dropout?

**Response to major comment #4:**

We thank the reviewer for this question. While the high proportion of zero counts (zero inflation) that is observed in single-cell RNA-seq data has been variously attributed to technical artifacts (dropout) or to statistical sampling, a recent publication in Genome Biology showed that its origin is primarily biological (PMID 32718323). Therefore, we believe that addressing this question (contribution of technical dropout to absent MAP expression in a single cell) is beyond the scope of our article. Nevertheless, we agree with the reviewer that the expression of MAPs should be assessed at the cluster-level, instead of single-cell level. As for canonical genes, there is a high variability of MAPs expression within each cluster of cells, and assessing the expression per cell makes little sense given the zero inflation inherent to single-cell RNA-seq data. All analyses in our article were already made by cluster comparisons using MAST, a software considering both the level and frequency of expression within each compared cluster (PMID 26653891). To illustrate this recommendation, we now show in Fig. S7b the total lung cancer single-cell RNA-seq sample where we report the expression of the SSASQLPSK ERE MAP that we found to be differentially expressed in our current Fig. 4d analysis. This expression has been projected on a UMAP generated from the expression of canonical genes, as reported in the original publication of these data [PMID 29988129]. The absent expression of the peptide in multiple cells of the two clusters expressing it at the highest level (alveolar and cancer cells, see also the violin plot in Fig. S7c) is clearly visible. Therefore, the expression of the peptide should be considered at the cluster-level instead at the single-cell level. These data and considerations were added to the manuscript (pages 13-14).

-I think it would be nice to have some time evaluation of the tool. If all possible MCS need to be searched for hundreds of peptides, this could take some time.

**Response to major comment #5:**

We thank reviewer #2 for this suggestion. We have evaluated the time required by BamQuery to analyze Canonical and Non-canonical MAPs. To do so, we randomly sampled canonical (our initial dataset of 1702 MAPs from the HLA Ligand Atlas) and noncanonical (our initial dataset of 724 MAPs, Supplementary Table 1) MAPs to create ten subsets of 1, 2, 3, 4, 5, 10, 15, 20, 30, 50, 70, or 100 peptides (Figure 1 below). Using four cores, we ran BamQuery to report peptide expression and biotype in 8 mTEC samples. BamQuery required a median of 37 and 92 seconds to analyze canonical and non-canonical MAPs, respectively (Figure 2 below, left panel). The disparity in the time required by BamQuery to process canonical and non-canonical MAPs is explained by the fact that non-canonical peptides generate almost 4-fold more MCS than canonical peptides (Figure 2 below, right-panel).

Figure 1. Random sampling of canonical MAP peptides (1,702) and non-canonical MAPs (724) to create ten subsets of 1, 2, 3, 4, 5, 10, 15, 20, 30, 50, 70, or 100 peptides. The y-axis shows the total MCS of the peptides (red dots) and the time (blue dots) that BamQuery takes to process. Each dot shows the mean values and standard deviation as error bars.

Figure 2. Time required by BamQuery to analyze the expression in eight mTEC of ten randomly sampled subsets of 1 to 100 canonical and non-canonical MAPs peptides using a minimum capacity of four cores (left panel). Number of MCS generated per canonical and non-canonical MAP (right panel).

In addition, we investigated the impact of the number of cores on BamQuery's runtime. We analyzed 100 randomly selected canonical and non-canonical MAPs using 4, 8, 16, and 32 cores. As expected, the results showed that using 32 cores significantly reduced the runtime by two to three times compared to using only 4 cores (Figure 3 below).

Figure 3. Time required (min) by BamQuery to analyze the expression in eight mTEC of 100 randomly sampled canonical and non-canonical MAPs peptides using 4,8,16, and 32 cores.

These results were added to our manuscript (pages 8-9 and figure S5).

**Minor comments:**

- Page 3, line 34: I do not understand the logic. It is fully expected that the majority of ncMAP are not mutated (mutation represents only a very small fraction of the genome).

**Response to minor comment #1:**

We agree with the reviewer that mutations represent only a small portion of the genome. We realized that the contrast that we established between the fact that most mutations are outside the exome and the low frequency of ncMAPs identified so far is probabilistically not well supported. We have replaced this sentence with “So far, most of the discovered ncMAPs are non-mutated”.

- Page 4, line 9: Not sure the degeneracy of the genetic code is the reason why some MAP could map to different regions. To me, it seems more likely that most of the ambiguous MAPs could be due to paralogs/orthologs. This is also supported by Fig. 2f (some MAPs with a limited number of possible MCS are found in many more regions).

**Response to minor comment #2:**

We agree with the reviewer and have mentioned the possible role of paralogs/orthologs as an explanation for the multiple regions of origin. However, we did not remove the mention of the degeneracy of the genetic code since some MAPs can be coded by many different MCS in our results.

- Would be good to define what is meant by 'biotypes'.

**Response to minor comment #3:**

We have emphasized that the biotype is a classification given to each MAP based on the annotations that overlap their genomic locations, as follows:

In Introduction:

“Second, the attribution of a biotype to MAPs corresponds to their annotation in terms of available genomic features that overlap with their genomic locations.”

And in the result part, in the fifth step of the BamQuery pipeline:

“…and (5) attribution of biotypes to MAPs that classify them in terms of genomic features in reference annotations that overlap with the various expressed regions (RPHM>0).”

- Page 5, line 55: not sure 'benign MAPs" is the best word. A MAP cannot be benign/malignant.

**Response to minor comment #4:**

We have modified the text to emphasize that these MAPs originate from benign tissues.

“To test BamQuery, we collected robustly validated MAPs derived from benign tissues reported in the HLA Ligand Atlas18 (1,702 canonical MAPs shared across at least 20 tissues, Fig. S1b,c).”

- Figure 1A: I struggled to understand it. Would it not be simpler if only one peptide was used? Also, where does the 73'728 comes from (number of DNA sequences coding for this peptide?) I also suspect that the MCS mode could still lead to multiple mapping to the genome?

**Response to minor comment #5:**

We appreciate this comment. We have modified Fig. 1a and believe it is now more readable (Figure below).

Indeed, the 73,728 MCSs are from reverse translation (aa to DNA), as suspected, MCS from peptides in MCS mode will map to the genome, possibly resulting in several genomic locations for MCSs. We have clarified this in the Methods section:

“All MCSs provided for peptides in MAP coding sequence (MCS) mode are included in the same fastq file to facilitate the compilation of their genomic locations.”

Overview of the BamQuery approach to measuring MAPs RNA expression levels.

- Figure 1B and text: What is JellyFish (not mentioned in the main text). Also what is 'accuracy of 94%' for a correlation, with a Pearson coefficient of 0.97

**Response to minor comment #6:**

We have paraphrased the first section of the manuscript to introduce the Jellyfish tool and given more details in the Methods section.

“As a control, we used the primary reads contained in the mTEC bam files previously aligned with STAR to generate a database of 27-nucleotide-long k-mers (reads chunked into smaller sequences) using the Jellyfish^6^ tool that counts their occurrences in fastq sequences (Methods).”

In addition, as mentioned in reply to major comment #1, we have introduced a change in Fig. 1b. Instead of showing the correlation between BamQuery counts and Jellyfish k-mer occurrences for individual MCS (this figure was moved to the supplemental data, Fig. S1d), we now present the correlation for MAPs using total BamQuery counts and total Jellyfish k-mer occurrences.

**References**

1.	Pearson, H. et al. MHC class I-associated peptides derive from selective regions of the human genome. *J Clin Invest* **126**, 4690-4701 (2016).

2.	Bassani-Sternberg, M., Pletscher-Frankild, S., Jensen, L.J. & Mann, M. Mass spectrometry of human leukocyte antigen class I peptidomes reveals strong effects of protein abundance and turnover on antigen presentation. *Mol Cell Proteomics* **14**, 658-673 (2015).

3.	Chong, C. et al. Integrated proteogenomic deep sequencing and analytics accurately identify non-canonical peptides in tumor immunopeptidomes. *Nat Commun* **11**, 1293 (2020).

4.	Ruiz Cuevas, M.V. et al. Most non-canonical proteins uniquely populate the proteome or immunopeptidome. *Cell Rep* **34**, 108815 (2021).

5.	Ehx, G. et al. Atypical acute myeloid leukemia-specific transcripts generate shared and immunogenic MHC class-I-associated epitopes. *Immunity* **54**, 737-752.e710 (2021).

6.	Marcais, G. & Kingsford, C. A fast, lock-free approach for efficient parallel counting of occurrences of k-mers. *Bioinformatics* **27**, 764-770 (2011).

7.	Dobin, A. et al. STAR: ultrafast universal RNA-seq aligner. *Bioinformatics* **29**, 15-21 (2013).

8.	Bray, N.L., Pimentel, H., Melsted, P. & Pachter, L. Near-optimal probabilistic RNA-seq quantification. *Nature Biotechnology* **34**, 525-527 (2016).

9.	Putri, G.H., Anders, S., Pyl, P.T., Pimanda, J.E. & Zanini, F. Analysing high-throughput sequencing data in Python with HTSeq 2.0. *Bioinformatics* **38**, 2943-2945 (2022).

10.	Hirama, T. et al. Proteogenomic identification of an immunogenic HLA class I neoantigen in mismatch repair-deficient colorectal cancer tissue. *JCI Insight* **6** (2021).

11.	Ballouz, S., Dobin, A., Gingeras, T.R. & Gillis, J. The fractured landscape of RNA-seq alignment: the default in our STARs. *Nucleic Acids Res* **46**, 5125-5138 (2018).

**Second round of review**

**Reviewer 1**

The issues raised by the reviewers were adequately addressed in the revisions to the manuscript.

**Reviewer 2**

My comments have been addressed
